# Multicomponent Interdisciplinary Group Intervention for Self-Management of Fibromyalgia: A Mixed-Methods Randomized Controlled Trial

**DOI:** 10.1371/journal.pone.0126324

**Published:** 2015-05-15

**Authors:** Patricia Bourgault, Anaïs Lacasse, Serge Marchand, Roxanne Courtemanche-Harel, Jacques Charest, Isabelle Gaumond, Juliana Barcellos de Souza, Manon Choinière

**Affiliations:** 1 École des sciences infirmières, Faculté de médecine et des sciences de la santé, Université de Sherbrooke, Sherbrooke, QC, Canada; 2 Centre de recherche clinique Étienne-Le Bel du Centre hospitalier universitaire de Sherbrooke, Sherbrooke, QC, Canada; 3 Centre de recherche du Centre hospitalier de l’Université de Montréal (CRCHUM), Montréal, QC, Canada; 4 Département des sciences de la santé, Université du Québec en Abitibi-Témiscamingue, Rouyn-Noranda, QC, Canada; 5 Département de neurochirurgie, Faculté de médecine et des sciences de la santé, Université de Sherbrooke, Sherbrooke, QC, Canada; 6 Département d’anesthésiologie, Faculté de médecine, Université de Montréal, Montréal, QC, Canada; David Geffen School of Medicine, UNITED STATES

## Abstract

**Background:**

This study evaluated the efficacy of the PASSAGE Program, a structured multicomponent interdisciplinary group intervention for the self-management of FMS.

**Methods:**

A mixed-methods randomized controlled trial (intervention (INT) vs. waitlist (WL)) was conducted with patients suffering from FMS. Data were collected at baseline (T_0_), at the end of the intervention (T_1_), and 3 months later (T_2_). The primary outcome was change in pain intensity (0-10). Secondary outcomes were fibromyalgia severity, pain interference, sleep quality, pain coping strategies, depression, health-related quality of life, patient global impression of change (PGIC), and perceived pain relief. Qualitative group interviews with a subset of patients were also conducted. Complete data from T_0 _to T_2 _were available for 43 patients.

**Results:**

The intervention had a statistically significant impact on the three PGIC measures. At the end of the PASSAGE Program, the percentages of patients who perceived overall improvement in their pain levels, functioning and quality of life were significantly higher in the INT Group (73%, 55%, 77% respectively) than in the WL Group (8%, 12%, 20%). The same differences were observed 3 months post-intervention (Intervention group: 62%, 43%, 38% vs Waitlist Group: 13%, 13%, 9%). The proportion of patients who reported ≥50% pain relief was also significantly higher in the INT Group at the end of the intervention (36% vs 12%) and 3 months post-intervention (33% vs 4%). Results of the qualitative analysis were in line with the quantitative findings regarding the efficacy of the intervention. The improvement, however, was not reflected in the primary outcome and other secondary outcome measures.

**Conclusion:**

The PASSAGE Program was effective in helping FMS patients gain a sense of control over their symptoms. We suggest including PGIC in future clinical trials on FMS as they appear to capture important aspects of the patients’ experience.

**Trial registration:**

International Standard Randomized Controlled Trial Number Register ISRCTN14526380

## Introduction

Fibromyalgia syndrome (FMS) is a chronic disorder of unclear origin. Growing evidence suggests a combination of interacting neurophysiological, genetic, and psychosocial mechanisms as the cause of FMS [1,2]. This syndrome is characterized by widespread musculoskeletal pain in association with fatigue, poor sleep quality, cognitive dysfunction, mood disturbances, and many other variable somatic symptoms [3]. Prevalence of FMS in the general population varies from 1.0 to 4.9% in women and from 0 to 2.9% in men [1,3–6] as demonstrated by studies from Europe, USA and Canada.

There is currently no cure for FMS nor is there a “gold standard” of treatment. Management of this disorder is therefore aimed at reducing symptoms and maintaining optimal functioning [7,8]. Interventions such as medication alone or the use of a single non-pharmacological treatment produce, at best, modest effects on patients' condition [9,10]. Results of a meta-analysis of 49 studies published 15 years ago [11] suggest that non-pharmacological treatments are more effective than drug interventions. A recent meta-analysis of 23 studies assessing the efficacy of psychological interventions for fibromyalgia showed small to medium positive effects on short and long-term pain, quality of sleep, functional status, depression, and tendency to catastrophize in the face of pain [12]. Other recent literature reviews on the use of patient education, exercise activities, cognitive behavioural therapy (CBT), and multidisciplinary treatment [13–16] suggest that a multimodal approach which combines at least one educational/psychological intervention with at least one exercise treatment can be effective for improving FMS symptoms including pain, fatigue, mood and/or quality of life (QOL). However, many of the reviewed studies suffer from methodological deficiencies (e.g., small sample size, single site study, unstandardized outcomes, short follow-up, etc), and well-designed trials are still needed.

Based on the Interactional School of Low Back Pain [17,18], Barcellos de Souza et al. [19] developed in 2007 a multimodal group intervention—the Interactional School of Fibromyalgia (ISF)—which combines exercise therapy and educational/psychological tools for self-management of FMS. Patient empowerment is an integral component of the intervention as is active patient participation. The authors [19] conducted a randomized controlled trial (RCT) to assess the efficacy of their intervention and found positive effects on pain intensity and perceived overall capacity to manage FMS symptoms. Although promising, these results remain preliminary and need to be replicated in a RCT involving more than one site, and using a comprehensive set of well-validated outcome measures such as those recommended by the IMMPACT (Initiative on Methods, Measurement, and Pain Assessment in Clinical Trials) Group [20–22]. Furthermore, adding a qualitative research component to the study would be an asset to further capture the patients’ experience during the intervention. Finally, some aspects of the ISF needed to be updated and somewhat reorganized. We therefore adapted the ISF into a more structured intervention program entitled PASSAGE whose French acronym is *P* *rogramme d’* *A* *pprentissage de* *S* *tratégie* *S* *d’* *A* *uto-* *G* *estion* *E* *fficaces* (Training Program of Efficient Self-Management Strategies).

The aim of the present study was thus to evaluate, quantitatively and qualitatively, the efficacy of the PASSAGE Program—a multicomponent interdisciplinary group intervention for the self-management of FMS. It was expected that the Program will lead to improvements in the clinical condition of patients suffering from this disorder.

## Methods

The French version protocol for this trial (as well as the English translation of the Methods section) and supporting CONSORT checklist are available as supporting information; see S1 CONSORT Checklist, S1 and S2 Protocols.

### Ethics Statement

The research protocol of the present study along with the patient informed consent form were reviewed and approved by the *Comité d’éthique de la recherche sur l’humain du Centre hospitalier de l’Université Sherbrooke*, Sherbrooke, Quebec, Canada (May 26^th^ 2009, #09–034) and by the *Comité d’éthique de la recherche avec des êtres humains de l’Université du Québec en Abitibi-Témiscamingue (CÉR-UQAT)*, Rouyn-Noranda, Quebec, Canada (May 15^th^, 2009). The study was registered at the International Standard Randomized Controlled Trial Number Register #ISRCTN14526380 (http://www.controlled-trials.com/ISRCTN14526380/).

### Protocol and Adjustments

Six adjustments were made to the protocol prior to enrolment. First, the upper age limit (65 years) was withdrawn. Second, patients suffering from chronic pain disorders other than FMS (e.g., painful diabetic neuropathy) were not excluded from the study as long as the pain associated with FMS was their predominant complaint. Third, potential participants had to accept to not introduce new pain medications or other new therapeutic modalities for pain management during the 11 weeks of the intervention because such a change in treatment could have biased our estimation of the intervention efficacy and made difficult to isolate its effects). Fourth, an additional training session for the health care professionals acting as facilitators was conducted to clarify some issues, answer questions, review the procedures, and insure uniformity between study sites. To minimize costs, this session was conducted via video conference due to the large distance between the two study sites. Fifth, research assistants were instructed to calculate the participants' scores on the Beck Depression Inventory (BDI) upon reception of their questionnaire. If a score > 30 was found and/or that participant reported suicidal ideas (question 9 of the BDI), the research assistant was instructed to contact the patient by phone and encouraged him/her to make an appointment with his/her treating physician (or psychologist) or to go the hospital emergency department. Finally, focus groups were added to the research protocol in order to document and further capture the patients’ experience.

### Design and Settings

**Fig 1:**
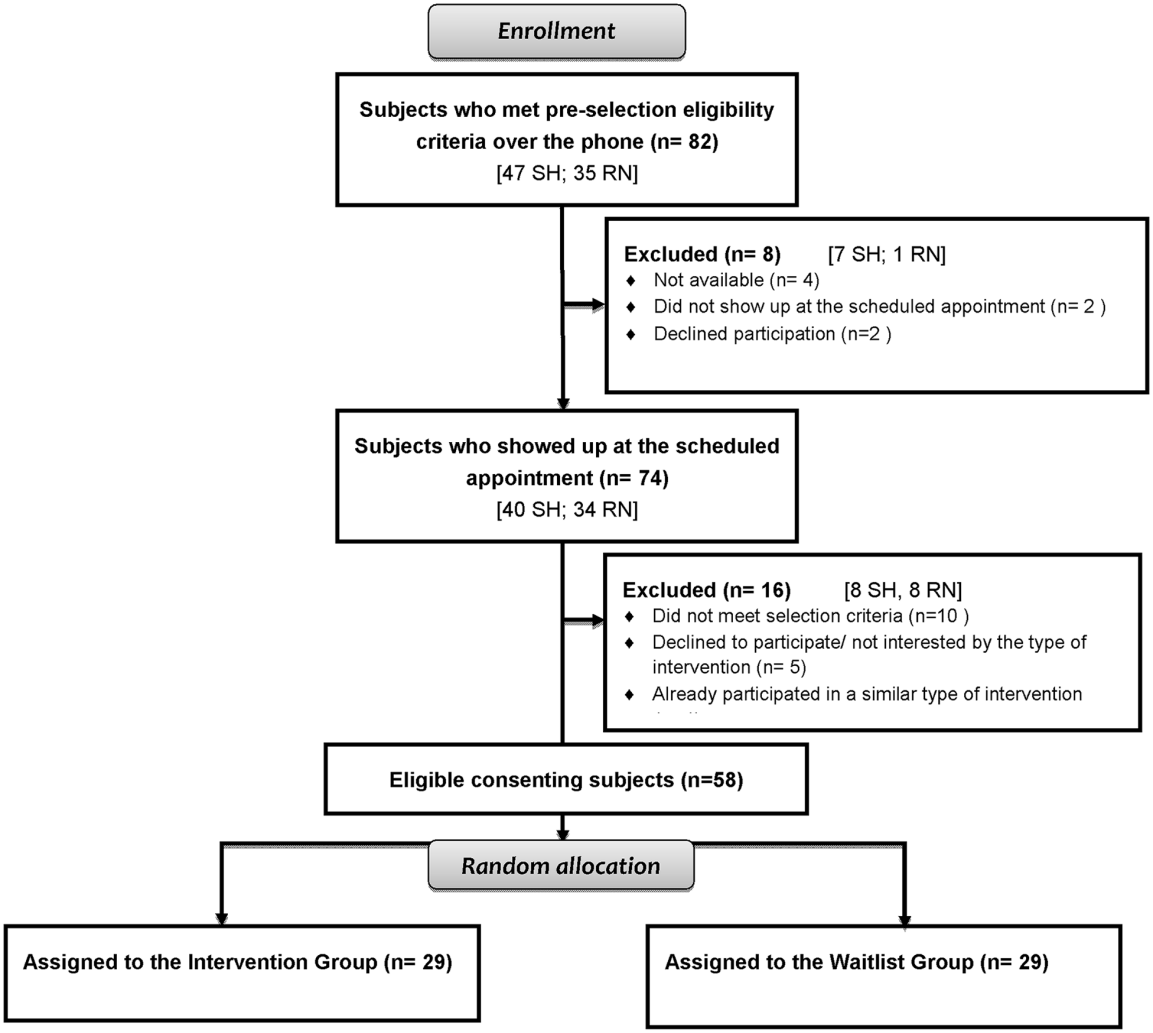
Flow of participants through the study at each assessment point. SH = Sherbrooke study site; RN = Rouyn-Noranda study site.

A mixed-methods, multicenter, open label, randomized, wait-list controlled trial with both a quantitative and a qualitative component was carried out in two university-affiliated settings between September 2009 and March 2011: 1) Sherbrooke, a suburban city located in the south of the province of Quebec (Canada), and 2) Rouyn-Noranda, a small city in the north of the province of Quebec (Canada). Study sites were chosen because of the clinical expertise of local teams with the ISF. Fig 1 describes the flow of participants through the study at each assessment point.

### Eligibility, Recruitment, and Randomization

Subjects were eligible for participation in the study if they: a) were aged 18 years or older, b) were able to read, understand, and complete questionnaires in French, c) had a medical diagnosis of FMS based on the American College of Rheumatology (ACR) classification criteria [23] for at least 6 months, d) reported FMS pain of at least moderate intensity (≥ 4/10) in the seven days prior to enrolment, the FMS pain being the chief complaint if the patient suffered from another chronic pain syndrome, e) were motivated to attend all group sessions and to integrate the proposed self-management strategies, and f) agreed to not introduce new pain medications or other new pain treatment modalities during the 11 weeks of the intervention. Exclusion criteria were the following: a) pregnant or lactating women, b) presence of an active cancer, uncontrolled metabolic disease and other major physical or psychiatric disorder that could compromise patient participation in the study, and d) outstanding litigation regarding patient’s claim for disability payments.

Recruitment was conducted through announcements in local newspapers in both study sites between September 2009 and October 2009. Interested subjects were invited to call the research coordinator who explained the study, reviewed some of the eligibility criteria, and fixed a first appointment with the potential participants one month prior to the beginning of the intervention. At the time of the first appointment, a pain physician established the FMS diagnosis using the ACR criteria [23], and a physical/psychological evaluation was carried out to ensure the subjects met all the eligibility criteria including proper motivation to partake in the intervention. Written informed consents were obtained from all participants who were then randomly assigned to the Intervention (INT) Group (PASSAGE Program) or the Waitlist (WL) Group. Randomization was stratified by study site and gender, and was done by an independent third party using the Random Allocation Software—Version 1.0.0 (Isfahan, Iran).

### Description of the Group Conditions

#### Intervention (INT) group

As mentioned earlier, the PASSAGE Program is a structured multicomponent interdisciplinary group intervention aimed at reducing FMS symptoms and maintaining optimal function through the use of self-management strategies and patient education. The intervention consists of 9 group sessions with 8 participants lasting 2.5 hours each. As shown in Table 1, each session involved 3 major components—1) psycho-educational tools, 2) CBT-related techniques, and 3) patient-tailored exercise activities. Self-management of the main symptoms of FMS including pain, fatigue, poor sleep quality, and mood fluctuations were targeted during the course of the sessions as well as issues relating to stress management. An additional session was devoted to the pharmacological and non-pharmacological treatments of FMS. The first 8 sessions were held over a period of 11 weeks while the 9^th^ final session was carried out 6 months later to review progress and gain maintenance. The first two sessions were partly devoted to the establishment of a contract with the patient where she/he: 1) fixed three personal outcome goals to be met by the end of the intervention program, 2) determined the minimally acceptable changes to be expected, and 3) agreed to participate in all group sessions and to devote time during the week to the tasks prescribed at the end of each session—i.e., about 45 minutes/day, 6 times/week. Patients were informed that they will be excluded from the program if they missed 2 sessions.

**Table 1:** Summary of the components and content of the PASSAGE Program.

Week	Session	PSYCHO-EDUCATIONNAL TOOLS*	CBT-RELATED TECHNIQUES	EXERCISE ACTIVITIES
1	1	Introduction: Introduce facilitators and group members; Overview of PASSAGE objectives and content of sessions; Introduce the contract principles.	Fixing realistic objectives: Assess capacity to manage FMS on a 0–10 scale; Discuss importance of setting up realistic objectives; Plan to fix 3 personal objectives (outcome goals) along with minimally acceptable changes to be expected^*a*^; Homework assignments: ^*a*^ to ^*d*^.	On site exercises: Abdominal breathing^*b*^; Pelvic tilt exercises; Ergonomic sit, lay down and lay up^*d*^.
2	2	FMS symptoms: Briefly present the pathophysiology of FMS; Describe the main FMS symptoms including pain, fatigue, poor sleep quality, and mood fluctuations; Describe the effects and impact of stress on FMS.	Introduction to self-management strategies: Review of the fixed personal objectives (n = 3); Discuss the impact of FMS symptoms on various aspects of daily living; Share personal efficient strategies to control symptoms^*a*^; Introduce new strategies to improve sleep quality^*b*^; Introduce cardiorespiratory training^§^; Homework assignment: ^*a*^ to ^*f*^ + patient’s signature of the contract with a significant person + diary completion re: accomplished tasks at home.	On site exercises: Exercises with pressure balls; Abdominal breathing^*c*^; Pelvic tilt exercises. Home exercises: Personalised exercises program ^*e*^ ^£^; Cardiorespiratory training^*f*^.
3	3	Exercise and physical activity as part of FMS management: Introduce anatomy and functions of muscles; Present types of exercises and tips for starting exercise program; Discuss the impact of physical activities and exercise on FMS symptoms; Discuss the relevance of a personalized exercise program.	Awareness of personal strengths and limits: Physical testing; Discuss problem of de-conditioning and fear/avoidance attitudes; Discuss importance of respecting self capacities; Demonstration of personalised exercise program by the participants; Homework assignment: ^*a*^ to ^*c*^ + identification of one novel self-management strategy + diary completion.	On site exercises: Abdominal breathing-sitting position; Cervical stabilization exercise; Pelvic tilt exercise—lay down position; Jaw relaxation exercise^*a*^. Home exercises: Personalised exercises program^*b*^ ^α^; Cardiorespiratory training^*c*^.
4	4	Psychological tools as part of FMS management: Present the impact of the person’s psychological state on FMS symptoms; Describe the role of positive vs. negative thoughts/appraisals about FMS symptoms; Discuss the rational of a patient-tailored psychological program.	Awareness of the patients’ power over their health condition: Discuss the notion of “choice” regarding FMS management: passive consumer vs. active partner in the treatment; Identify negative or maladaptive thoughts that may affect FMS symptoms; Share how changes in perceptions may affect psychological (and physical) well-being^*a*^; Introduce problem-solving strategies and cognitive coping strategies^*b*^; Discuss the role of relaxation techniques for managing FMS symptoms; Homework assignment: ^*a*^ to ^*e*^ + diary completion.	On site exercises: Abdominal breathing—sitting position; Pelvic tilt exercise—sitting position; Symmetry exercise; Relaxation technique practice—anti-relaxation^*c*^. Home exercises: Personalised exercises program^*d*^; Cardiorespiratory training^*e*^.
5	5	Energy and capacity management: Describe the physiologic signs when exceeding personal capacities/limits; Discuss the importance of proper balance between activity and relaxation periods, and its impact on FMS symptoms; Discuss the role of a healthy alimentation for maximizing energy.	Awareness of the impact of stress and its relation with management of energy and capacities: Identification of own limits; Discuss activity pacing and importance of engaging in pleasant and meaningful activities; Discuss and share strategies to adequately manage energy and capacities^*a*^; Introduce new strategies to cope with personal limits, and especially in the context of stressful situations^*b*^; Tasting new healthy food products; Home assignments: ^*a*^ to ^*e*^ + identification of one sign of stress + one strategy to cope with personal limits + diary completion.	On site exercises: Personalised exercises program (team of 2); Abdominal breathing—sitting position; Pelvic tilt exercise—up position; Relaxation technique practice—active^c^ ^ϕ^. Home exercises: Personalised exercises program^*d*^; Cardiorespiratory training^*e*^.
6		Integration week^Ψ^		
7	6	The vicious circle of chronic pain: Briefly present the pathophysiology of chronic pain; Describe the impact of chronic pain on various aspects of daily living including mood, family relationships, sexuality, etc.; Understand how the vicious circle of chronic pain can develop and persist.	Awareness of more adverse effects of FMS: Discuss strategies to deal with pain flare-ups and setbacks; Discuss more devastating effects of FMS-related pain: e.g., social isolation, major depression, suicide, etc; Share strategies to cope with these symptoms^*a*^; Introduce new strategies that may be helpful in these situations^*b*^; Home assignments: ^*a*^ to ^*e*^ + diary completion.	On site exercises: Personalised exercises program (team of 2); Abdominal breathing—up position; Pelvic tilt exercise—in mouvement; Activity pacing; Relaxation technique practice—passive^*c*^. Home exercises: Personalised exercises program^*d*^; Cardiorespiratory training^*e*^.
8		Integration week		
9	7	Pharmacological and non-pharmacological treatment of FMS: Discuss the myth of the “magic pill” or “magic treatment”; Describe the rational for using pharmacological and non-pharmacological treatments; Present the major types of pain medication and their side effects; Describe the “to-do” and “not-to-do” with pain medications; Discuss the role of complementary therapies for the management of pain and other FMS symptoms.	Awareness of own personal judgement about FMS treatment: Discuss the untended consequences healing may have on the person (“Is healing really what I want?); As the “expert” of his/her condition, encourage the person to discover the customized balance of pharmacological and non-pharmacological methods that best suit his/her condition in the context of a problem-solving approach^*a*^; Discuss the role of pain medication as a way to increase function and physical activity; Home assignments: ^*a*^ to ^*e*^ + self-management strategies + diary completion.	On site exercises: Personalised exercises program (team of 2); Abdominal breathing—up position; Pelvic tilt exercise—in mouvement; Relaxation technique practice-visualisation^*b*^. Home exercises: Personalised exercises program^*c*^; Cardiorespiratory training^*d*^; Activity pacing^*e*^.
10		Integration week		
11	8	Review and summary: Summarize the knowledge and self-management strategies acquired during the program.	Awareness of control gain over symptoms and how to maintain this control: Re-assessment of the capacity to manage FMS on a 0–10 scale (session 1); Review of the fixed personal objectives and comparison with expected changes; Physical testing; Discussion on the ways to maintain gains made through the program and to keep control over symptoms with acquired strategies; Review strategies to deal with pain flare-ups and setbacks; Graduation with certificate of achievement; Home assignments: ^*a*^ to ^*c*^.	On site exercises: Personalised exercises program (alone); Abdominal breathing—all positions; Pelvic tilt exercise—all positions; Relaxation technique practice-personal choice^*a*^. At home exercises: Personalised exercises program^*b*^; Cardiorespiratory training^c^.
		Integration months		
6 months later	9	Follow-up visit	Awareness of actual condition: Discuss evolution of the condition since the beginning of the program; Identification of efficient strategies^*a*^; Personalised exercises program adjustment; Home assignments: ^*a*^ to ^*d*^.	On site exercises: Personalised exercises program (alone); Relaxation technique practice^*b*^. Home exercises: Personalised exercises program^*c*^; Cardiorespiratory training^*d*^.

CBT = Cognitive Behavioral Therapy.

* Except for Session 1, all educational sessions start with a brief overview of the participants’ preceding week(s) (e.g, achievements).

^£^ At the time of the very first appointment with the participants, those assigned to the Intervention Group were provided with a brief personalised exercise program based on the results of their physical evaluation.

^α^ Starting on Session 3, patient-tailored exercise programs are put in place and are practiced at the beginning of each session. Participants are encouraged to carry out their exercise program 20 min per day, 6 times a week.

^§^ Participants are invited to pick up one cardiorespiratory activity of their choice (e.g., swimming, walking, etc) and practice it for 20 minutes, 3 times a week.

^ϕ^ Starting at Session 4, participants are introduced and trained in using different types of relaxation techniques. Participants choose the technique they prefer and are encouraged to do a relaxation session at home 3 times a week

^Ψ^ At Week 6, 8, and 10, there is no session. These break periods provide the participants time to practice/integrate newly acquired self-management strategies and consolidate the exercises/relaxation programs at home

The sessions were always conducted in a well-equipped exercise room with mattresses, pillows, exercise balls, mirrors, sound system and computer equipment for Power Point presentations. These sessions were interactive and led by two health care professionals who both acted as facilitators, one being mainly responsible for the psychological aspect of the intervention and the other for its physical aspect. Patients were viewed as the “experts” of their condition, and were given a role of active partner in the management of their FMS. Except for Session 1, the others always started with customized exercise routines (15 min), including correction of posture and movements when needed. Participants were then asked to discuss their experiences with the prescribed tasks of the preceding week (including the practice of new self-management strategies) (15 min). Then, the two facilitators started the education part of the session during which various topics related to FMS symptoms and their management were covered (see Table 1). Participants were strongly encouraged to share their own experience and the tools/strategies they used to manage their condition which fostered patients' empowerment as well as their active participation as experts of their condition. This portion of the session, which lasted about 60 min, was followed by a 15-min break during which both participants and facilitators had the opportunity to socialize. In the second portion of the sessions, the facilitators proposed new self-management strategies, specific exercises, and respiration techniques. A strong emphasis was placed on the rationale behind the proposed strategies/techniques. Participants were invited to practice them during a 30-min period. Starting on Week 4, the exercise program ended with a relaxation session during which different techniques were taught and practiced (15 min). Finally, participants were prescribed tasks to be done during the following week(s). At any time during the sessions, participants were allowed to move, lie down, or use pillows to alleviate pain, if needed.

In order to ensure uniformity and standardization of the intervention program in both study sites, all health care professionals acting as facilitators attended a one-day structured training session which was held in Rouyn-Noranda (Québec, Canada). The first part of the training was devoted to theory (e.g., rationale behind the intervention, key concepts, procedures to follow during each session, the “*to-do*” and “*not-to-do*”, etc), and the second part took the form of a practicum with presentations of scenarios, role playing, and video demonstrations. The comprehensive course manual used during the training and given to each facilitator also contained a detailed description of the content of the sessions along with an annotated paper copy of the slides to be used in each session. A second training session was held via videoconference to clarify some issues, answer questions, and review the procedures.

To ensure facilitators’ adherence to the intervention protocol, all sessions were recorded and monitored (using back-surface mirror) by a health care professional with experience with the ISF and who participated in the development of PASSAGE Program. Conference calls involving the researchers and study coordinators from each study site were also made on a weekly basis to review the procedure, discuss issues raised during the sessions, and ensure uniformity.

#### Waitlist (WL) group

Participants randomized to the WL Group were instructed to continue their treatment(s) as usual until they could take part in the PASSAGE Program—i.e., 3 months after the INT Group had completed the program. Changes in pharmacological or non-pharmacological treatments were allowed during this period in the WL Group (usual care).

### Procedure

#### Quantitative study

**Fig 2:**
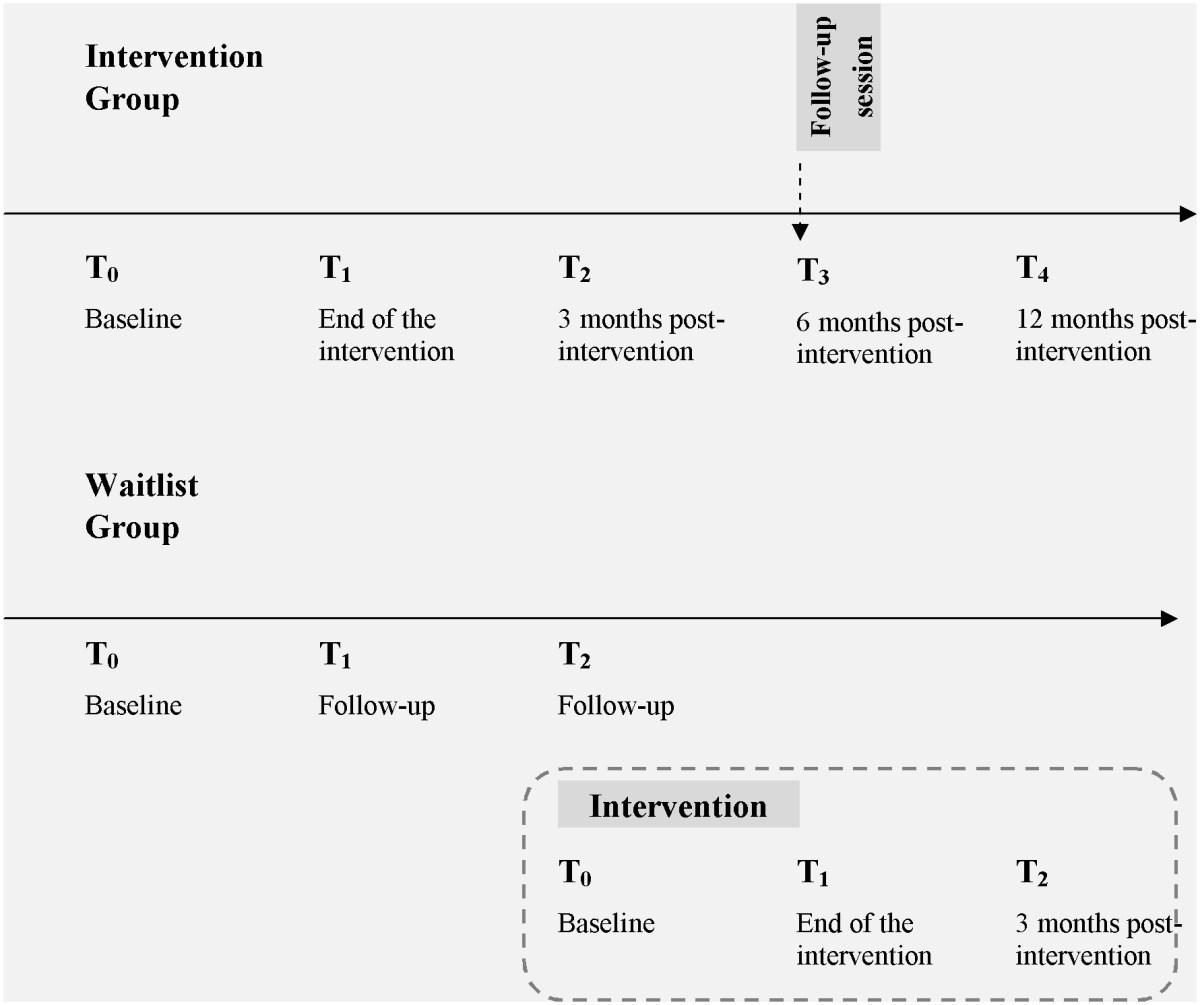
Timeline of data collection in each study group.

Data were collected in both study groups at baseline (T_0_), after the INT Group completed the 8 sessions of the PASSAGE Program (T_1_), and 3 months later (T_2_) (Fig 2). Patients of the WL Group were then offered the Program and completed follow-up measures at the end of the intervention (T_1_), and 3 months later (T_2_), thereby providing efficacy data from another cohort of patients. Additional follow-up measures were also collected in the INT Group at 6 (T_3_) and 12 (T_4_) months after the completion of the PASSAGE Program so longitudinal data (T_0_ to T_4_) were available to assess the long-term benefits of the intervention in this group.

Data were collected at each time point with a self-administered questionnaire which was mailed to the patients along with a stamped return envelope to be mailed back to the research team within the next 7 days. Reminder phone calls were made if the questionnaires were not returned on time. Upon reception, questionnaires were carefully verified, and a research assistant contacted the patients if any information was missing or if their depression scores on the BDI was > 30 and/or they reported suicidal ideas (question 9 of the Beck Depression Inventory) (see Section Protocol and Adjustments).

#### Qualitative study

In order to document and further capture the patients’ experiences, face-to-face open-ended narrative qualitative group interviews were conducted in each study site. Interviews took place 6 to 9 months after completion of the PASSAGE Program, and were conducted by the same interviewer in both sites. The interviewer had an extensive experience in qualitative research interviews and was, until then, unknown to the study participants. Nine patients from the Sherbrooke site (Québec, Canada) and 7 from the Rouyn-Noranda site (Québec, Canada) volunteered to participate in the group interviews. The same interview guide was used in both study sites and it included open-ended questions aimed at covering three main topics related to the research objectives. Participants were asked to talk about 1) their experiences during the intervention, 2) its impact on their daily life, and 3) their general appraisal of the intervention. The group interviews lasted between 60 and 90 minutes, and were audio-taped, entirely typed-written (*verbatim)*, and annotated with the interviewer’s field notes.

### Outcomes

#### Primary outcome

Pain intensity was the primary outcome and was measured with a standardized numerical rating scale (NRS) where 0 indicated “no pain” and 10 “worst possible pain” [21,24]. At each time point of the study, patients of both groups were asked to rate the average intensity of their pain as experienced in the past seven days.

#### Secondary outcomes

The choice of the secondary outcomes was based on the characteristics of the FMS symptomatology, the rational/objectives of the proposed intervention, and the IMMPACT Group recommendations [20,21] as well as the 2012 Canadian Guidelines for the Diagnosis and Management of FMS [7,8]. Two major sets of secondary outcomes, specific and global, were used to assess the effectiveness of the intervention. The selected measurement instruments are well-validated and widely used tools with documented psychometric qualities.

The first set of secondary outcomes measured specific symptoms or dimensions of the patients’ condition prior to the beginning of the intervention (T_0_) and at follow-up times—i.e., T_1_ and T_2_ in both groups, and T_3_ and T_4_ in the INT Group only.

Severity of FMS was measured with one of the most widely used tool in this research field, the Fibromyalgia Impact Questionnaire (FIQ) which is a disease-specific instrument designed to evaluate the impact of FMS by providing a multidimensional assessment of the overall severity of FMS [25,26]. The first 11 FIQ items ask about actual capacities regarding domestic activities and are answered to on a 4-point Likert scale ranging from 0 (always) to 3 (never). The last 9 FIQ items assess the presence and severity of various symptoms in the past seven days (pain, physical functioning, fatigue, morning tiredness, stiffness, depression, anxiety, job difficulty and overall well-being) using a numerical scale ranging from 0 (no symptoms) to 10 (major symptoms). The total FIQ score was calculated with a pre-determined algorithm (*www*.*myalgia*.*com/FIQ*) and ranges from 0 to 100, where a higher score indicates a greater impact of FMS.

The extent to which patients’ pain interfered with various aspects of their daily living was assessed with the 10 interference items of the Modified Brief Pain Inventory (BPI) [27,28]. These items include general activity, mood, walking ability, normal work, relations with others, sleep, enjoyment of life, personal care, recreational activities, and social activities in the past seven days. Items are rated on a 0 (does not interfere) to 10 (completely interferes) scale. The global BPI interference is derived by averaging the 10 items.

Considering the high frequency of sleep problems in FMS patients and the potential interrelations with pain [29,30], the Chronic Pain Sleep Inventory (CPSI) [31] was also administered to all participants to assess the impact of pain on sleep quality during the past 4 weeks. The CPSI is composed of 4 items answered to on a scale ranging from 0 (never) to 10 (always). Items are: 1) trouble falling asleep, 2) needing sleep medication, 3) awakening due to pain in the night, and 4) awakening due to pain in the morning. The fifth item of the CPSI assesses overall quality of sleep using a 0 (very poor) to 10 (excellent) scale. A total Sleep Problem Index Score (SPIS) is calculated by taking the sum of the scores on items 1, 3 and 4. The SPIS can range from 0 to 30 and higher scores indicate greater sleep problems.

The Coping Strategy Questionnaire (CSQ) [32,33] was used to assess the type of coping strategies participants employed day-to-day to cope with their pain. The CSQ includes 21 items answered to on a 4-point Likert scale ranging from 1 (never) to 4 (always). Items assess 5 coping strategies: 1) Ignoring pain sensations, 2) Diverting attention, 3) Catastrophizing, 4) Reinterpreting pain sensations, and 5) Praying. A score is obtained for each subscale by summing the scores on each of its items. In addition, patients’ tendency to catastrophize while they are in pain, which is known to have a profound impact on the experience of pain (see critical review [34]), was further investigated in the present study by administering the Pain Catastrophizing Scale (PCS) [35,36]. The PCS contains 13 items rated on a 5-point Likert scale ranging from 0 (not at all) to 4 (always). A total score is calculated by summing the score on each item.

The Beck Depression Inventory (BDI) Version 1 [37,38] was used to assess severity of depressive symptoms in the past seven days. This scale includes 21 items rated on a 4-point ordinal scale and a total score of the BDI (ranging from 0 to 63) can be obtained from the sum of all individual items. Higher scores indicate more severe depressive symptoms.

Health-related QOL was assessed with a generic instrument—the Standard SF-12v2 (4-week recall) [39]. This questionnaire covers 8 domains (i.e., Physical Functioning, Role-Physical, Bodily Pain, General Health, Vitality, Social Functioning, Role-Emotional, Mental Health) and the scores on each of these domains are summarized into 2 scales, the Physical Summary Scale and the Mental Summary scale. Scores on each summary scale were calculated with standard scoring algorithms and normalized using the US general population values (mean = 50; SD = 10).

Our second set of secondary outcomes was oriented towards patients’ global impression regarding changes in their condition and overall perception regarding their treatment responses in terms of pain relief. These measures were collected in both groups at T_1_ and T_2_, and at T_3_ and T_4_ in the INT Group only.

Participants were asked about their global impression of change in the past 3 months regarding their 1) pain, 2) level of functioning, and 3) QOL, using a modified version of the Patient Global Impression of Change (PGIC) Scale [40]. The scale ranged from 1 to 7 with “remained unchanged” as the mid-point, and “considerably deteriorated” and “considerably improved” as anchors. PGIC scores in each area were recoded into three categories: 1) Improved (slightly/ greatly/ considerably improved), 2) Stable (remained unchanged), and 3) Deteriorated (considerably/ greatly/ slightly/ deteriorated).

Patients’ overall perceptions of their treatment responses in terms of pain relief was assessed on a 0 to 100% Pain Relief Scale where 0% represents no pain relief and 100% represents complete pain relief [41][42]. Patients were asked to provide their ratings based on the preceding 3 months. Substantial improvement was defined with a cut-off point of pain relief ≥ 50% [43].

### Sample Size

The sample size was calculated for the primary outcome (NRS = average pain intensity over the past 7 days; continuous scale ranging from 0–10) based on testing the inequality of two means in a repeated measures design. Previous reviews [44–46] established that a 2-point reduction on the 0–10 NRS scale constitutes a clinically meaningful difference in pain intensity. Assuming the standard deviation of the NRS of 2.0 units and a study design with 3 repeated measurements having a compound symmetry covariance matrix, the sample size was determined based on the ability to detect, with a power of 80%, a change of 2 units or more in the average pain intensity score between the INT and WL groups at an two-sided alpha level of 0.05. Under these assumptions and conservatively assuming an autocorrelation coefficient (rho) of one, a group sample size of 16 patients, representing a total sample size of 32 patients was required. Given that the study was carried out in two sites and that each site was expected to have two groups, the sample size was doubled, and 64 patients in total were targeted (32 per study site). This strategy did not only increase our statistical power but also prevented reduced power because of patient loss to follow-up. Sample size estimation was performed using PASS 2008 and all statistical analyses were performed using SAS Version 9.2 (SAS Institute, NC, USA). The investigator in charge of the statistical analyses (A.L.) was blinded to group assignment.

### Quantitative Data Analysis

Comparisons of the baseline (T_0_) sociodemographic and pain characteristics between the INT and WL Groups were carried out with parametric and non-parametric tests depending on the type of variables and their distributions (i.e., t-test, Wilcoxon rank-sum test, Chi-square test, Fisher’s exact test). To detect if significant differences in continuous outcomes over time (T_0_, T_1_, and T_2_) were due to the intervention (*Group* x *Time* interaction effect), linear mixed models for repeated measures were used: 1) unadjusted model, 2) gender and study site adjusted model, and 3) fully adjusted model (study site, gender, living arrangements, work status, pain duration and use of pain medication). When interaction effects were detected, post hoc pairwise comparisons using t-tests or Wilcoxon rank-sum tests were carried out with a Bonferroni correction. Effect sizes are presented as raw group differences (mean differences) and their 95% confidence intervals (95% CI). A negative effect size value indicates that the intervention was superior to the control group on negatively oriented outcome measures (i.e. pain intensity NRS, FIQ score, BPI interference score, CPSI sleep problem index score, catastrophizing CSQ score, PCS score, BDI score). A positive value indicates the intervention was superior to the control group on positively oriented outcome measures (i.e. CPSI overall sleep quality score, other CSQ subscales scores, SF-12v2 health-related QOL scores).

Categorical outcomes such as the PGIC (proportion of patients reporting improvement) and pain relief (proportion of patients reporting ≥ 50% of pain relief) in the past 3 months were compared between the two study groups at the end of the intervention (T_1_) and 3 months post-intervention (T_2_) using Chi-square tests and Fisher exact tests where appropriate. Effect sizes were computed as odds ratios (OR) and their 95% CI.

As previously mentioned, additional data were collected from the WL Group at the end of the trial once they had the opportunity to participate in the PASSAGE Program. These data were used to conduct sensitivity analyses to see if the pattern of results observed on the global outcome measures at T_1_ and T_2_ were similar to the one observed in the INT Group.

With the 12 months follow-up data from the INT Group, additional analyses were carried out to assess the effect of the treatment over a longer period of time. One-way ANOVAs with repeated measures on one factor (within-subjects time effect between T_0_, T_1_, T_2_, T_3_ and T_4_) were conducted. When significant differences were detected, post hoc comparisons were carried out using t-tests or Wilcoxon signed rank sum test. Raw effect sizes between T_0_ and T_4_ measures are presented as mean differences and their respective 95% CI.

### Qualitative Data Analysis

A thematic analysis of the qualitative data was conducted using the methodology proposed by Mucchielli [47]. All *verbatim* were reviewed line by line, summarized in words, and transformed into codes. MS Excel software was used to create a coding tree, and codes were combined to identify emerging themes which were then classified into main themes and associated themes. The analysis was done independently by two investigators (P.B., R.C-H) who then compared and reviewed their results until a consensus was reached.

## Results

### Participants’ Recruitment

As shown in Fig 1, 24 subjects were excluded throughout the study selection process, leaving a total of 58 eligible patients who were randomly assigned to the INT Group (n = 29) and the WL Group (n = 29). Fifteen patients (31.0% in the INT Group (9/29) vs. 20.7% (6/29) in the WL Group) did not complete the 3-month trial: two out of fifty-eight were excluded from the program because of non compliance and the others withdrew either because they: 1) were no more able to attend the sessions due to a scheduling conflict (n = 3/58)), 2) developed a medical disorder unrelated to FMS (n = 3/58), 3) went through an episode of psychological instability (n = 2/58), or 4) for personal reasons (n = 1/58). Four out of fifty-eight participants failed to return their study questionnaires by mail at one time or another, and did not provided complete longitudinal data. Consequently, a total of 43 patients completed the T_2_ measures: 20/43 received the intervention and 23/43 were on the waitlist. As mentioned earlier, this last group received the intervention at the end of the trial and were assessed up to 3 months post-intervention (17/23 patients completed follow-up).

### Participants’ Characteristics

Socio-demographic and pain characteristics of the randomly assigned participants are presented in Table 2. Their mean age was 49.98 ± 9.23 years and 46.74 ± 11.42 years in the INT and WL Groups, respectively. As expected [4,6], there was a greater proportion of women in both study groups (> 92%, 26/28 in the INT Group and 26/28 in WL Group). More than half of the subjects (18/28 in the INT Group and 15/28 in the WL Group) had completed a university education level. The mean duration of pain was > 10 years in both groups (INT Group: 15.66 ± 11.12 years, WL Group: 11.94 ± 8.23 years) and the pain intensity levels on the NRS (average pain in the past 7 days) were comparable (INT Group: 6.57 ± 2.03, WL Group: 6.39 ± 1.83). The only statistically significant difference between the groups was the proportion of patients who were using prescribed pain medication; this proportion was lower in the INT Group (78.57%, 22/28) than it was in the WL Group (100%, 28/28).

**Table 2:** Characteristics of the participants who completed the baseline evaluation.

	INT Group (n = 28)		WL Group (n = 28)	
**Sociodemographics**				
Age	49.98	(9.23)	46.74	(11.42)
Sex (Females)	26	(92.9%)	26	(92.9%)
Ethnicity (Caucasians)	28	(100%)	27	(96.4%)
Education level				
Collegial/University not completed	10	(35.7%)	13	(46.4%)
University completed	18	(64.3%)	15	(53.5%)
Living arrangement				
Living alone	5	(17.9%)	6	(21.4%)
With spouse/partner	22	(78.6%)	19	(67.9%)
Other living arrangements *	1	(3.6%)	3	(10.7%)
Work status				
Full-time job	6	(21.4%)	5	(17.9%)
Part-time job	6	(21.4%)	8	(28.6%)
Medical disability	7	(25.0%)	12	(42.9%)
Not working †	9	(32.1%)	3	(10.7%)
Household income (cdn$/year)				
Less than 20000$	6	(22.2%)	5	(17.9%)
Between 20000 and 49999$	11	(40.7%)	10	(35.7%)
Between 50000 and 79999$	8	(29.6%)	10	(35.7%)
80000$ and over	2	(7.4%)	3	(10.7%)
**Pain characteristics**				
Pain duration (yr)	15.66	(11.12)	11.94	(8.23)
Average pain intensity in the past 7 days (NRS)	6.57	(2.03)	6.39	(1.83)
**Use of pain-related medications & natural products**				
Use of OTC pain-related medication	20	(71.4%)	24	(85.7%)
Use of prescribed pain medication	22	(78.6%)	28	(100%)
Use of pain-related natural products	15	(53.6%)	17	(60.7%)

Data are presented as mean ± standard deviation values, or number of patients and percentage.

INT Group: Intervention Group

WL Group: Waitlist Group

NRS = Numerical rating scale

OTC = Over-the-counter

*Living with children (n = 1), parents (n = 3), or brothers/sisters (n = 1)

^†^Retired, students, volunteer work

### Efficacy of the PASSAGE Program up to 3 Months Post-Intervention

#### Primary outcome

As shown in Table 3, average pain intensity scores were comparable between the two study groups from baseline up to 3 months post-intervention. No significant *Group* x *Time* interaction was found (*P* > .05). Effect sizes of -0.13 (95% CI: -1.37 to 1.11) and -0.55 (95% CI: -1.82 to 0.72) were found between study groups at T1 and T2 respectively.

**Table 3:** Magnitude and significance of the improvements in the intervention (INT, n = 28) and the waitlist (WL, n = 29) groups up to three months post-intervention.

	Baseline (T_0_)				End of the intervention (T_1_)					3 months post-intervention (T_2_)					Repeated measures analyses p-values**
Study outcomes*	INT Group		WL Group		INT Group		WL Group		Effect sizeand 95% CI	INT Group		WL Group		Effect size and 95% CI	*Group* x *Time* effect
**PRIMARY OUTCOME**															
**Pain intensity**															
Pain on the average in the past 7 days (NRS: 0–10)	6.57	± 2.03	6.39	± 1.83	5.95	± 2.06	6.08	± 2.14	-0.13 (-1.37–1.11)	5.36	± 1.74	5.91	± 2.29	-0.55 (-1.82–0.72)	0.669, 0.701, 0.778
**SECONDARY OUTCOMES**															
**Severity of FMS**															
Total FIQ score (0–100)	64.68	± 16.80	62.43	± 18.90	55.11	± 16.22	56.68	± 20.66	-1.57 (-12.59–9.45)	51.49	± 16.27	55.18	± 20.44	-3.69 (-15.19–7.18)	0.465, 0.537, 0.665
**Pain interference**															
BPI interference mean score (0–10)	5.09	± 2.38	5.36	± 2.40	4.63	± 2.15	4.99	± 2.32	-0.36 (-1.68–0.96)	4.08	± 2.14	4.72	± 2.24	-0.64 (-1.99–0.71)	0.954, 0.958, 0.988
**Sleep quality**															
CPSI—Overall sleep quality item (0–10)	2.75	± 1.82	2.89	± 2.59	4.09	± 2.04	3.72	± 2.30	0.37 (-0.92–1.66)	4.33	± 2.18	3.57	± 2.37	0.76 (-0.65–2.17)	0.512, 0.578, 0.691
CPSI—Sleep Problem Index score (0–30)	18.00	± 8.49	18.89	± 7.91	12.50	± 7.41	15.12	± 7.72	-2.62 (-7.08–1.84)	14.19	± 8.60	16.65	± 8.00	-2.46 (-7.57–2.65)	0.711, 0.732, 0.877
**Coping Strategy Questionnaire**															
Ignoring Pain Sensations Subscale (0–15)	7.14	± 3.14	5.93	± 2.61	6.41	± 2.28	6.52	± 2.60	-0.11 (-1.56–1.34)	8.10	± 3.13	6.00	± 2.65	2.10 (0.32–3.88)	0.010, 0.014, 0.052
Diverting Attention Subscale (0–15)	7.39	± 3.82	7.96	± 2.59	7.77	± 2.65	8.36	± 2.06	-0.59 (-1.98–0.80)	7.71	± 3.21	7.09	± 2.37	0.62 (-1.10–2.34)	0.121, 0.118, 0.219
Catastrophizing Subscale (0–12)	4.50	± 3.00	4.93	± 3.05	3.86	± 2.61	3.64	± 3.13	0.22 (-1.49–1.93)	3.38	± 2.91	4.13	± 2.88	-0.75 (-2.54–1.04)	0.496, 0.532, 0.724
Reinterpreting Pain Sensations Subscale (0–12)	3.39	± 3.06	2.68	± 2.26	3.36	± 3.49	2.76	± 2.17	0.60 (-1.09–2.29)	4.10	± 3.75	2.70	± 2.85	1.40 (-0.64–3.44)	0.961, 0.972, 0.969
Praying Subscale (0–9)	2.11	± 2.04	3.11	± 2.67	2.09	± 2.27	2.92	± 2.78	-0.83 (-2.33–0.67)	2.05	± 2.42	2.78	± 2.98	-0.73 (-2.42–0.96)	0.789, 0.785, 0.820
**Pain catastrophizing**															
Total PCS score (0–52)	23.54	± 11.10	22.04	± 11.98	17.86	± 9.83	18.88	± 12.75	-1.02 (-7.78–5.74)	15.62	± 13.43	20.00	± 11.23	-4.38 (-11.97–3.21)	0.193, 0.234, 0.364
**Depression levels**															
BDI total score (0–63)	19.54	± 9.39	18.61	± 9.37	16.91	± 7.84	16.56	± 10.39	0.35 (-5.12–5.82)	16.05	± 7.73	16.78	± 10.00	-0.73 (-6.30–4.84)	0.870, 0.868, 0.857
**Health-related quality of life**															
Physical Summary Scale of the SF-12v2 (0–100)	31.21	± 8.95	29.59	± 10.46	30.55	± 8.17	29.41	± 11.08	1.14 (-4.65–6.93)	30.49	± 7.90	28.65	± 9.09	1.84 (-3.44–7.12)	0.962, 0.950, 0.930
Mental Summary Scale of the SF-12v2 (0–100)	40.58	± 11.39	40.94	± 9.00	40.74	± 8.42	39.07	± 11.28	1.67 (-4.25–7.59)	40.75	± 10.49	37.59	± 9.76	3.16 (-3.08–9.40)	0.444, 0.450, 0.505

* Data are presented as mean ± standard deviation values.

** P-values are presented for the *Group x Time* effect and show whether the mean scores across time depended or not upon the intervention. The first line presents unadjusted mixed model p-values while the second line are the p-values for gender and study site adjusted model and the fully adjusted model (study site, gender, living arrangements, work status, pain duration and use of pain medication). All models are Kenward-Roger adjusted.

BPI = Brief Pain Inventory; higher scores indicate more pain interference with various aspects of daily living; BDI = Beck Depression Inventory; higher scores indicate more severe depressive symptoms; CPSI = Chronic Pain Sleep Inventory; Overall Sleep Quality item: higher scores indicate better sleep quality; Sleep Problem Index score: higher scores indicate greater problems; CSQ = Coping Strategy Questionnaire: higher scores on ignoring pain sensations, diverting attention, reinterpreting pain sensations, and praying subscales indicate greater use of the coping strategy; higher scores on the catastrophizing subscale indicate a greater tendency to catastrophize; FIQ = Fibromyalgia Impact Questionnaire: higher scores indicate greater FMS severity; PCS = Pain Catastrophizing Scale: higher scores indicate a greater tendency to catastrophize in the face of pain;

SF-12v2 = 12-Item Short Form Health Survey version 2: higher scores indicate better health-related quality of life.

#### Specific secondary outcomes

Table 3 shows the variations in the mean scores (± SD), effect sizes and their 95% CI on the continuous secondary outcome (specific outcome measures) obtained in the INT and WL Groups at T_0_, T_1_, and T_2_. The mixed models results revealed no significant interaction between time and group assignment on the majority of these outcome measures. In other words, when compared with the WL Group, patients in the INT Group did not show more or less improvements over time with regard to the severity of their FMS condition (Fibromyalgia Impact Questionnaire), the extent to which their pain interfered with their daily living, the quality of their sleep, the type of strategies they used to cope with their pain, their tendency to catastrophize in the face of pain, the physical component of their health-related QOL (Physical Summary Scale of the SF-12v2), and their psychological well-being (Depression BDI Scores, Mental Summary Scale of the SF-12v2). The only *Time* x *Group* interaction effect that reached statistical significance was on the Ignoring Pain Sensations Subscale (*P* = 0.010) of the Coping Strategy Questionnaire. Although these results were suggestive of positive impact of the intervention on this measure, the results of the post-hoc analyses were not statistically significant after application of the Bonferronni correction.

#### Global secondary outcomes

**Fig 3:**
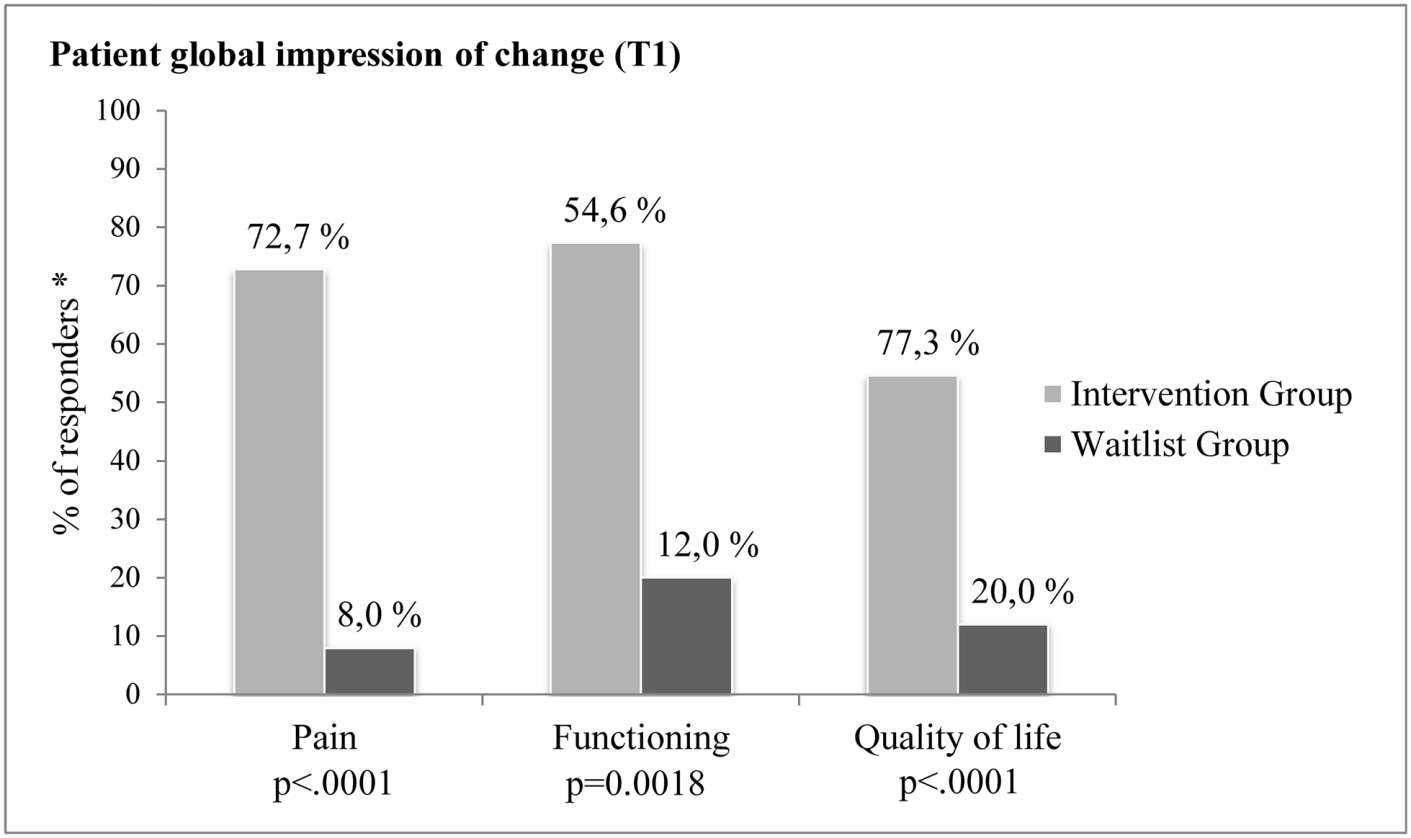
Percentage of patients in the Intervention and Waitlist Groups who reported that their pain, functioning and quality of life improved (slightly, greatly, or considerably) at T1 compared to baseline (T0) on the Patient Global Impression of Change Scales.

**Fig 4:**
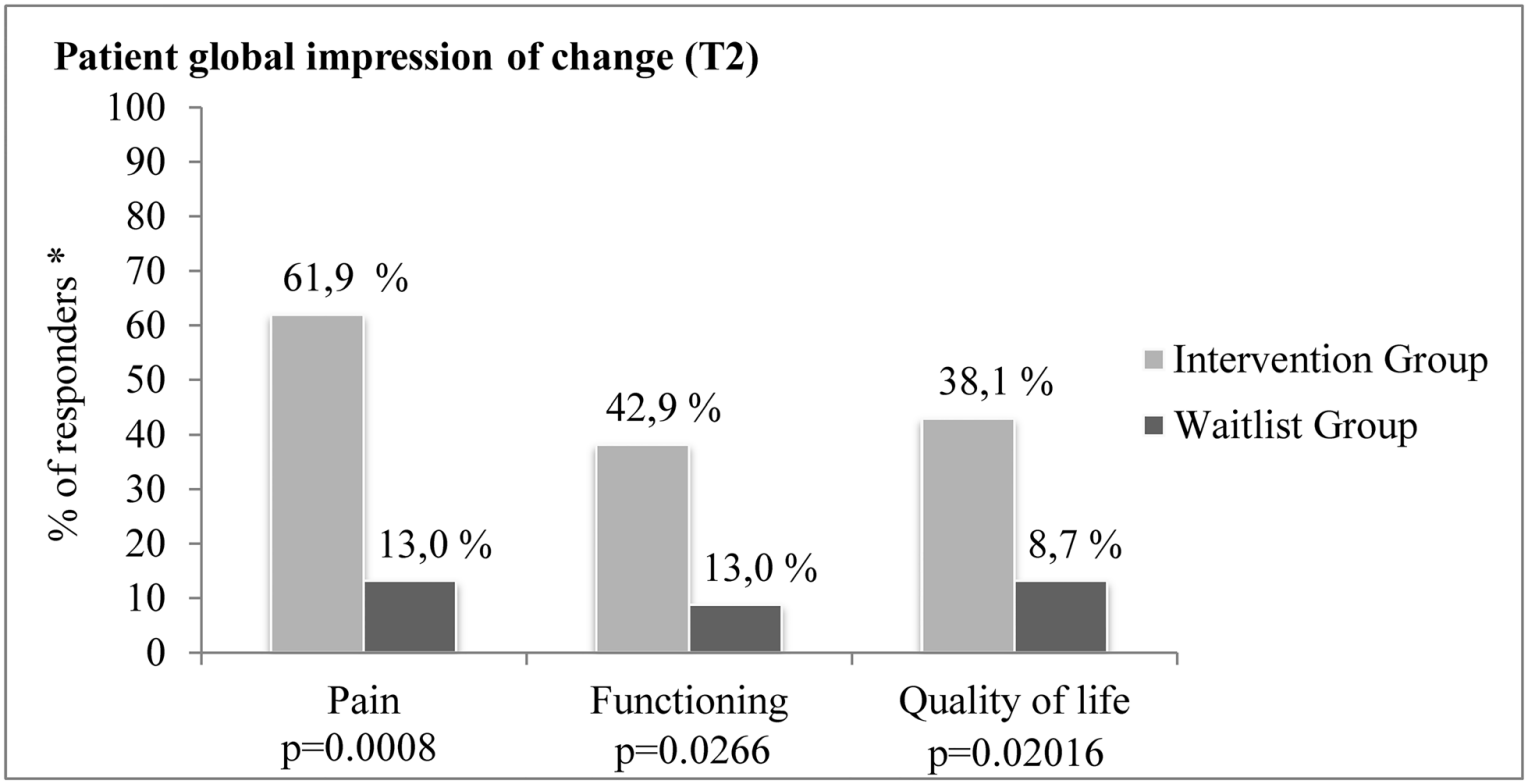
Percentage of patients in the Intervention Group who reported that their condition (pain, functioning and quality of life) continued to improve or remained stable 3 months after the intervention (T2) compared to the Waitlist Group on the Patient Global Impression of Change Scales.

A different pattern of results emerged in the second set of secondary outcomes—i.e., measures of patients’ global impression of change (PGIC) regarding changes in their condition and overall perceived pain relief. Comparisons of the PGIC ratings revealed statistically significant differences between the two study groups at T_1_ (end of intervention). Specifically, the likelihoods of reporting overall improvement in pain (OR: 30.67; 95% CI: 5.48 to 171.73; *P* < .0001), level of functioning (OR: 8.80; 95% CI: 2.02 to 38.25; *P* = .002), and QOL (OR: 13.60; 95% CI: 3.36 to 55.04; *P* < .0001) were higher in the INT Group than in the WL one. As shown in Fig 3, 72.7% (16/22), 54.6% (12/22), and 77.3% (17/22) of the patients of the INT Group reported improvements on these indicators between T_0_ and T_1_ compared with 8.0% (2/25), 12.0% (3/25) and 20.0% (5/25) in the WL Group. Furthermore, patients in the INT Group continued to perceive significant improvements in the 3 months following the intervention (i.e., between T_1_ and T_2_). When compared to the patients of the WL group, those of the INT Group were significantly more likely to report improvement in pain (OR: 10.83; 95% CI: 2.42 to 48.52; *P* = .0008), level of functioning (OR: 5.00; 95% CI: 1.13 to 22.18; *P* = .0266), and QOL (OR: 6.46; 95% CI: 1.184 to 35.26; *P* = .0201). As for these three outcomes respectively, 61.9% (13/21), 42.9% (9/21), and 38.1% (8/21) of the patients in the INT Group continued to perceive improvements compared with 13.0% (3/23), 13.0% (3/23) and 8.7% (2/23) in the WL Group (Fig 4) (one subject in the INT Group returned the questionnaire at T_2_ but not at T_1_). As previously mentioned, additional PGIC ratings were collected in the WL Group (17/23) at the end of the trial once they also completed the PASSAGE Program. Close to 60% of them reported improvements in their pain (58.8%; 10/17), functioning (58.8%; 10/17), and QOL (58.8%; 10/17) between T_0_ and T_1_, while the percentage of those who continued to improve on these indicators 3 months following the intervention was 35.29% (6/17), 23.53% (4/17), and 29.41% (5/17) respectively.

Significant group differences were also found in the measure of patients’ overall perceptions of pain relief. The proportion of patients reporting ≥ 50% pain relief between T_0_ and T_1_ was significantly higher in the INT Group (36.4%; 8/22) than in the WL Group (12.0%; 3/25) (OR: 4.19; 95% CI: 0.95 to 18.53; *P* = .049). Three months later (T_2_), one third (33.3%; 7/21) of the patients in the INT Group reported ≥ 50% pain relief compared to only 4.3% (1/23) in the WL Group (OR: 11.00; 95% CI: 1.22 to 99.25; *P* = .013). Once the WL Group completed the PASSAGE Program, 23.5% (4/17) of them reported ≥ 50% pain relief, and this percentage remained the same 3 months later.

### Efficacy of the PASSAGE Program up to 12 Months Post-Intervention

**Fig 5:**
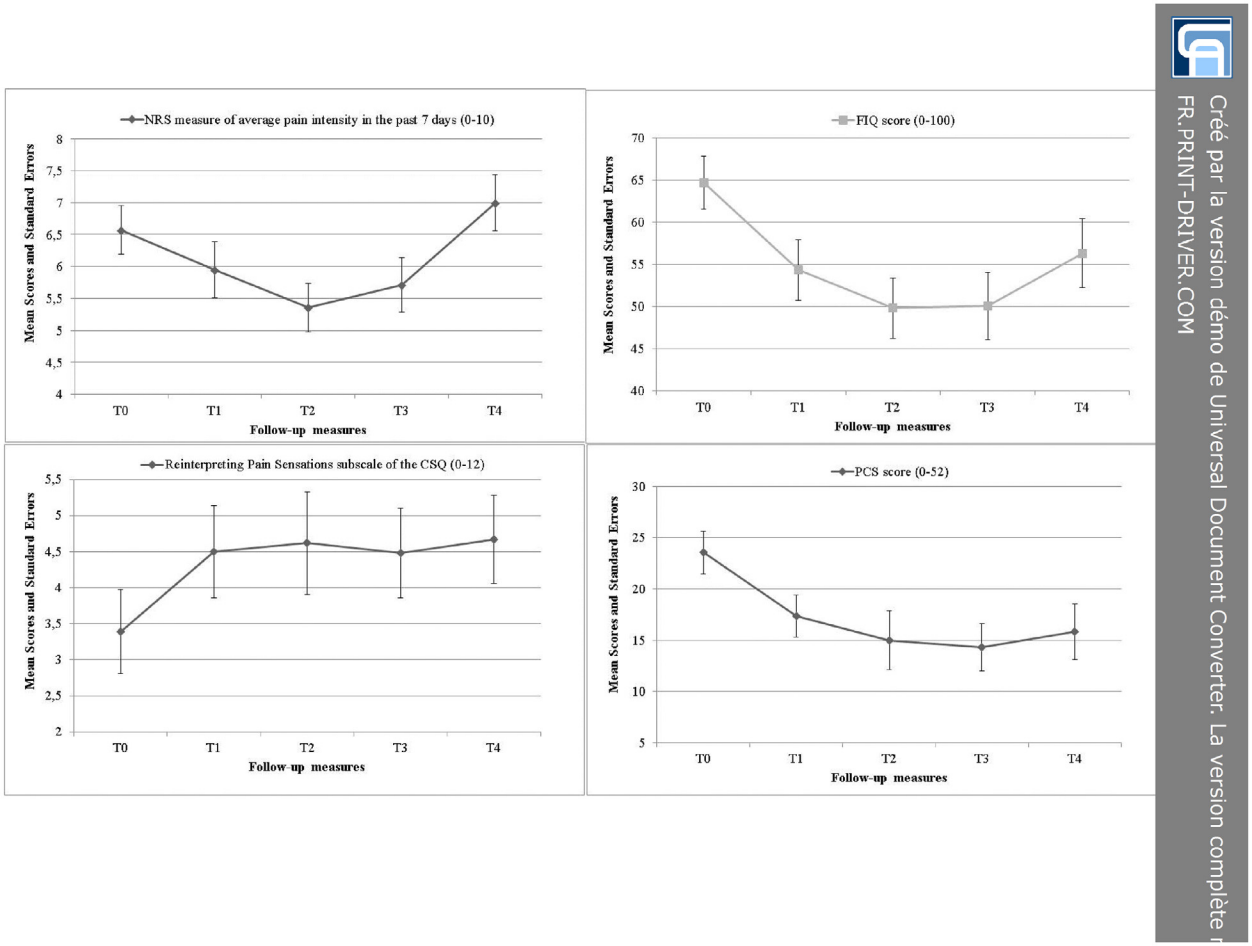
Mean scores and their Standard Errors in the Intervention Group at baseline (T0), at the end of the intervention (T1), and at 3, 6, and 12 months thereafter (T2, T3, T4). NRS = Numerical Rating Scale (higher scores indicate greater pain intensity); FIQ = Fibromyalgia Impact Questionnaire (higher scores indicate greater FMS severity); CSQ = Coping Strategies Questionnaire (higher scores indicate greater use of the strategy/coping efforts); PCS = Pain Catastrophizing Scale (higher scores reflect greater pain catastrophizing).

A total of 18 patients (64.5%) assigned to the INT Group completed additional follow- up measures 6 (T_3_) and 12 months (T_4_) after the completion of the PASSAGE Program. One-way ANOVAs with repeated measures on one factor (time) revealed statistically significant mean differences across the different follow-up times (T_0_ to T_4_) regarding the NRS measure of average pain intensity in the past 7 days (*P* = .0263), the Fibromyalgia Impact Questionnaire (*P* = .0041), the Reinterpreting Pain Sensations subscale of the Coping Strategy Questionnaire (*P* = .0071), and the Pain Catastrophizing Scale (*P* = .0007) (Fig 5). Although these results suggest that some clinical benefits of the intervention were maintained on the long-term on certain specific outcome measures, the results of the post-hoc analyses revealed statistically significant differences between the baseline scores (T_0_) and those at 12 months post-intervention (T_4_) only for the Reinterpreting Pain Sensations subscale of the Coping Strategy Questionnaire (Effect size: 1.83; CI:0.79 to 2.87) and the Pain Catastrophizing Scale (Effect size: -6.89; CI: -10.38 to -3.39).

On the global outcome measures, a good proportion of patients continued to report improvements or remained stable as revealed by their scores on the PGIC scales (pain, functioning, and QOL) at 6 (T_3_) and 12 months post-intervention (T_4_). In fact, at T_3_, 33.3 to 47.6% of the patients still reported improvements as experienced in the preceding 3 months while 23.8 to 38.1% reported being stable. Twelve months post-intervention (T_4_), 11.1 to 16.7% of patients reported improvements as experienced in the preceding 3 months while 16.7 to 38.0% reported stable levels. When asked about the percentage of pain relief experienced in the past 3 months, more than one quarter (28.6%) of the participants reported ≥ 50% pain relief at follow-up 6 months (T_3_) while 5.6% did so at follow-up 12 months (T_4_).

### Qualitative Results

Results of the thematic analysis of *verbatim* highlighted three major themes. The first one was UNCONDITIONAL ACCEPTANCE. All participants reported having greatly appreciated the intervention. Many of their comments were directed towards the facilitators—i.e., their unconditional acceptance, understanding, open-mindedness, kindness, availability and warm approach. For instance, some participants stated “*We felt understood; they know the disease*”, “*Just a look*, *and they knew what was wrong*” and “*I felt I was really important during the meetings*”. The majority of the participants stressed the difference between the PASSAGE Program and the care they usually receive where they do not feel supported by their healthcare providers: “*Our doctors don’t believe in our pain*, *we don’t know where to go and to whom to talk about FMS*”. All interviewed participants said they would partake in the intervention again and would recommend it to other FMS patients.

The second emerging theme was GROUP cohesion. It appeared that being part of a group was more important than the participants expected it would be specifically in terms of the opportunity for: 1) sharing “*She may have a trick that you didn’t think of*. *It helps to find new strategies*”; 2) support “*I didn’t know that so many people suffered from FMS*, *now I feel less lonely*”; and 3) motivation “*Motivation of others helped maintain my own motivation*.”; “*The effect of the group stayed even when I was home*. *That is why I exercised even when I wasn’t in a mood to do so*.” and “*Even if I didn’t feel like going to the meeting*, *I went because I knew I would feel better*.”

The last emerging theme was INCREASED EMPOWERMENT. Participants described that the impact of the intervention went beyond the symptoms themselves in that they acquired new knowledge and learned how to self-manage their condition. “*They gave me tools to gain control over my symptoms and to understand how to do it*.”; “*They helped me realize that I did too much exercising*. *I learned to manage my energy*.“; and “*I learned how to say no and to accept my limits*.”. The intervention also brought some behavioural changes among the participants: “*Now when I talk to my friends*, *I am not talking only about my disease*. *I ask them how they are feeling*.” and “*I now have leisure activities*, *I go out with friends*.”.

Finally, the following verbatim provides a meaningful and insightful illustration of the global impact of the intervention: “*At the beginning of the intervention*, *I realized that my pain was like a budget*. *I understood that I will always have the same amount of money but I will now manage it differently*. *This is really different*. *When you manage your pain*, *it is less present*, *less intense*”.

## Discussion

The results of the present study suggest that the PASSAGE intervention had a positive short-term impact on patients’ overall perceptions of their condition as revealed by their scores on the PGIC (pain, functioning, QOL) and pain relief scales compared to the patients who were assigned to the waitlist during this same period. Additional follow-up measures in the INT Group also showed that patients continued to improve or remained stable at 6- and 12-months post-intervention. For instance, at 6 months post intervention, between 57% and 85% of the INT participants were either stable or still noticing improvements on the PGIC outcomes and the pain relief measure. Results of the qualitative component of the study further supported the quantitative findings by showing that the intervention was effective in helping FMS patients gain an impression of control over their symptoms. However, no improvements were found on the primary outcome (NRS pain intensity) and the secondary specific outcome measures. The present study has a number of implications which are discussed below.

### Short-Term Impact of the Intervention

The hypothesis of the present study was that a multicomponent interdisciplinary self-management intervention would lead to a reduction in pain intensity and a lower impact of pain and FMS symptoms on various aspects of daily living including sleep quality and emotional well-being (depression), improved health-related QOL, less tendency to catastrophize in face of pain, and better pain coping strategies. However, no significant differences on these measures were found between the INT and the WL Groups. Nevertheless, significant improvements were observed on patients' global impression of change in pain symptoms, functioning, and QOL, as well as improvements in their perceived pain relief. It is thus possible that a measure assessing patients' global impression of change may be more reflective of the impact of an intervention like the PASSAGE Program than the primary outcomes generally used.

Numeric scales, like the 0–10 pain intensity scale, are often considered as the primary outcome in pain treatments clinical trials [21], although these measures are more and more criticized [43]. In the present study, no significant changes were found on the 0–10 pain intensity numeric scale which further suggests that this measure may not be the ideal primary outcome when it comes to the evaluation of an intervention designed for the FMS population.

Similar conclusions have being reached by other researchers as PGICs are increasingly being used as a gold standard in chronic pain treatments clinical trials [44,48]. For instance, improvements as measured by PGIC alone can be recognized as a response to a pharmaceutical treatment [48]. In pharmacological trials among FMS patients, correlations have been reported between PGIC and clinical pain, physical functioning, fatigue and impact on daily living [49]. Furthermore, because of the multidimensional nature of pain, QOL, improvements in functioning are increasingly used as markers of clinical significance when evaluating the efficacy of treatments [43]. A meta-analysis of the efficacy of pain medications such as gabapentin and pregabalin concluded that, even with a moderate pain reduction, QOL could be very much improved in chronic pain patients [50] and FMS patients [51]. Thus, greater attention should be given to the selection of primary outcomes when studying the FMS population with a specific concern for assessing patient global impression of change and overall perceived pain relief.

### Teaching Self-Management of Symptoms

The results of the qualitative and quantitative components of this study further suggest that the PASSAGE Program was highly successful in teaching patients to self-manage their illness and to take control over their pain management. One fascinating analogy emerged from the qualitative interviews where one participant described the management of his pain as one would describe the management of a budget. This participant mentioned that even though she will always have the same amount of money (—i.e., pain) what really matters is how she manages it. The better you manage your money (—i.e., pain), the less poor you feel (—i.e, the less intense and present the pain feels). The empowerment described by this patient and many others shows that the PASSAGE Program was successful in helping patients improve the self-management of their pain through the learning and implementation of new strategies. Furthermore, the quantitative results regarding the improvements of coping strategies and the increased ability to ignore pain sensations further demonstrate that the program was successful in this regard.

Elements of the PASSAGE Program such as patient education and aerobic exercise could have had a positive influence as they are known to increase global well-being and physical functioning as well as to decrease pain (e.g., [52–54]. For instance, Hävermark and Langius-Eklöf [55] evaluated the impact of a physical therapy-based educational program (including information, exercise and relaxation) with groups of 15 FMS patients seen twice weekly for 2 hours over a period of 10 weeks, and found a positive short-term effect on symptoms as well as a long-term effect on well-being.

However, it is important to note that the PASSAGE Program goes further than simply education and exercise. Above all, the principles of CBT where the role of thoughts, beliefs, and expectations are believed to have a major impact on symptoms [56–58] were followed. The present results support the effectiveness of an intervention based on CBT principles. For instance, in the qualitative interviews, the unconditional acceptance of group facilitators, the group cohesion and the empowerment over the illness had a significant impact on patients. Other authors (e.g., [59]) have also reported that facilitators, group membership and the sharing of strategies and life changes such as attitudes and healthy behaviours can significantly influence patients' pain recognition and their sense of control over their disease. Also, being part of an educational program appears to be helpful in reducing FMS patients' anxiety levels [60]. The present results are further in line with the 2012 Canadian Guidelines for the Diagnosis and Management of FMS [7,8] which specifically mention that multimodal management strategies must be used when dealing with FMS and that patients should play an active role in their care.

### Long-Term Impact of the Intervention

Although the absence of a WL group at the T_3_ and T_4_ follow-up times limits our ability to draw conclusions regarding the long-term impacts of the program, some interesting results deserve to be mentioned. First, long-term improvements (up to 12 months post-intervention) on the PGIC scales and perceived pain relief were found among the patients of the INT Group. In addition, the average pain intensity in the last week, impact of FMS on daily life, pain catastrophizing and some coping strategies also appeared to improve over time. As in most quasi-experimental studies, we cannot determine if these changes were due to the intervention, to the effect of time or simply to the mere participation in a study. Time effects (independent of the intervention) were found on different follow-up outcome measures which suggests that the participation in the study, taken alone, can bring improvement in the medical condition. These preliminary results support the long-term effect of the PASSAGE intervention, but future randomized controlled studies are nonetheless needed.

### Strengths and Limitations

To our knowledge, this study is the first which evaluates the clinical impact of a multicomponent interdisciplinary self-management intervention for FMS patients using a two-site mixed-methods design. Such a design (quantitative and qualitative) insured appropriate triangulation of the data [61], and allowed for a richer exploration of participants' experiences during the intervention. Other strengths to the present study which insure its internal validity deserve to be highlighted. A randomized controlled design was used, a rigorous training of intervention facilitators was carried out, standardized recruitment and data collection methods across study sites were used, validated and recommended measurement scales were utilized, and mixed models allowing the adjustment for confounders and the handling of missing data were used. Furthermore, this study was conducted on two different sites located in two different regions of the province of Québec (Canada) which ensures a better representativeness of FMS patients. Finally, the retention rate was good and in line with other intervention studies with this population (e.g., [62]). In the INT Group, 78.6% of the patients completed the intervention (18.8% of those in the WL Group dropped out during this period), 71.4% completed the measures at 3 months post-intervention, 67.9% did so 6 months post-intervention and finally 64.3% completed the 12-month follow-up questionnaire.

Some limitations to the present study should nonetheless be addressed. The sample size remained relatively small and might have limited the detection of some group differences (Type II error). Although a Bonferroni correction was applied in some analyses, the possibility of type I error resulting from the high number of statistical tests that were conducted cannot be excluded. Also, the actual usage of the strategies taught to the patients during the intervention was not assessed which limits our ability to determine in which way the strategies were helpful or not. Another limitation is the self-report nature of all the measures used in the present study. Future research should include methods such as observation-based assessments of patients' functioning [62].

Finally, although the baseline characteristics of the patients were similar to what is found in the general population of FMS patients, it should be remembered that the present RCT had strict selection criteria which may lead to the selection of patients that are not representative of the general population. For example, participants in the present study had to report moderate pain intensity (≥ 4/10) in the seven days prior to enrolment leading to the exclusion of a number of mild FMS patients. In fact, the recruitment of participants with pain ≥ 4/10 may explain the limited impact of the intervention on the 0–10 intensity of pain numeric scale considering the difficulty to improve outcomes in more severe FMS patients [13].

### Future Research and Clinical Implications

Our next step will be to evaluate the clinical impact of our interdisciplinary self-management intervention in a pragmatic trial conducted in many clinical settings of the province of Quebec, thus providing further evidence for the effectiveness of the intervention in “real-life” settings [63]. The PASSAGE Program could also be improved by involving family physicians in the intervention in order to provide the patients with optimal medication for their symptoms. It has been suggested that FMS patients should be treated rapidly in the primary sector of care before symptoms get worse [64] and that a shared-decision making process between the patient and the physician can help in improving the quality of the interactions [65]. Furthermore, in a recent report, Oldfield and colleagues [66] discussed how primary care physicians' moral judgements resulting from their skepticism regarding the legitimacy of FMS greatly impair the patient-doctor relationship for women with FMS. It is thus essential to better educate family physicians and insure that the patients get the treatment they need. In this regard, Fitzcharles and colleagues [7,8] published a set of recommendations in order to discuss the legitimacy of FMS and to guide its diagnosis and management. Thus, involving primary care physicians in the PASSAGE Program could be very beneficial for FMS patients in order to provide this often neglected clientele of patients with effective self-management of their symptoms.

In conclusion, our new multicomponent interdisciplinary self-management intervention for FMS was found to be effective in improving the patients’ global impression of change in terms of pain, functioning and QOL as well as in increasing their perceived pain relief. We suggest including this type of measures in future clinical trials on FMS as they appear to capture an important aspect of the patient experience. Further research on the long-term efficacy of the PASSAGE Program is nonetheless needed as well as studies involving primary care physicians in the intervention.

## Supporting Information

S1 CONSORT Checklist CONSORT Checklist. (PDF)

S1 Dataset Original data. (SAS7BDAT)

S1 Protocol Trial protocol (French). (PDF)

S2 Protocol Trial protocol methodology (English). (PDF)
