# Supplementary material for: Multicomponent Interdisciplinary Group Intervention for Self-Management of Fibromyalgia: A Mixed-Methods Randomized Controlled Trial
Source: PLoS One. 2015 May 15;10(5):e0126324. doi: 10.1371/journal.pone.0126324 (PMC4433106; doi:10.1371/journal.pone.0126324)
Supplement: S1 CONSORT Checklist — (PDF) [file pone.0126324.s001.pdf]

Checklist of Items for Reporting Trials of Nonpharmacologic Treatments*

Section	Item	Standard CONSORT Description	Extension for Nonpharmacologic Trials	Manuscript's sections
**Title and abstract†**	1	How participants were allocated to interventions (e.g., "random allocation," "randomized," or "randomly assigned")	In the abstract, description of the experimental treatment, comparator, care providers, centers, and blinding status	Title page and Abstract
**Introduction**				
Background	2	Scientific background and explanation of rationale		Introduction section
**Methods**				
Participants†	3	Eligibility criteria for participants and the settings and locations where the data were collected	When applicable, eligibility criteria for centers and those performing the interventions	Methods - Design and Settings section<br><br>Methods - Eligibility, Recruitment, and Randomization section
Interventions†	4	Precise details of the interventions intended for each group and how and when they were actually administered	Precise details of both the experimental treatment and comparator	Methods - Description of the Group Conditions section<br><br>Table 1
	4A		Description of the different components of the interventions and, when applicable, descriptions of the procedure for tailoring the interventions to individual participants	Methods - Description of the Group Conditions section<br><br>Table 1
	4B		Details of how the interventions were standardized	Methods - Description of the Group Conditions section

	4C		Details of how adherence of care providers with the protocol was assessed or enhanced	Methods - Description of the Group Conditions section
Objectives	5	Specific objectives and hypotheses		Introduction section
Outcomes	6	Clearly defined primary and secondary outcome measures and, when applicable, any methods used to enhance the quality of measurements (e.g., multiple observations, training of assessors)		Methods - Outcomes section
Sample size†	7	How sample size was determined and, when applicable, explanation of any interim analyses and stopping rules	When applicable, details of whether and how the clustering by care providers or centers was addressed	Methods - Sample Size section
Randomization–sequence generation†	8	Method used to generate the random allocation sequence, including details of any restriction (e.g., blocking, stratification)	When applicable, how care providers were allocated to each trial group	Methods - Eligibility, Recruitment, and Randomization section
Allocation concealment	9	Method used to implement the random allocation sequence (e.g., numbered containers or central telephone), clarifying whether the sequence was concealed until interventions were assigned		Methods - Eligibility, Recruitment, and Randomization section
Implementation	10	Who generated the allocation sequence, who enrolled participants, and who assigned participants to their groups		Methods - Eligibility, Recruitment, and Randomization section
Blinding (masking)†	11A	Whether or not participants, those administering the interventions, and those assessing the outcomes were blinded to group assignment	Whether or not those administering co-interventions were blinded to group assignment	*Not feasible except for the statistical analyses* Methods - Sample Size section
	11B		If blinded, method of blinding and description of the similarity of interventions†	*Not applicable*

Statistical methods†	12	Statistical methods used to compare groups for primary outcome(s); methods for additional analyses, such as subgroup analyses and adjusted analyses	When applicable, details of whether and how the clustering by care providers or centers was addressed	Methods - Quantitative Data Analysis section<br><br>Methods - Qualitative Data Analysis section
**Results**				
Participant flow†	13	Flow of participants through each stage (a diagram is strongly recommended)---specifically, for each group, report the numbers of participants randomly assigned, receiving intended treatment, completing the study protocol, and analyzed for the primary outcome; describe deviations from study as planned, together with reasons	The number of care providers or centers performing the intervention in each group and the number of patients treated by each care provider or in each center	Figure 1<br><br>Results - Participants' Recruitment section<br><br>Methods - Design and Settings section
Implementation of intervention†	New item		Details of the experimental treatment and comparator as they were implemented	Methods - Description of the Group Conditions section<br><br>Table 1
Recruitment	14	Dates defining the periods of recruitment and follow-up		Methods - Eligibility, Recruitment, and Randomization section<br><br>Methods – Procedure section

Baseline data†	15	Baseline demographic and clinical characteristics of each group	When applicable, a description of care providers (case volume, qualification, expertise, etc.) and centers (volume) in each group	Results – Participants' Characteristics section<br><br>Methods - Description of the Group Conditions section<br><br>Table 2
Numbers analyzed	16	Number of participants (denominator) in each group included in each analysis and whether analysis was by "intention-to-treat"; state the results in absolute numbers when feasible (e.g., 10/20, not 50%)		Figure 1<br>Results<br>Table 2<br>Table 3<br><br>Methods - Quantitative Data Analysis section
Outcomes and estimation	17	For each primary and secondary outcome, a summary of results for each group and the estimated effect size and its precision (e.g., 95% confidence interval)		Results section<br>Table 3
Ancillary analyses	18	Address multiplicity by reporting any other analyses performed, including subgroup analyses and adjusted analyses, indicating those prespecified and those exploratory		Methods - Quantitative Data Analysis section<br><br>Methods - Qualitative Data Analysis section
Adverse events	19	All important adverse events or side effects in each intervention group		*Not applicable*
**Discussion**				
Interpretation†	20	Interpretation of the results, taking into account study hypotheses, sources of potential bias or imprecision, and the dangers associated with multiplicity of analyses and outcomes	In addition, take into account the choice of the comparator, lack of or partial blinding, and unequal expertise of care providers or centers in each group	Discussion section

Generalizability†	21	Generalizability (external validity) of the trial findings	Generalizability (external validity) of the trial findings according to the intervention, comparators, patients, and care providers and centers involved in the trial	Discussion - Strengths and Limitations section
Overall evidence	22	General interpretation of the results in the context of current evidence		Discussion section

*Additions or modifications to the CONSORT checklist. CONSORT = Consolidated Standards of Reporting Trials.

†This item was modified in the 2007 revised version of the CONSORT checklist.
