# Supplementary material for: Multicomponent Interdisciplinary Group Intervention for Self-Management of Fibromyalgia: A Mixed-Methods Randomized Controlled Trial
Source: PLoS One. 2015 May 15;10(5):e0126324. doi: 10.1371/journal.pone.0126324 (PMC4433106; doi:10.1371/journal.pone.0126324)
Supplement: S1 Protocol — (PDF) [file pone.0126324.s003.pdf]

DÉVELOPPEMENT, IMPLANTATION ET ÉVALUATION D'INTERVENTIONS

DE TYPE «ÉCOLE INTERACTIONNELLE» ADAPTÉES À DES

PERSONNES SOUFFRANT DE FIBROMYALGIE OU DE LOMBALGIE

Protocole de recherche
volet 3-A — programme ACCORD

Chercheurs principaux :	Patricia Bourgault, inf., Ph.D.; Serge Marchand, Ph.D.
Chercheurs collaborateurs :	Isabelle Gaumond, Ph.D., Jacques Charest, Ph.D., Manon Choinière, Ph.D., Dominique Dion, M.D., M.Sc., Anaïs Lacasse, Ph.D. Juliana Barcellos de Souza, Ph.D.,
Partenaires-cliniciens :	Christian Cloutier, M.D., CHUS<br>Sylvie Lamoureux, psychologue, CHUS<br>Émilie Lagueux, ergothérapeute, CHUS<br>Mario Sian et Serge Favreau , psychologues, UQAT
Partenaire-patient :	Diane Leroux<br>Représentante de l'Association de fibromyalgie de l'Estrie
Partenaires-gestionnaires :	Madeleine Ducharme, CHUS<br>Ghyslaine Pratta, Centre de réadaptation Constance-Lethbridge

Organisme subventionnaire :	IRSC

Table des matières

1. RÉSUMÉ DU PROJET 4
2. PROBLÉMATIQUE ET RECENSION DES ÉCRITS 5
2.1 Problématique 5
2.2 Recension des écrits 6
2.2.1 Fibromyalgie 6
2.2.2 Lombalgie 6
2.2.3 Conséquences de la douleur chronique 7
2.2.4 Programmes d'intervention 8
2.3 Cadre théorique: un model interactionnel 10
3. OBJECTIFS DE L'ÉTUDE 12
4. MÉTHODOLOGIE DE L'ÉTUDE 12
4.1 Devis de recherche 12
4.2 Population 13
4.3 Critères de sélection 13
4.3.1 Critères d'inclusion 13
4.3.2 Critères d'exclusion 14
4.4 Déroulement de l'étude et collecte des données 14
4.4.1 Formation du personnel de recherche et des intervenants 14
4.4.2 Procédure de recrutement et collecte des données 14
4.5 Variables et instruments d'évaluation 16
4.5.1 Variables indépendantes 16
4.5.2 Variables dépendantes et instruments de mesure utilisés auprès des participants 20
4.5.3 Variables dépendantes et instruments de mesure utilisés auprès des conjoints des participants 22
4.5.4 Variables et mesures spécifiques à l'analyse économique 23
4.5.5 Suivi de l'implantation 24
4.6 Analyses statistiques 24
4.6.1 Estimation de la taille échantillonnale 24
4.6.2 Analyses des données 25
4.7 Considérations éthiques 25
5. RETOMBÉES ET IMPACTS DU PROJET 26
6. ÉCHÉANCIER 27
7. RÉFÉRENCES 28
Annexes 39

Liste des abréviations

AQDC : Association québécoise de la douleur chronique
APS: American Pain Society
DC : Douleur chronique
EI : École Interactionnelle
ÉIF : École Interactionnelle de fibromyalgie
ÉIL : École Interactionnelle de lombalgie
FM : Fibromyalgie
IASP: International Association for the Study of Pain
LB : Lombalgie
UdeS : Université de Sherbrooke
UQAT : Université du Québec en Abitibi-Témiscamingue

1. RESUME DU PROJET

Un nombre important de personnes souffrent de douleur chronique (DC) et la prévalence ne cesse de s'accroître. Bien qu'il n'existe pas de statistiques précises pour la province de Québec, les estimations les plus conservatrices situent la prévalence de la DC aux alentours de 20% dans la population en générale au Canada (Millar, 1996; Moulin, Clark, Speechley et Morley-Forster, 2002, Boulanger *et al*., 2007) et ailleurs dans le monde (Breivik, Collett, Ventafridda, Coheh, Gallacher, 2006; Passik, Kirsh, McDonald, Russak, Martin *et al*., 2000). Plusieurs études témoignent de douleurs mal diagnostiquées, non traitées ou insuffisamment soulagées qui engendrent des conséquences dramatiques aux plans physique, psychosocial et économique (Ashburn et Staats, 1999; Gureje, Von Korff, Simon et Gater, 1998; Ohayon et Schatzberg, 2003). Le principal obstacle au traitement optimal de la douleur chronique est qu'elle n'est généralement pas reconnue comme une maladie au même titre que d'autres désordres chroniques et qu'elle ne reçoit pas, par conséquent, l'attention requise au plan clinique.

Au cours des dernières années, des cliniciens et chercheurs de l'Université de Sherbrooke (UdeS), de l'Université du Québec en Abitibi-Témiscamingue (UQAT) et du Centre de réadaptation du Constance Lethbridge ont mis sur pied des interventions faisant appel à des notions d'auto-gestion de la DC et d'autonomisation du patient (*patient empowerment)* et s'adressant aux personnes souffrant de fibromyalgie ou de lombalgie plus spécifiquement appelées « Écoles Interactionnelles » (EI) par l'UdeS et l'UQAT. Quatre caractéristiques principales distinguent les EI des autres programmes longs de prise en charge : équipe, objectifs pédagogiques, buts thérapeutiques et auto-gestion de la douleur. Les EI impliquent une approche multifactorielle et interdisciplinaire portant sur des aspects physiques et psychologiques de la DC et donnent une part active aux participants en favorisant l'utilisation de diverses stratégies d'auto-gestion visant à contrôler leur condition. Les résultats cliniques obtenus jusqu'à maintenant avec ce type d'intervention sont suffisamment prometteurs pour proposer ici, de réunir les expertises des patients, des cliniciens et des chercheurs afin de jumeler les forces des interventions développées à ce jour tout en tenant compte d'éléments clés provenant des écrits scientifiques plus récents. Ce projet s'inscrit dans une initiative plus large de recherche dans le domaine de l'échange et du transfert des connaissances **(Programme ACCORD: Application Concertée des COnnaissances et Ressources en Douleur)**, initiative subventionnée par les Instituts de recherche en santé du Canada (IRSC). Le Programme ACCORD vise essentiellement à améliorer l'évaluation et le traitement de la douleur chronique au Québec à l'aide de stratégies efficaces d'échange et d'application de connaissances.

Le présent protocole concerne la Phase 1 du volet 3-A du Programme ACCORD. Ce volet cible les consommateurs/grand public et les personnes qui souffrent de DC. Plus spécifiquement, l'objectif de ce volet vise à développer et à mesurer l'efficacité d'une intervention de type « école interactionnelle » auprès de personnes souffrant de fibromyalgie (FM) ou de lombalgie (LB). Ces Écoles

interactionnelles seront implantées à l'École de Réadaptation de l'Université de Sherbrooke ainsi qu'à l'Université du Québec en Abitibi-Témiscamingue. Leur impact et leur efficacité seront évalués aux plans clinique et économique. Les modèles d'interventions qui en découleront serviront de guide et de référence pour l'implantation des ÉI dans une vingtaine de centres de réadaptation du Québec d'ici les cinq prochaines années, soit la phase 2 du volet 3A. Le déploiement de ces EI devrait contribuer à l'amélioration de la condition et de la qualité de vie des personnes qui souffrent de FM ou de LB et ce, en leur donnant un rôle de partenaire actif dans la gestion de leur état de santé.

2. PROBLÉMATIQUE ET RECENSION DES ÉCRITS

2.1 PROBLÉMATIQUE

Au Québec et au Canada, on estime qu'environ un individu sur cinq souffre de douleur chronique (DC), et ce nombre augmente de façon alarmante (Moulin *et al*., 2002, Statistiques Canada, 2002, Boulanger *et al*., 2007). L'étude de Moulin, Clark, Speechley et Morley-Forster (2002) a montré que 29% des répondants souffraient de DC et que la prévalence augmente avec l'âge. Selon la définition proposée par l'International Association for the Study of Pain (IASP), la douleur est une expérience sensorielle et émotionnelle désagréable, associée à un dommage tissulaire réel ou potentiel ou décrite en de tels termes (Merskey et Bogduk, 1994). Étant à la fois une expérience sensorielle et émotionnelle sa compréhension en est complexifiée, puisque, malgré qu'elle soit une sensation universelle, elle relève de la subjectivité de chacun. La douleur est une perception qui est habituellement considérée comme une expérience négative et désagréable bien qu'essentielle à la survie. La douleur aiguë est d'apparition récente et survient en général en réponse à des stimulations dommageables pour les tissus, comme par exemple une brûlure, une piqûre ou encore une coupure (Besson, 1992). Toutefois, la douleur peut outrepasser sa fonction de système d'alarme et persister au-delà du recouvrement normal. C'est alors que la douleur devient chronique et qu'elle représente un véritable problème pour les personnes qui en sont atteintes. On admet arbitrairement qu'une douleur devient chronique lorsqu'elle perdure au-delà de trois à six mois (Melzack et Wall, 1988). Plus récemment, Loeser et Melzack (1999) ont souligné que la douleur chronique se distingue de la douleur aigüe non pas par la durée ou la persistance de la douleur mais plutôt par l'incapacité du corps à restaurer ses fonctions physiologiques pour arriver à rétablir l'homéostasie.

Longtemps considérée comme un symptôme, et non pas comme une maladie, la prise en charge de la douleur chronique demeure souvent sous-optimale (American Pain Society, 2003; Breivik, Collett, Ventafridda, Cohen et Gallacher, 2006). Les coûts engendrés par la douleur chronique au Québec et au Canada, en terme de journées de travail perdues et d'utilisation de ressources de santé, demeurent mal chiffrés mais des études réalisées au Canada (Moulin, Clark, Speechley et Morley-Foster, 2002; Millar, 1996; Schopflocher, Borowski, Harstall, Juzwishin, Ospina et Taenzer, 2003) et dans d'autres pays (American Pain Society, 2003; Kumpusalo, Mantyselka et Takala, 2000; Loeser, 1999; van Tulder, Koes et Bouter, 1995; Stewart, Ricci, Chee, Morganstein et Lipton, 2003) révèlent

que les coûts directs et indirects sont énormes. À titre d'exemple, en Finlande, on estime que les coûts reliés à la DC (soins de santé et indemnités) représenteraient plus que 3% du PNB (Kumpusalo, Mantyselka et Takala, 2000). En plus des coûts économiques directs et indirects qu'elle engendre, cette condition a d'importantes conséquences au plan biospychosocial et en termes de limitations tant pour l'individu que pour ses proches (American Pain Society, 2003; Association québécoise de la douleur chronique (AQDC), 2005; Breivik *et al*., 2006, Moulin *et al*. 2002).

2.2. Recension des écrits

2.2.1 Fibromyalgie

La FM est un syndrome complexe incluant de nombreuses manifestations cliniques et une étiologie mal comprise (Block, 1999; Houvenagel, 2003). Le tableau clinique de la FM se compose de douleurs chroniques diffuses, de douleurs localisées (points de sensibilité), d'une augmentation de la perception de la douleur (hyperalgésie), de réponses anormales aux stimuli mécaniques (allodynie), de troubles du sommeil, de fatigue, de perte de force musculaire, de dépression et parfois de troubles digestifs comme le syndrome du côlon irritable (Bennett, 2003). Cependant, pour établir le diagnostic, seulement les deux premiers symptômes cités ci-dessus sont nécessaires (Houverngel, 2003; Wolf *et al*., 1990). De plus, selon Berman *et al*. (1999) et White *et al*. (1999), la DC est le principal facteur de plainte. La fibromyalgie (FM) touche environ 3 à 5 % de la population (Clauw et Crofford, 2003) avec une prédominance féminine à 80% selon Hirsch-Herpers (2001). Ce syndrome de douleur chronique affecte plus de 630 000 Canadiens (McNally, Matheson et Bakowsky, 2006).

2.2.2 Lombalgie

La lombalgie (LB) réfère à des douleurs au bas du dos attribuables principalement à des problèmes musculo-squelettique et représente la 2e cause motivant les gens à consulter un professionnel de la santé. Aussi, il s'avère que plus de 90% des personnes souffriront un jour d'un mal de dos et ce, souvent après la vingtaine et dans un contexte de sédentarité (Mantha et Lefrançois, 2007). Trois types de lombalgie sont généralement considérés aux fins de triage : la lombalgie simple, la lombalgie avec composante neurologique et la lombalgie avec pathologie rachidienne (Rossignol et Arsenault, 2006). Dans le cadre de cette étude, seul la lombalgie simple sera considérée. Les caractéristiques générales de ce type de lombalgie sont l'absence d'une composante neurologique à la douleur lombaire ou lombosacrée, la présence d'une douleur « mécanique » c'est-à-dire qui varie dans le temps et selon l'activité et stabilité de l'état général de la personne lombalgique. Les douleurs au dos représentent 17% des problèmes de santé chroniques (Gouvernement du Québec, 2006). La prévalence à vie de la LB varie entre 70 et 85% et la lombalgie chronique invalidante touche environ 1% de la population (Kopec, Sayre et Esdaile, 2003). La LB entraîne donc des coûts importants reliés soit au traitement ou encore reliés à l'absentéisme au travail. En effet, au Québec, au cours des dernières années, la LB a amené des dépenses de près de six cent millions de dollars (Dionne, Bourbonnais, Frémont, Rossignol et Stock, 2004).

Parmi les facteurs à évaluer en lien avec la douleur chez les lombalgiques, Gatchel et Turk (1999) soulignent l'importance de considérer non seulement les aspects physiques et biomécaniques de la douleur mais aussi de porter une attention aux facteurs d'ordre psychosociaux. Les causes de la douleur lombaire chronique sont difficiles à déterminer et les personnes affligées par la LB doivent envisager apporter des changements dans leur mode de vie (Mantha et Lefrançois, 2007).

2.2.3 Conséquences de la douleur chronique

La DC entraîne des conséquences dans différentes sphères soit au niveau biologique, psychologique ou encore socio-familial.

Au niveau biologique, le sommeil est souvent perturbé par la DC et vice versa. Il est possible que les troubles du sommeil soient inhérents à la FM et ce, peu importe la sévérité de la douleur. Par contre, Affleck *et al*. (1996) ont montré qu'il y avait une relation bidirectionnelle entre la DC et la qualité du sommeil. Diverses études établissent une perturbation du patron du sommeil chez les fibromyalgiques (Arsenault et Marchand, 2007). La durée de la phase 1 (sommeil léger) est augmentée, le ratio alpha/delta est anormal, avec une nette accentuation des intrusions alpha lors du sommeil lent profond correspondant à un phénomène d'éveil (Dauvilliers et Touchon, 2001; Branco, Atalaia et Paiva, 1994). La latence d'endormissement se trouve alors prolongée et un raccourcissement de la durée totale du sommeil sont fréquemment observées. Chez les fibromyalgiques, les courbes d'enregistrement démontrent une baisse de l'index d'efficacité du sommeil (temps de sommeil effectif par rapport à la durée d'enregistrement). Le sommeil, plus court, apparaît de moins bonne qualité. La réduction de la phase 4 du sommeil, phase du sommeil lent et profond, pourrait expliquer la diminution du taux de somathormone (GH), qui pourrait, elle-même, perturber la sécrétion de bêta-endorphines et réduire la sensation de bien-être (Maquet, Croisier et Crielaard, 2001). Chez les lombalgiques, une étude cas-témoins (n=198) (Marty *et al*. 2004) a démontré que l'existence d'une lombalgie chronique est associée à une altération significative du sommeil des patients par rapport aux témoins. La moyenne du score de l'échelle de Pittsburgh, qui évalue la qualité du sommeil et dont le score varie de 0 (pas de désordre) à 21, était de $4,7 \pm 3,2$ pour les sujets sains et de $10,9 \pm 7,9$ pour les patients lombalgiques chroniques ($p < 0,0001$). Cependant, l'étude ne permet pas de conclure si les troubles du sommeil sont une cause ou une conséquence de la lombalgie chronique.

Au niveau psychologique, de nombreuses études épidémiologiques et cliniques (Gureje *et al*., 1998; Blyth *et al*., 2001; Ohayon *et al.,* 2003; McWilliams *et al.,* 2003; Arnow *et al*., 2006) ont documenté la forte prévalence de co-morbidités psychologiques chez les patients qui souffrent de DC et notamment lorsque cette dernière interfère avec les activités quotidiennes. Ainsi, une vaste étude multicentrique parrainée par l'OMS et menée sur cinq continents a montré que les patients souffrant de DC sont quatre fois plus à risque de souffrir également de dépression ou de troubles anxieux (Gureje *et al.,* 1998). La DC ressentie par les fibromyalgiques peut mener à des changements comportementaux ainsi qu'à une augmentation de l'anxiété et de la peur qui augmentent l'attention portée sur la

douleur ce qui en exacerbe la perception (Craig, 2003; Wall, 2003). De plus, la détresse psychologique et les troubles affectifs apparaissent fréquents avec une réduction des seuils de perception et de tolérance à la douleur. La coexistence de la FM et d'un syndrome dépressif est de plus régulièrement observée. La douleur chez les fibromyalgiques joue aussi un rôle important sur leur qualité de vie. Dans le même ordre d'idées, la douleur chronique ressentie chez les lombalgiques peut devenir la cause de celle-ci dans le contexte où le lombalgique tente d'éviter toute activité et s'enferme donc dans une boucle sans fin (Charest, Chénard, Lavignolle et Marchand, 1996). Cette causalité circulaire sera abordée davantage à la section 2.3. Sullivan *et al. (*2001) ajoutent que la tendance à la dramatisation (*catastrophizing*) face à la douleur constitue un prédicteur fiable et important de la sévérité de la douleur. Ces mêmes auteurs soulignent que cette tendance à la dramatisation peut être non seulement un concept lié au trait de personnalité et donc stable dans le temps, mais peut être également vue comme une réponse cognitive modifiable à l'aide d'interventions ciblées.

Au niveau socio-familial, l'influence du contexte socio-familial sur la douleur a été largement démontrée à ce jour (Romano, Turner et Jensen, 1997). En effet, les écrits indiquent l'importance du contexte socio-familial sur la douleur chronique ressentie de même que les impacts négatifs vécus par les proches d'une personne ayant une douleur chronique tel l'anxiété, la dépression et les conflits conjugaux (Schwartz, Slater, Birchler et Atkinson, 1991; Snelling, 1994 ; Silver, 2004). Schwartz, Slater, Birchler et Atkinson (1996) ont évalué le comportement de 61 hommes affectés d'une douleur rachidienne et ont observé que les conflits conjugaux génèrent une aggravation du handicap social et ont un impact psychologique chez le malade. Une bonne harmonie dans le couple et le soutien de l'environnement familial sont des déterminants en regard du comportement de la personne ayant une douleur face à celle-ci. La douleur chronique entraîne des conséquences au niveau de la cellule familiale telles que l'isolement social, les conflits conjugaux, une diminution des relations sexuelles et de l'anxiété (Snellings, 1994). Les résultats de l'étude de Schwartz *et al*. (1996), menée auprès de 29 couples, ont démontré que 28% des conjointes de lombalgiques chroniques présentaient des symptômes dépressifs. Dans cette étude, trois prédicteurs significatifs de la dépression chez les conjointes des lombalgiques ont été identifiés : niveau de douleur du malade, son niveau d'hostilité et de colère et le niveau de satisfaction de la relation conjugale telle que perçu par la conjointe. En outre, la douleur chronique a inévitablement un impact sur la vie familiale. Les membres de la famille se sentent souvent impuissants et inadéquats, surtout s'ils ont l'impression de ne recevoir que peu d'aide et de soutien extérieur (Vannotti et Gennart, 2006). Or, à notre connaissance, aucune étude n'a considéré l'implication de la famille dans l'intervention auprès d'une personne souffrant de FM. Kerns (In Gatchel et Turk, 1999) souligne l'importance de poursuivre les efforts pour développer et évaluer des interventions en considérant aussi la famille.

2.2.4 Programmes d'intervention

Plusieurs études ont démontré que des programmes d'intervention visant à rendre les personnes plus autonomes et impliquées dans la gestion de leur maladie

chronique peuvent avoir des impacts positifs sur leur qualité de vie et sur divers indicateurs de santé (Lorig, Ritter, Stewart, Sobel, Brown, Bandura, 2001; Bodenheimer, Lorig, Holman, Grumbach, 2002). La participation des patients à titre de partenaires actifs est de plus en plus perçue comme un des éléments-clés des modèles de soins en gestion de la maladie chronique (Anderson et Funnell, 2005; Watson, Broemeling, Reid et Black, 2004). Certains programmes sont maintenant offerts pour répondre aux besoins de personnes atteintes de problèmes chroniques spécifiques tels l'asthme (Réseau Québécois de l'Asthme et de la MPOC, 2007), le diabète (Anderson et Funnell, 2005; Diabète Québec, 2007), l'arthrite (Lorig, Gonzalez, Laurent, Morgan et Laris, 1998; La Société d'arthrite, 2007) alors que d'autres sont plus généraux (Stantford University, 2007). S'appuyant sur les mêmes principes, des programmes d'auto-gestion de la DC (Lefort, Gray-Donald, Rowat et Jeans, 1998; Gatchel et Okifuji, 2006) ont vu le jour, mais ne sont souvent disponibles que dans des centres spécialisés en douleur et donc peu accessibles à l'ensemble des gens, particulièrement hors des grands centres urbains.

Des études portant sur des modèles d'interventions pour les fibromyalgiques ont été développées et testées. Ces interventions visent surtout à améliorer la qualité de vie par l'activité physique (Valim *et al*., 2003; Vierck *et al*., 2001; Wigers, 1996), par des séances de relaxation ainsi que par des approches cognitives (Williams *et al*., 2002; Williams, 2002; Hadhazy *et al*., 2000) et éducationnelles (Mannerkorpi *et al*., 2000). Les bénéfices de tous ces traitements sont significatifs à court terme, mais ils ne semblent pas offrir un impact positif à long terme (Krsnich-Shriwise, 1997). De plus, Clauw *et al*. (2003) et Mannerkorpi *et al*. (2003) suggèrent que pour atteindre des résultats encore plus efficaces des interventions multifactorielles devraient être considérées (Bennett *et al*., 1996). Ces interventions multifactorielles devraient aborder simultanément les aspects physiques, psychologiques et sociaux de la maladie et intégrer des stratégies d'auto-gestion de la douleur.

D'autre part, le fréquent retour des symptômes de la FM observé quelques mois après la fin d'une intervention pourrait être lié à la dépendance du participant face au traitement et au thérapeute (Clauw *et al*., 2003 ; Picavet *et al*., 2003). Ce phénomène de dépendance peut indiquer un manque d'intégration des solutions proposées pour soulager la douleur chronique. Certaines techniques de prise en charge de la douleur chronique ont tenté de maximiser l'intégration des solutions par le participant. Parmi ces interventions, il existe les *Écoles du dos* (Poiraudeau *et al*., 2001 ; Hodselmans *et al*., 2001 ; Roques *et al*., 2002 ; Chatain *et al*., 1998 ; Charest *et al*., 1994 ; Chenard *et al*., 1991) qui ont démontré leur efficacité à long terme avec les personnes souffrant d'une douleur chronique au niveau lombaire. Les résultats de l'étude de Barcellos de Souza (2007), dans laquelle une intervention multifactorielle multidisciplinaire structurée pour les fibromyalgiques, appelée « École interactionnelle de fibromyalgie-ÉIF) a été développée et évaluée, démontrent que cette intervention peut entraîner des changements durables (un an après la fin de l'intervention). Ces changements s'observent tant sur le plan des données expérimentales (réduction de l'allodynie mécanique, augmentation du

seuil de douleur aux points de sensibilité) que sur le plan des données cliniques (réduction de l'impact de la FM et de la douleur clinique). Ces résultats indiquent non seulement que l'amélioration est significative mais qu'elle persiste après le traitement alors que le taux d'abandon du programme a été de 3%. Toutefois, cette étude présentait certaines failles méthodologiques de sorte qu'il est difficile d'arriver à des conclusions claires notamment l'absence de groupe contrôle.

En ce qui concerne l'efficacité des programmes auprès de personnes ayant une lombalgie chronique, Rossignol et Arsenault (2006) mentionnent que l'approche multidisciplinaire et la thérapie comportementale détiennent un niveau élevé de preuve scientifique d'efficacité et l'École de dos, un niveau modéré. Bien que modéré, ce type d'approche a une efficacité bien supérieure aux approches médicamenteuses couramment utilisées (Rossignol et Arsenault, 2006). Les programmes longs de prise en charge font partie du troisième niveau de traitements éducationnels pour lutter contre la lombalgie. L'École interactionnelle de lombalgie (ÉIL) fait partie de cette catégorie (Charest, 1996).

2.3 CADRE THÉORIQUE : UN MODÈLE INTERACTIONNEL

Les Écoles Interactionnelles (ÉI) reposent sur un programme structuré pour traiter, en groupe, la fibromyalgie ou la lombalgie. Leur but est d'apprendre aux personnes fibromyalgiques (ÉIF) ou lombalgiques (ÉIL) à s'auto-soigner. L'intervention des ÉI s'inspire du modèle interactionnel et se base sur les principes de la thérapie de stratégie et de la thérapie brève. Ce modèle d'intervention prend en compte : (a) le modèle circulaire de la douleur, qui fait ressortir l'interaction entre toutes les composantes de la douleur : nociceptive, sensorio-discriminative, motivo-affective et comportementale (Marchand, 1998) ; (b) les principes de la thérapie brève et de la thérapie stratégique (voir revue par Charest, 1998, Charest, 1996, Watzlawick et Nardone, 2000). La stratégie d'intervention des EI se fonde sur l'étude des comportements communicationnels des individus en interaction (Foerster, 1988).

Le programme des EI repose sur les trois caractéristiques suivantes : interaction, tactiques thérapeutiques et déroulement (Charest, Chénard, Lavignolle et Marchand, 1996). En ce qui concerne la première caractéristique, l'interaction, l'alliance thérapeutique ou la qualité de la relation constitue un facteur déterminant des progrès thérapeutiques. La façon dont la personne perçoit les divers aspects de cette relation, tels que le respect, la cordialité et l'engagement du praticien, expliquerait environ 25% des changements thérapeutiques (Asay et Lambert, 1999). Les praticiens, toujours les mêmes pour chacune des rencontres de groupe, maximisent l'alliance thérapeutique par divers moyens dont par exemple le contrat thérapeutique, les conversations informelles durant les pauses et les activités physiques personnalisées à chacun des participant. En ce qui a trait à la deuxième caractéristique, soient les tactiques thérapeutiques, elles représentent des lignes directrices sur lesquelles les praticiens s'appuient pour mettre en œuvre la stratégie interactionnelle. Une tactique thérapeutique désigne un ensemble de moyens coordonnés et pratiques visant à atteindre le but de s'auto-soigner. Ces

tactiques modifient la perception qu'a la personne d'elle-même et l'amène à sortir de son processus de victimisation en regard de la fibromyalgie ou de lombalgie pour en arriver à être en mesure de mieux composer avec ce problème. Les trois principales tactiques sont : client ou touriste, changement minimal et expert, non expert ou égalitaire. Ces tactiques s'adressant au plan moteur et cognitif de la personne et ont été décrites en détail par Charest, Chenard, Lavignolle et Marchand (1996) en regard d'une population lombalgique (Chenard, *et al*., 1991; Charest, *et al*., 1994). La troisième caractéristique, soit le déroulement, réfère aux rencontres hebdomadaires des EI soient neuf rencontres, entrecoupées de deux semaines de travail autonome, pour un total de onze semaines, organisées autour des thèmes suivants : contrat, symptômes, préparation physique, force mentale, gestion de ses capacités, nutrition, chronicité, traitement, cheminement et relance. En résumé, cette intervention non-pharmacologique offre aux participants des informations à propos de leur maladie, des cercles vicieux auxquels ils sont soumis, ainsi qu'a des prescriptions de tâches physiques (exercices) et psychologiques (relaxation, stratégies de gestion des limites, et recadrage) pour favoriser une autonomie face à la gestion de leur douleur.

Dans le cadre d'une École Interactionnelle s'adressant à une population fibromyalgique, Barcellos de Souza (2007) a intégré à l'intervention les trois caractéristiques décrites précédemment, soit l'interaction, les tactiques thérapeutiques et le déroulement. Dans cette étude, deux nouveaux contenus avaient alors été ajoutés à l'intervention par rapport aux programmes multidisciplinaires conventionnels de gestion de douleur. Le premier ajout concernait la pratique de la respiration lente et profonde appelée aussi diaphragmatique. Cette respiration favorise la réduction de l'anxiété, de la dépression, du stress, de la douleur et de la perception chez la personne d'une amélioration de son état de santé général (Villien, Yu, Barthelemy et Jammes, 2005). Le second contenu ajouté concernait la nutrition, un volet rarement décrit comme outil de traitement de la fibromyalgie (Lemstra et Olszynski, 2005). Les résultats obtenus se sont avérés positifs dans une perspective de gestion de la douleur (Barcellos de Souza, Charest et Marchand, 2007).

Tel que mentionné auparavant, les écrits soulignent l'impact de la famille sur la gestion de la douleur ou encore les conséquences négatives de la douleur chronique sur la famille. Or, bien que plusieurs études indiquent la nécessité d'inclure la famille dans les programmes de gestion de la douleur, aucune étude, à notre connaissance, n'évaluée l'impact d'une telle intervention sur les conjoints. La présente étude propose d'évaluer si les ÉI peuvent avoir un effet positif sur le conjoint.

3. OBJECTIFS DE L`ÉTUDE

Objectif primaire :

Évaluer l'efficacité à court et à long terme d'interventions multifactorielles et interdisciplinaires de type École interactionnelle de fibromyalgie (ÉIF) et de lombalgie (ÉIL) pour réduire la sévérité de la douleur chez des personnes souffrant de ces syndromes de douleur chronique.

Objectifs secondaires :

a) Évaluer si les ÉIF et ÉIL contribuent à court et à long terme à :
- o Diminuer l'impact fonctionnel de la douleur dans diverses sphères de la vie quotidienne;
- o Réduire la tendance à la dramatisation face à la douleur;
- o Améliorer la qualité du sommeil, le bien-être psychologique et la qualité de vie reliée à la santé;
- o Diminuer les niveaux d'anxiété et de dépression chez les conjoints et augmenter leur qualité de vie et leur niveau de sentiment d'efficacité et de santé perçue.

b) Obtenir des données préliminaires sur l'impact des ÉIF et ÉIL au plan économique—i.e., en termes des coûts reliés à la douleur et à l'utilisation des ressources de santé.

c) Créer un modèle d'implantation des ÉIF/ÉIL qui servira de plate-forme à la Phase 2 du présent projet où les EI seront offerts dans des centres de réadaptation du Québec

4. MÉTHODOLOGIE DE L'ÉTUDE

4.1 Devis de recherche

Pour répondre aux objectifs décrits précédemment, un devis de recherche de type essai randomisé contrôlé avec mesures répétées sera utilisé. Chaque type d'école (ÉIF, ÉIL) fera l'objet d'un essai randomisé contrôlé multicentrique (Sites : École de Réadaptation de l'Université de Sherbrooke et Université du Québec en Abitibi-Témiscamingue (UQAT)). La réalisation d'une étude multicentrique permettra d'établir les conditions nécessaires d'uniformisation des procédures en vue d'une éventuelle implantation des EI dans d'autres sites à travers le Québec.

Les participants souffrant de FM ou de LB seront randomisés dans chacun des sites d'étude à l'aide d'une table de nombres aléatoires. Ils seront assignés soit à participer aux EI (Groupe Intervention) soit à être sur une liste d'attente (Groupe contrôle). La randomisation sera effectuée par la méthode de randomisation stratifiée afin d'apparier les sujets des groupes intervention et contrôle selon qu'ils souffrent de douleur d'intensité modérée (4-6/10 sur une échelle numérique où 0 = aucune douleur et 10 = la pire douleur possible) ou sévère (≥ 7/10) selon la même échelle dans les sept jours précédant l'évaluation initiale. Les sujets seront aussi

appariés selon le sexe. À noter que les groupes FM (intervention ou contrôle) ne seront constitués que de sujets de sexe féminin compte tenu du ratio femmes/hommes (6/1) pour ce type de désordre (McNally *et al*., 2006). Une telle stratégie permettra de limiter les sources de variabilité potentiellement reliées au sexe.

Les participants recevant l'intervention (EIF/EIL) seront suivis sur une période de 12 mois alors que ceux du Groupe contrôle le seront sur une période de six mois afin qu'ils puissent avoir accès à l'intervention (ÉIF/ÉIL) dans un délai jugé éthiquement raisonnable.

4.2 Population

Personnes adultes de la région de Sherbrooke ou de Rouyn-Noranda présentant une problématique de fibromyalgie ou de lombalgie chronique.

4.3 Critères de sélection

Un nombre égal de sujets sera enrôlé au sein de chaque site d'étude et ce, à partir des critères d'inclusion/exclusion suivants :

4.3.1 Critères d'inclusion. Les sujets devront répondre à <u>tous</u> les critères suivants pour être enrôlés dans l'étude :

- Avoir un diagnostic médical de FM primaire posé depuis au moins 6 mois et établi à partir des critères de l'American College of Rheumatology (Wolfe *et al*., 1990) —i.e., 11/18 points de sensibilité à la douleur générée par une pression équivalente à 4kg (Wolfe *et al*., 1990).

  OU

  Avoir un diagnostic médical de LB non spécifique posé depuis au moins 6 mois et établi selon les critères de Chou (2007).
- Être âgé de 18 à 65 ans.
- Être capable de lire, comprendre et compléter des questionnaires en français.
- Rapporter une douleur d'intensité moyenne $\geq$ 4/10 au cours des 7 jours précédant l'évaluation initiale.
- Accepter d'être assigné au hasard à l'une ou l'autre des conditions expérimentales.
- Être intéressé et motivé par le type d'intervention proposé (client vs touriste : Un client, au sens où nous l'employons ici, désigne la personne fibromyalgique ou lombalgique qui demande activement de l'aide et répond aux critères médicaux. La tactique thérapeutique consiste à évaluer si la personne est « cliente » ou, dans le cas d'une « touriste », à la rendre cliente (Barcellos de Souza, 2007, p.213).
- Accepter de participer à 9 rencontres hebdomadaires étalées sur une période de 11 semaines et à une visite de relance.
- Maintenir un traitement médicamenteux contre la douleur stable ou ne prendre aucun analgésique pour la durée de l'étude.

4.3.2 Critères d'exclusion. Les sujets seront exclus de l'étude s'ils répondent à n'importe lequel des critères suivants :

- o Être enceinte ou en phase d'allaitement.
- o Être atteint d'un cancer actif ou d'une maladie d'origine métabolique non contrôlée.
- o Souffrir de douleur chronique d'origine autre que FM ou LB (p.ex. : arthrite rhumatoïde, neuropathie diabétique, etc.).
- o Souffrir d'une co-morbidité physique ou psychiatrique majeure et suffisamment sévère pour altérer la capacité du sujet à participer à l'étude.
- o être en litige au sujet d'indemnisations de la CSST ou de la SAAQ.

À noter que seront exclus en cours d'étude les sujets qui commencent un nouveau traitement analgésique.

4.4 Déroulement de l'étude et collecte des données

4.4.1 Formation du personnel de recherche et des intervenants. Les assistants de recherche dans chacun des sites participants recevront une formation en bonne et due forme afin d'assurer l'uniformité des procédures utilisées pour le recrutement des sujets et la collecte des données. Il en sera de même pour les intervenants qui animeront les ÉIF et EIL. Ces derniers participeront à une seule et même séance de formation qui se tiendra à l'Université de Sherbrooke et durant laquelle seront revus en détail le contenu de chacune des sessions, les modes d'intervention préconisés et les stratégies à prendre dans les cas problèmes. Toutes les sessions des ÉIF et ÉIL seront filmées afin de documenter l'arrimage inter-sites dans l'application du programme et pour développer un outil vidéo qui servira à la formation des intervenants ultérieurs dans les autres centres de réadaptation du Québec où l'ÉIF et l'ÉIL seront par la suite implantées.

4.4.2 Procédure de recrutement et collecte des données. Le recrutement des sujets se fera à l'aide d'une stratégie non probabiliste où des annonces seront publiées dans les journaux locaux de la région de Sherbrooke et de Rouyn-Noranda (voir l'annonce en **Annexe 1**). Les sujets intéressés à prendre part à l'étude seront invités à communiquer par téléphone avec l'assistante de recherche. Au moment de cet appel, cette dernière effectuera une première entrevue de présélection à l'aide d'un guide d'entrevue de présélection structuré (**Annexe 2**) afin de vérifier si le sujet satisfait les critères de sélection de l'étude. Si tel est le cas, l'assistante de recherche expliquera au sujet les objectifs et le déroulement de l'étude de même que la nature de sa participation. Si le sujet démontre un intérêt, un premier rendez-vous sera fixé au site de l'étude afin de : confirmer, par un médecin, le diagnostic de FM ou de LB non spécifique du sujet ainsi que pour vérifier l'intérêt du sujet à faire partie de la recherche, son intérêt pour apprendre à mieux gérer la douleur (objectif de l'intervention) et la disponibilité du conjoint pour répondre aux différents questionnaires. Si le sujet est sélectionné pour l'étude, il y aura par la suite lecture et signature du formulaire de consentement de la personne ayant une DC et du conjoint (ce dernier pourra être obtenu lors de la première séance de l'ÉI), l'évaluation de base (bilan interactionnel et évaluation physique) de même que l'administration des mesures d'évaluation initiale (T_0) à

l'aide de questionnaires auto-administrés composés d'échelles dûment validées. Une période de 90 minutes devrait suffire pour compléter cette évaluation initiale (T_0) incluant les questionnaires. Après ce premier rendez-vous, les sujets seront randomisés dans le groupe intervention ou dans le groupe contrôle en stratifiant selon l'intensité de la douleur et le sexe. Les sujets du groupe intervention seront invités à la première séance de l'école et les sujets du groupe contrôle seront avisés de leur participation ultérieure à l'intervention.

Les sujets qui auront été assignés au groupe intervention débuteront par la suite le programme de l'ÉIF ou de l'ÉIL qui inclura 9 sessions hebdomadaires étalées sur une période de 11 semaines. Les thématiques abordées et le contenu de chacune des sessions sont décrits au Tableau 1. À la toute fin de la dernière session du programme (T_1), soit 3 mois post-randomisation, à 3 mois post-intervention (T_2), à 6 mois post-intervention (T_3), où il y aura une relance, et à 12 mois post-intervention (T_4), les sujets du groupe intervention se verront administrer sensiblement les mêmes questionnaires que ceux utilisés lors de l'évaluation initiale. La durée totale de l'étude sera donc de 15 mois pour ce groupe. Pour ce qui est des sujets assignés au groupe contrôle, les mêmes mesures seront colligées simultanément. Des mesures immédiates post-intervention seront prises aussi chez les sujets du groupe contrôle une fois qu'ils auront complété l'EIF ou L'EIL. La durée totale de l'étude pour le groupe contrôle sera donc aussi de 15 mois tel qu'illustré à la **Figure 1**.

Figure 1 Déroulement et collecte des données

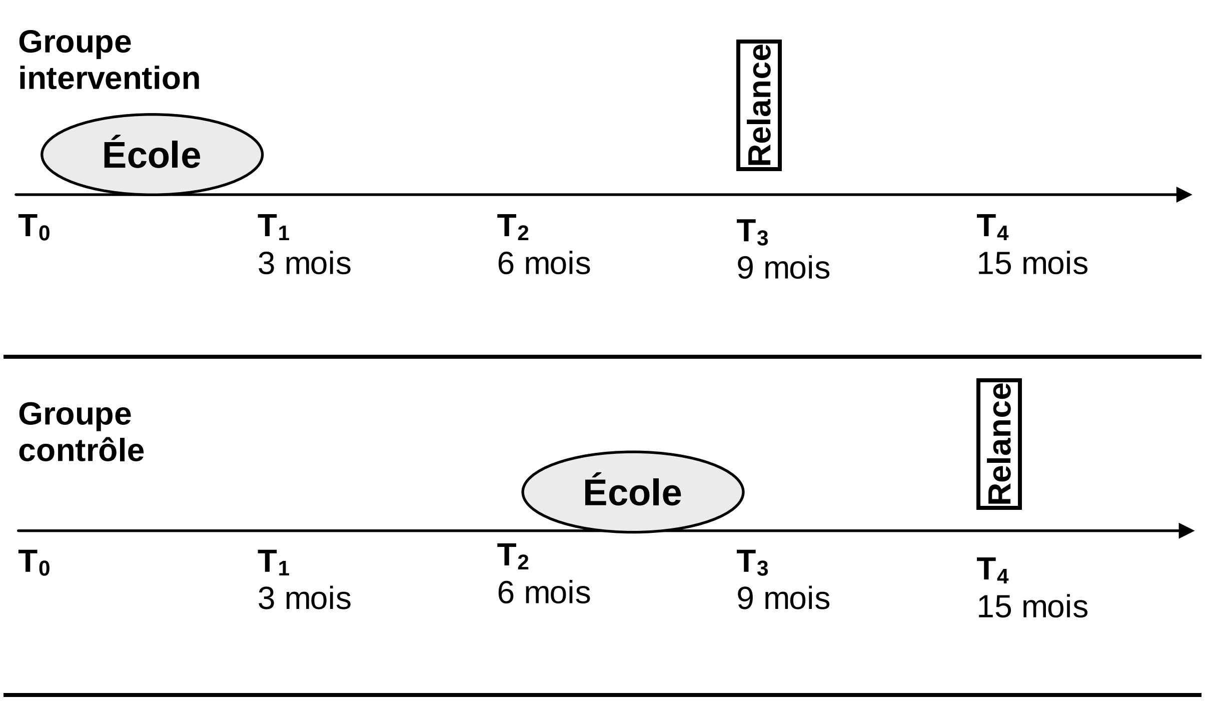

Les mesures seront prises à chacun des temps (T_0 à T_4) auprès des participants et auprès de leur proche et ce, pour les 2 groupes (intervention et contrôle).

À noter que des mesures seront aussi prises auprès des conjoints à tous les temps de mesure à l'aide de questionnaires auto-administrés. Un formulaire de consentement leur sera donc aussi adressé préalablement. La durée prévue pour qu'un conjoint complète les questionnaires est estimé à quarante-cinq minutes.

La majorité des données de l'étude seront recueillies grâce à des questionnaires auto-administrés. Les questionnaires T_0, T_1, et T_3 seront remis aux participants en main propre et seront complétés sur place tandis que les questionnaires du T_2 et T_4 (groupe intervention uniquement) seront envoyés aux participants par la poste. Pour le groupe contrôle, ce ne sont que les questionnaires du T_1 qui seront envoyés par la poste. Ces derniers seront donc invités à compléter ces questionnaires à la maison dans la semaine qui suit et à les retourner par la poste dans l'enveloppe pré-adressée et préaffranchie qui leur aura été fournie. Une semaine après l'envoi postal des questionnaires, l'assistante de recherche appellera chaque sujet afin de lui rappeler l'importance de sa participation à l'étude.

Vu la complexité des mesures spécifiques à l'analyse économique préliminaire, les données sur l'utilisation de ressources de santé et sur la perte de productivité seront recueillies par téléphone par l'assistante de recherche. Des entrevues téléphoniques d'environ 15 minutes seront planifiées dans la même semaine que l'administration des questionnaires auto-administrés (T_0 à T_3 chez le groupe intervention et T_0 à T_2 chez le groupe contrôle).

4.5 Variables et instruments d'évaluation

4.5.1 Variables indépendantes

La présente étude comporte deux variables indépendantes. La première réfère à la condition expérimentale—i.e., intervention EI vs contrôle. La seconde variable indépendante réfère au passage du temps—i.e., avant (T_0) et après l'intervention où des mesures sont effectuées à différents moments pour le groupe intervention (T_1 –post-intervention immédiat, T_2 – 6 mois post-intervention, T_3 - 9 mois post-intervention et T_4 - 15 mois post-intervention). Pour le groupe contrôle, trois mesures seront prises avant l'intervention et deux suivant la fin de l'intervention (**figure 1**).

L'intervention des EI suit le modèle interdisciplinaire de l'École interactionnelle du dos (Charest, *et al*., 1994) présenté précédemment. Tel qu'illustré dans le **Tableau 1**, les ÉIF/ÉIL incluent neuf rencontres. Les premières sont hebdomadaires et étalées sur 5 semaines. Il y a ensuite une semaine de travail autonome entre les séances 5 et 7, suivi de deux semaines travail autonome et finalement la séance 8. La 9e séance correspond à la visite de relance. Les rencontres se font en groupe de huit sujets avec deux intervenants de différentes disciplines professionnelles, pour favoriser l'approche interdisciplinaire, idéalement une intervenante ayant une formation au niveau psychologique (p.ex. : psychologue, infirmière, etc.) et une autre au niveau physique (p.ex. : ergothérapeute, physiothérapeute, etc.). Chaque

rencontre a une durée d'environ deux heures trente incluant une pause interactionnelle de quinze minutes.

Tableau 1 Thématiques et contenus des sessions des Écoles interactionnelles (ÉIF/ ÉIL)

Sessions	Contenus ÉIF/EIL
**SÉANCE 1**<br>**CONTRAT**	**Participant**<br>▪ Présentation de la fibromyalgie ou de la lombalgie.<br>▪ Présentation de chaque étape de l'école.<br>▪ Présentation des membres du groupe (nom, occupation, début symptômes, brève histoire de la maladie).<br>▪ Formulation d'un contrat : (a) établir trois objectifs personnels mesurables à atteindre à la fin de l'école, (b) déterminer le changement minimal acceptable visé à la fin de l'intervention (c) définir le temps consacré aux activités découlant de l'école (45min/j, 6 fois/sem.).<br>▪ Apprentissage de la respiration diaphragmatique (position assise).<br>▪ Tâche : 3 fois par jour faire 3 respirations diaphragmatiques (au minimum).
**SÉANCE 2**<br>**SYMPTÔME**	• Retour sur la rencontre précédente.<br>• Présentation des symptômes de la FM ou de la LB et des quatre symptômes qui seront touchés pendant l'école : la douleur, la fatigue, le sommeil et le stress.<br>• Identification des stratégies des participants qui sont efficaces pour améliorer la qualité du **sommeil**.<br>• Compléter avec les autres stratégies.<br>• Tâche : essayer deux stratégies pour favoriser le sommeil et compléter pendant la semaine le carnet de route (balle pression, marche selon ma capacité 3 fois/sem.).
**SÉANCE 3**<br>**PRÉPARATION PHYSIQUE**	• Retour sur la rencontre précédente.<br>• Rationnel de l'activité physique et son influence sur les symptômes.<br>• Test physique.<br>• Identification des stratégies des participants qui sont efficaces pour la **douleur**.<br>• Compléter avec les autres stratégies.<br>• Tâche : marche selon ma capacité 3 fois par semaine, poursuivre les autres stratégies, pratique routine motrice 6 fois/semaine et compléter le carnet de route pendant la semaine.
**SÉANCE 4**<br>**GESTION DU STRESS**	• Retour sur la rencontre précédente.<br>• Routine motrice<br>• Rationnel de la force mental sur la modulation de la douleur ainsi que sur la gestion du stress.<br>• Pratique d'une technique de relaxation active.<br>• Identification des stratégies des participants qui sont efficaces pour gérer le **stress**.<br>• Compléter avec les autres stratégies.<br>• Tâche : relaxation 3 fois par semaine, poursuivre les autres stratégies et compléter pendant la semaine le carnet de route.
**SÉANCE 5**<br>**GESTION DES CAPACITÉS**<br>**(ÉIF)**	• Retour sur la rencontre précédente.<br>• Pratique de la routine en classe.<br>• Apprendre à : (a) identifier ses limites, (b) respecter ses limites, (c) valoriser ses capacités (principe d'économie d'énergie, conservation de l'énergie, ergonomie).et d) pourquoi et comment bien se nourrir, avec une emphase sur le guide alimentaire canadien.<br>• Identification des stratégies des participants qui sont efficaces pour diminuer la **fatigue (augmentation de l'énergie)**.<br>• Essai de nouveaux aliments santé<br><br>• Compléter avec les autres stratégies.<br>• Tâche : appliquer deux des stratégies de gestion des capacités et manger de façon équilibrée et intégrer deux nouveaux aliments, poursuivre les autres stratégies et compléter pendant la semaine le carnet de route, poursuivre les autres stratégies et

	compléter pendant la semaine le carnet de route.<br>• Pratique d'une technique de relaxation passive.
**ANATOMIE (ÉIL)**	• Retour sur la rencontre précédente.<br>• Comprendre l'anatomie et le fonctionnement de la colonne vertébrale et adapter son environnement.<br>• Pratiques de positions à adopter.<br>• Identification des stratégies de mobilisation qui sont efficaces pour diminuer la **fatigue (augmentation de l'énergie)**..<br>• Compléter avec les autres stratégies.<br>• Tâche : appliquer deux des stratégies de mobilisation iet ntégrer deux nouvelles positions, poursuivre les autres stratégies et compléter pendant la semaine le carnet de route, poursuivre les autres stratégies et compléter pendant la semaine le carnet de route
**PAUSE 1 SEMAINE**	• Travail autonome.
**SÉANCE 6 CHRONICITÉ**	• Retour sur la rencontre précédente.<br>• Pratique de la routine motrice en classe.<br>• Modulation de la douleur, vivre avec la douleur chronique et les conséquences indésirables d'une guérison (stratégie, outil de gestion).<br>• Poursuite des tâches précédentes et compléter pendant la semaine le carnet de route.<br>• Technique de relaxation (*focussing*)
**PAUSE 1 SEMAINE**	• Travail autonome.
**SÉANCE 7 TRAITEMENTS**	• Retour sur la rencontre précédente.<br>• Routine motrice en classe.<br>• Rationnel des traitements pharmacologiques et non pharmacologiques et de la chirurgie (ÉIL seulement).<br>• Poursuite des tâches précédentes et compléter pendant la semaine le carnet de route.
**PAUSE 2 SEMAINES**	• Travail autonome.
**SÉANCE 8 CONDITION INITIALE**	**Participant seulement**<br>• Retour sur la rencontre précédente.<br>• Discussion du cheminement parcouru durant l'école.<br>• Réévaluation du temps à consacrer à l'activité physique, la relaxation et les autres stratégies pendant les semaines à venir.<br>• Diplôme<br>• Poursuite des tâches précédentes et compléter pendant les semaines le carnet de route.
**SÉANCE 9 RELANCE**	• Retour sur les semaines précédentes.<br>• Discussion du cheminement parcouru depuis la fin de l'école.<br>• Discussion des stratégies appliquées et qui fonctionnent.<br>• Ajustement des routines motrices individualisées<br>• Poursuite des tâches précédentes.

À partir de la 4e session, la routine motrice est pratiquée à l'arrivée (15 minutes) et la session se termine par une relaxation (15 minutes).

Les sessions 5 et 6 présentent des sujets différents entre les ÉIF et ÉIL.

4.5.2 Variables dépendantes et instruments de mesure utilisés auprès des participants

Le choix des variables dépendantes et des instruments de mesure utilisés dans la présente étude s'est appuyé sur les récentes recommandations du Groupe IMPPACT (Inititatives on Methods, Measurement, and Pain Assessement in Clinical Trials) (Brislin, 1986; Turk, Dworkin, Allen, Bellamy, Brandenburg, Carr *et al*., 2003; Dworkin, Turk, Farrar, Haythornthwaite, Jensen, Katz *et al.*, 2005). La sélection des instruments de mesure s'est effectuée en tenant compte de la qualité de leurs qualités psychométriques (p.ex., validité, fidélité, sensibilité, etc.), de la disponibilité d'une version française dûment validée et de l'existence de données normatives lorsqu'applicable. La sélection a aussi été guidée par un souci de minimiser le plus possible le fardeau imposé aux participants. Enfin, le choix des mesures s'est appuyé sur l'expérience des responsables du présent projet qui ont utilisé une batterie similaire de questionnaires dans des études antérieures incluant celle de Barcellos de Souza (2007). Pour ce qui est des items sélectionnés pour mesurer les coordonnées démographiques des participants, leur histoire médicale incluant celle de la douleur de même que les traitements pharmacologiques et non pharmacologiques qu'ils utilisent pour contrer la douleur, ils ont été tirés du Registre Québec Douleur créé en 2006, lequel est actuellement employé dans les Centres d'expertise en traitement de la douleur des RUIS du Québec notamment au CHUS.

Le **Tableau 2** reproduit en **Annexe 3** liste chacune des variables mesurées dans la présente étude auprès des participants FM ou LB et à quels moments elles le seront. Pour ce qui est des variables qui seront mesurées au moment de l'évaluation initiale, elles peuvent être regroupées et résumées de la façon suivante :

- ***Caractéristiques de la douleur*** incluant, entre autres, son intensité qui sera mesurée à l'aide d'une échelle numérique de 0 à 10 où 0 = aucune douleur et 10 = pire douleur possible (Jensen et Karoly, 2001) de même que son impact dans diverses sphères de la vie quotidienne (Items d'interférence du Questionnaire concis sur la douleur modifié (*Brief Pain Inventory*) (Cleeland et Ryan, 1994 ; Larue, Colleau, Brasseur et Cleeland, 1995 ; Tyler *et al*., 2002).

- ***Qualité du sommeil*** mesurée à l'aide du *Chronic Pain Sleep inventory (Kosinki, Janagap, Gajria, Schein (2007)*.

- ***Traitements pharmacologiques et non pharmacologiques*** utilisés présentement pour contrer la douleur et autres désordres médicaux.

- ***Consommation de tabac, alcool, drogues***

- ***Stratégies d'ajustement face à la douleur***
  - o ***Tendance à la dramatisation*** mesurée à l'aide de l'Échelle de dramatisation face à la douleur (*Pain Catastrophizing Scale*) (Sullivan, Bishop et Pivik, 1995 ; French, Vigneau, French, Cyr et Evans, 2004).
  - o ***Questionnaire sur les stratégies d'ajustement face à la douleur*** (CSQ-F) (*Coping strategies Questionnaire* Rosenstiel et Keefe, 1983, Irachbal *et al*., 2002).

- ***Niveau de dépression*** évalué à l'aide de l'Inventaire de dépression de Beck – V1 (*Beck Depression Inventory)* (Beck, Ward, Mendelson, Mock et Erbaugh, 1961 ; Gauthier, Thériault, Morin et Lawson, 1982).

- ***Qualité de vie reliée à la santé*** (*SF-12v2)* (Ware, Kosinski et Keller, 1996).

- ***Attentes du patient face à l'intervention*** EI —i.e., changement attendu au niveau de la douleur, du fonctionnement quotidien et de la qualité de vie *(Patient Global Impression of Change)* – échelle catégorielle allant de «considérablement détériorée» à «considérablement améliorée» (Dworkin, Nagasako, Hetzel et Farrar, 2001 ; Farrar, 2003). Une échelle de degré de soulagement (0%=aucun soulagement, 100%=soulagement complet) (Haythornthwaite et Fauerbach, 2001 ; Jensen, 2003) sera également employée pour mesurer les attentes du patient en termes de soulagement.

- ***Coûts associés à la douleur*** (utilisation de ressources de santé et perte de productivité) Voir 4.5.4 pour plus de détails sur ce questionnaire.

- ***Caractéristiques sociodémographiques*** (sexe, année de naissance, ethnicité, langue première, scolarité, condition de vie actuelle, statut civil, statut d'emploi, etc.).

Participants EIF

- ***Impact de la fibromyalgie*** lequel sera mesuré à l'aide du Questionnaire d'impact de la fibromyalgie *(Fibromyalgia Impact Questionnaire)* (Burckhardt, Clark et Bennett, 1991 ; Perrot *et al*., 2003).

Participant EIL

- ***Incapacités engendrées par la lombalgie*** qui seront évaluées à l'aide de l'Échelle d'incapacité du dos du Québec (Kopec *et al*., 1996)

Toutes les mesures décrites ci-haut seront administrées aux T_0, T_1, T_2 T_3 et T_4 à l'exception de celles ayant trait aux attentes du participant face à l'intervention EI qui ne seront administrées qu'au T_0. Ces dernières sont remplacées par des questions ayant trait à l'impression globale de changement tel que perçu par le sujet en regard de sa douleur, de son statut fonctionnel et de sa qualité de vie et

ce, à l'aide d'une échelle catégorielle allant de «considérablement détérioré» à «considérablement amélioré» (*Patient Global Impression of Change)* (Dworkin, Nagasako, Hetzel et Farrar, 2001 ; Farrar, 2003). L'échelle de degré de soulagement (0%=aucun soulagement, 100%=soulagement complet) (Haythornthwaite et Fauerbach, 2001 ; Jensen, 2003) sera également administrée aux mêmes temps. Le patient devra enfin coter son degré de satisfaction face à l'École interactionnelle à l'aide d'une échelle de satisfaction en six points allant de « très insatisfait » à « très satisfait » (Haythornthwaite et Fauerbach, 2001). En ce qui a trait aux informations sociodémographiques, ne seront répétées que les questions ayant trait au statut d'emploi et les sources de revenu.

Les questionnaires et instruments de mesures de cette section sont présentés à l'**Annexe 4**.

4.5.3 Variables dépendantes et instruments de mesure utilisés auprès des conjoints des participants. (voir **Tableau 3 Annexe 5**)

- ***Qualité de vie reliée à la santé*** (*SF-12v2)* (Ware, Kosinski et Keller, 1996).

- Anxiété (*STAI)* STAI-S ou STAI-T (Spielberger *et al*., 1983; Spielberger *et al*., 1988; Gauthier et Bouchard, 1993; Spielberger *et al*., 2008)

- *Inventaire de dépression de Beck-V1* (Beck *et al*., 1961; Bourque et Beaudette, 1982, Beck, Epstein, Brown et Steer, 1988; Beck *et al*. 1996;).

- **Satisfaction de la relation avec le conjoint**

Relation avec le conjoint
Très satisfaisante, plutôt satisfaisante, plutôt insatisfaisante, très satisfaisante

- Questionnaire d'activités et d'attitudes à l'égard de la sexualité Beaudoin, Carbonneau, Godbout, Bouchard et Sabourin (2007).

- **Santé perçue** (Santé Québec, 1995)

Question évaluant la santé perçue par le conjoint en regard de sa propre santé comparativement à d'autres personnes de son âge.

- **Échelle d'efficacité personnelle** (Bandura, 1977; Hébert *et al*., 2004).

Questionnaire global portant sur l'efficacité personnelle perçue adapté au rôle de soutien. L'efficacité personnelle est la conviction que l'on peut produire un résultat positif et la conviction que l'on peut faire un comportement qui réfère, dans la présente étude, à la capacité d'assumer son rôle de soutien auprès de son conjoint.

- ***Attentes du conjoint face à l'intervention*** EI —i.e., changement attendu au niveau de la douleur, du fonctionnement quotidien et de la qualité de vie *(Patient Global Impression of Change)* – échelle catégorielle allant de «considérablement détériorée» à «considérablement améliorée» (Dworkin, Nagasako, Hetzel et Farrar, 2001 ; Farrar, 2003). Une échelle de degré de soulagement perçu cgez le participant à l'ÉI (0%=aucun soulagement, 100%=soulagement complet) (Haythornthwaite et Fauerbach, 2001 ; Jensen, 2003).

- **Caractéristiques sociodémographiques et personnelles** (sexe, année de naissance, ethnicité, langue première, scolarité, condition de vie actuelle, statut civil, statut d'emploi, revenu familial, satisfaction en regard du revenu, principale source de revenu, type de lien avec la personne FM ou LB, cohabitation ou non avec la personne FM ou LB, durée de l'aide apportée).

Les questionnaires et instruments de mesures de cette section sont présentés à l'**Annexe 6**.

4.5.4 Variables et mesures spécifiques à l'analyse économique.

L'évaluation économique préliminaire réalisée dans le cadre de cette étude prendra en considération tous les coûts directs (utilisation de ressources de santé) et les coûts en perte de productivité (anciennement désignés coûts indirects) associés à la douleur chronique. Les informations nécessaires à l'estimation de ces coûts seront répertoriées grâce au questionnaire sur l'utilisation de ressources de santé et la perte de productivité (**Annexe 7**) qui sera administré au téléphone par l'assistante de recherche.

Dans le groupe d'intervention (EIF/EIL) et dans le groupe contrôle, les coûts encourus durant les 3 derniers mois seront mesurés de façon rétrospective lors de l'entrevue initiale (T_0), ainsi qu'au T_1 et au T_2, afin de pouvoir établir une comparaison entre le groupe d'intervention (EIF/EIL) et le groupe contrôle. Dans le groupe d'intervention (EIF/EIL) seulement, les coûts encourus durant les 3 derniers mois seront également mesurés au T_3. Bien qu'aucune comparaison ne sera possible avec le groupe contrôle, les coûts mesurés au T_3 pourront êtres comparés aux coûts mesurés au T_0 dans ce même groupe.

Coûts directs
Afin d'obtenir une mesure des coûts directs pour toutes les ressources de santé utilisées à cause de la douleur, des informations seront répertoriées sur le nombre et la durée des hospitalisations et des visites à l'urgence, le nombre de visites chez un médecin omnipraticien, un médecin spécialiste ou tout autre professionnel de la santé, l'achat de médicaments, de produits naturels ou de fournitures et l'utilisation d'aide domestique rémunérée. La valorisation financière des ressources utilisées par chaque participant pourra ensuite être obtenue en multipliant la quantité de chacune des ressources par leur coût unitaire. Les coûts unitaires pourront être obtenus grâce au Rapport de l'Association Québécoise d'Établissements de Santé

et de Services sociaux, aux informations recueillies auprès de la Régie de l’Assurance Maladie du Québec et en s’informant auprès du patient.

Perte de productivité
Puisqu’un problème de santé peut entraîner une diminution du temps pendant lequel un individu est productif, il est intéressant de pouvoir estimer la valeur monétaire de la perte ou du gain de productivité découlant d’une intervention. (Rothermich et Pathak, 1999). Malgré les nombreuses controverses entourant l’estimation des coûts de la perte de productivité, (Peeples *et al.*, 1997; Rothermich et Pathak, 1999; Sach et Whynes, 2003) la méthode du capital humain (Koopmanschap et Rutten, 1996) demeure la plus utilisée (Liljas, 1998) et constitue donc la méthode qui sera adoptée dans la présente étude. Chez les travailleurs, les coûts en perte de productivité seront donc obtenus en multipliant l’absentéisme (nombre de jours d’absence au travail en raison de la douleur) par le salaire journalier des sujets (Berger et *al*., 2001). Chez les participants sans travail rémunéré, les coûts en perte de productivité seront obtenus grâce à l’approche du coût de remplacement, qui correspond à ce que couteraient les activités de la femme/homme "au foyer" aux prix du marché. (Drummond et *al*. 1997) Par conséquent, le nombre de jours de travail au foyer perdus (à cause de la douleur) sera multiplié par l’estimation du coût de remplacement journalier du travail non rémunéré selon l'âge et le sexe. Les coûts de remplacement seront obtenus grâce aux données les plus récentes de Statistique Canada.

4.5.5 Suivi de l’implantation
Afin d’éviter une erreur de type 3, soit l’attribution d’un manque d’effet des EI alors que leur implantation est incomplète, un suivi sera réalisé après chaque séances pour assurer le suivi (*monitoring*) de l’intervention (Rossi et Freeman, 1993). Cette démarche est d’autant plus importante car les EI seront implantées sur deux sites. Pour évaluer l’implantation des EI, une étude de l’adéquation du a/n de la rationalité structurelle (degré d’intégration des ressources et des outils de même que le respect du calendrier prévu), et fonctionnelle (niveau de concordance des contenus et des activités des EI avec ce qui était prévu) de même que la participation de la population cible (degré de mise en œuvre : nombre d’abandons, nombre de rencontres auxquelles assisteront les participants, nombre de stratégies intégrées). Les écarts observés entre ce qui avait été prévu au plan initial et ce qui est réalisé seront documentés (Huberman et Miles, 1991). Les interventions visant à combler ces écarts seront proposées de même que l’évaluation de leur application. Cette analyse d’implantation sera la pierre angulaire du déploiement des EI lors de la Phase 2 du projet ACCORD.

4.6 Analyses statistiques

4.6.1 Estimation de la taille échantillonnale
Trente-deux patients FM seront recrutés à Sherbrooke (16 dans le groupe expérimental et 16 dans le groupe contrôle) et 32 patients FM seront recrutés à Rouyn-Noranda (16 dans le groupe expérimental et 16 dans le groupe contrôle). Cette taille de l'échantillon est basée d’une part sur les résultats de l'ÉIF

récemment publiés par Barcellos de Souza *et al*. (2007), où les auteurs rapportent des effets statistiquement significatifs, et d'autre part des diminutions d'intensité de douleur de l'ordre de 30%, lesquelles ont été reconnues dans différentes études comme étant cliniquement significative (Farrar *et al*. 2001). Aussi, dans cette étude, le taux d'attrition est nul.

Afin de confirmer objectivement la taille de notre échantillon, nous avons utilisé la formule suivante (Cohen, 1988):
$n=2*(Z_\alpha+Z_\beta)^2 X \sigma^2/\delta^2$ où $\alpha=.05$, $\beta=.30$ et σ^2/δ^2 (i.e., l'inverse de la taille de l'effet).

Assumant une taille de l'effet modeste (Cohen's d=0.7, ce qui est conservateur basé sur l'étude de Barcellos de Souza *et al*., 2007), et utilisant la formule décrite ci-haut, nous obtenons un échantillon d'approximativement 16 sujets FM par groupe. Donc, pour Sherbrooke, nous aurons besoins de 32 sujets FM. De même, nous aurons besoins de 32 sujets FM pour Rouyn-Noranda.

Puisque nous utilisons sensiblement le même type d'intervention (École Interactionnelle), les paramètres utilisés pour calculer la taille de l'échantillon chez les FM devraient donc être transposables pour le calcul de l'échantillon chez les LB. Nous devrons donc recruter 32 LB à Sherbrooke (16 dans le groupe expérimental et 16 dans le groupe contrôle) et 32 LB à Rouyn-Noranda (16 dans le groupe expérimental et 16 dans le groupe contrôle).

4.6.2 Analyses des données

Pour chaque EI, des statistiques descriptives (mesures de tendance centrale et de dispersion, tableaux de fréquence) seront d'abord calculées pour décrire les caractéristiques démographiques (p.ex. : âge, sexe) et cliniques (p.ex. : durée de la douleur) des groupes à l'étude. Les caractéristiques des sujets des groupes expérimentaux et contrôles seront ensuite comparés en utilisant un test t pour groupes indépendants ou un test du chi-deux selon que les variables sont de nature continue ou catégorielle. Les résultats obtenus aux différents temps de l'étude (T_0 , T_1, T_2) dans les deux groupes (intervention vs contrôle) sur la variable primaire de sévérité de douleur—i.e., intensité moyenne au cours des 7 derniers jours seront comparés à l'aide à l'aide de statistiques paramétriques (ANOVA ou ANCOVA à 2 facteurs avec mesures répétées) ou non paramétriques selon la nature de la distribution de la variable. Les proportions de patients qui auront vu leur niveau d'intensité de douleur baisser dans le temps d'au moins 30% seront également calculées et comparées dans les 2 groupes. En ce qui concerne les autres variables incluses dans les objectives secondaires (impact fonctionnel de la douleur, dramatisation, qualité du sommeil, bien-être psychologique, qualité de vie reliée à la santé (participant); stress, dépression, sentiment d'efficacité, santé perçue (conjoint)), coûts reliés à l'utilisation de ressources de santé et à la perte de productivité), les groupes seront comparés aux différents temps à l'aide des mêmes tests que ceux décrits précédemment. Toutes les analyses statistiques seront réalisées avec le logiciel SAS et le seuil de signification statistique sera fixée à $p < .05$. Enfin, une analyse coût-efficacité sera réalisée en comparant les coûts aux T_0, T_1 T_2 chez des groupes interventions et groupes contrôles à l'aide du

même type de tests que ceux décrits ci-haut (ANOVA ou ANCOVA avec mesures répétées).

4.7 Considérations éthiques

Lors du premier contact téléphonique, une explication détaillée de l'étude sera donnée au participant éventuel et l'assistante de recherche répondra à toutes questions relatives à l'étude. De plus, lors de la première rencontre, un des investigateurs ou l'assistante de recherche répondront à toutes autres questions ayant pu survenir entretemps. Un formulaire de consentement sera remis aux participants (**Annexe 8**) et à un conjoint (**Annexe 9**) pour lecture et signature. Une copie leur sera remise. Tous les participants de l'étude seront libres de se retirer en tout temps. Les renseignements recueillis demeureront strictement confidentiels et seront gardés sous clés dans une filière à la FMSS/UQAT. Au moment de l'entrée de données, chaque participant se verra assigner un numéro de code. La base des données sera sécurisée et seule la chercheure sera en mesure d'associer les codes aux participants. Les données seront détruites 5 ans après la fin de l'étude. Le protocole sera soumis au Comité d'éthique de la recherche (CER) sur l'humain du CHUS et à celui de l'UQAT pour approbation. Le Programme ACCORD dont fait partie le présent projet a été révisé par un Comité de pairs des IRSC et bénéficie d'une subvention des IRSC (**Annexe 10**).

5. RETOMBEES ET IMPACTS DU PROJET

Les Écoles interactionnelles (EI) proposées ici contribueront, nous l'espérons, à combler les écarts entre les besoins des personnes fibromyalgiques (FM) et lombalgiques (LB) et le peu de services qui leur sont offerts, dans le contexte de conjoncture économique actuelle dans le système de santé québécois. La participation à ces EI devrait permettre d'améliorer la qualité de vie des personnes au prise avec des problématiques de FM ou de LB en les outillant à mieux gérer leur douleur pour en diminuer l'intensité. Parmi les aspects novateurs de ces EI, l'évaluation de l'impact sur le conjoint devrait contribuer à mieux saisir l'effet de l'intervention. Le second aspect novateur réside dans le fait que les EI proposées reposent sur un modèle facilement exportable dans d'autres Centres de réadaptation compte tenu de leur structure de fonctionnement et des ressources relativement limitées et accessibles qu'elles emploient. Le troisième aspect novateur réfère au modèle qui sera créé et qui servira d'outil de référence pour le déploiement ultérieur de ces EI dans une vingtaine de centres de réadaptation au Québec ce qui représente une ressource inestimable pour ce nombre croissant de personnes affligées par la douleur chronique et leur famille. En dernier lieu, à ce jour, aucune étude n'a évalué l'impact économique d'Écoles Interactionnelles dans le traitement de la douleur chronique telle que la lombalgie ou la fibromyalgie. Le présent projet permettra d'obtenir des données préliminaires à cet effet, lesquelles pourront appuyer une demande de subvention pour une étude sur une plus grande échelle. Puisque la douleur chronique compte parmi les conditions de santé les plus incapacitantes et les plus coûteuses, les résultats de cette étude sont

essentiels en vue d'éclairer le choix quant aux interventions de santé à privilégier dans un contexte de ressources limitées pour la prise en charge de cette condition de santé.

6. ÉCHÉANCIER

Activités	2008	2009	2010	2011
**Rencontres des membres de l'équipe de recherche et partenaires**	**Novembre -**			
**Soumission aux CER (Sherbrooke/UQAT)**		**Février**		
**Formation des intervenants**		**Avril/mai**		
**Recrutement**		**Juillet/août**		
**Expérimentation des 2 EI à Sherbrooke et à l'UQAT (Groupes Expérimental)**		**Sept./déc.**		
**Expérimentation des EI aux groupes contrôles (Groupes Contrôle)**			**Mars/juin**	
**Relance des 2 EI (Groupes E)**			**Juin**	
**Relance des 2 EI (Groupes C)**			**Décembre**	
**Analyse des données**				**Janvier/mai**
**Diffusion et publications**				**Septembre**

7. RÉFÉRENCES

Affleck G, Urrows S, Tennen H, Higgins, P, Abeles, M. (1996). Sequential daily relations of sleep, pain intensity and attention to pain among women with fibromyalgia. *Pain*, 68, 363-368.

American Pain Society (2003). Chronic pain in America: Roadblocks to relief.

Anderson, R.M., Funnell, M.M. (2005). Patient empowerment: reflections on the challenge of fostering the adoption of a new paradigm. *Patient Education and Counseling. 57*, 153-157.

Arnow, B.A. Hunkeler, E.M., Blasey, C.M., Lee, J., Constantino, M.J., Fireman, B., Kraemer, H.C., Dea, R., Robinson, R., Hayward, C. (2006). Comorbid Depression, Chronic Pain, and Disability in Primary Care. *Psychosomatic Medicine*, 68, 262–268.

Arsenault, P., Marchand, S. (2007). Synthèse des mécanismes impliqués dans un syndrome douloureux complexe: la fibromyalgie. *Douleur et Analgésie, 20*(4), 200-212.

Asay, T. P., Lambert, M. J. (1999). The empirical case for the common factors in therapy: Quantitative findings. *In* M. A. Hubble, B. L. Duncan, S. D. Miller (Eds.), *The heart and soul of change: What works in therapy* (p. 33-56). Washington, D. C.: American Psychological Association.

Ashburn, M.A., Staats P.S. (1999). Management of chronic pain. *Lancet*, *353*,1865-1869.

Association québécoise de la douleur chronique. (2005). *Site de l'Association québécoise de douleur* chronique. Statistiques. Site téléaccessible à l'adresse <http://www.douleurchronique.org./html/content.asp?node>. Consulté le 5 février 2008.

Bandura, A. (1977). Self-efficacy : toward a unifying theory of the behavioral change. *Psychological Review*, 84, 191-215.

Barcellos de Souza, J. (2007). École Interactionnelle de Fibromyalgie: un traitement multidisciplinaire pour une population hétérogène. Thèse. Université de Sherbrooke, Faculté de médecine et des sciences de la santé.

Barcellos de Souza, J., Charest, J., Marchand, S. (2007). École interactionnelle de fibromyalgie: description et évaluation. Douleur et Analgésie, *20*(4), 213-218.

Beaudoin, A-S., Carbonneau, N., Godbout, N., Bouchard, S. et Sabourin, S. (2007). Validation préliminaire du Questionnaire d'activités et d'attitudes à l'égard de la sexualité. Canadian Journal of Behavioural Science.

Beck, A. T., Epstein, N., Brown, G., Steer, R. A. (1988). An inventory for measuring clinical anxiety: psychometric properties. *Journal of Consulting and Clinical Psychology*. *56*, 893-897.
Beck, A.T., Steer, R.A., Brown, G.K. (1996). *Manual for the Beck Depression Inventory BDI-II*. San Antonio, Tx.

Beck, A.T., Ward, C.H., Mendelson, M., Mock, J., Erbaugh, J. (1961). An inventory for measuring depression. *Archives of General Psychiatry*, *4*, 561-571.

Bennett, R., Burckhardt, C.S., Clark, S., O'Reilly, C.A., Campbell, S.M. (1996). Group treatment of fibromyalgia: a 6 month outpatients program. *Journal of Rheumatology*, *23*, 521-528.

Bennett, R.M. (2003). Textbook of pain. *In* Wall, P.D., Melzack, R., *Fibromyalgia.* Churchill Livingstone, Toronto (4nd éd.).

Berger, M. L., Murray, J. F., Xu, J., Pauly, M. (2001). Alternative valuations of work loss and productivity. *Journal of Occupational and Environmental Medicine*, *43* (1), 18-24.

Berman, B.M., Swyers, J.P. (1999). Complementary medicine treatments for fibromyalgia syndrome. *Baillière's Clinical Rheumatology*, *13*(3), 487-492.

Besson, J.M. (1992). *La douleur*. Paris: Éditions Odile Jacob.

Blais, F.C., Gendron, L., Mimeault, V., Morin, C.M. (1997). Évaluation de l'insomnie: validation de trois questionnaires. *L'Encéphale, 23*, 447-453.

Block, S.R. (1999). On the nature of Rheumatism. *Arthritis Care and Research, 12*(2), 129-138.

Blyth, F.M., March, L.M., Brnabic, A.J., Jorm, L.R., Williamson, M., Cousins. M.J. (2001). Chronic pain in Australia: a prevalence study. *Pain*, 89, 127-134.

Bodenheimer, T., Lorig, K., Holman, H., Grumbach, K. (2002). Patient self-management of chronic disease in primary care. *JAMA*, *288,* 2469-2475.

Boulanger, A., Clark, A.J., Squire, P., Cui, E., Horbay, G. (2007). Chronic pain in Canada: have we improved our management of chronic noncancer pain? *Pain Res Manag*, 12, 39-47.

Bourque, P., Beaudette, D. (1982). Étude psychométrique du questionnaire de depression de Beck auprès d'un échantillon d'étudiants universitaires francophones. *Revue canadienne des sciences du comportement*, 14, 211-218.

Branco, J., Atalaia, A., Paiva, T. (1994). Sleep Cycles and alpha-delta sleep in fibromyalgia syndrome. *Journal of Rheumatology, 21*, 1113-1117.

Breivik, H., Collett, B., Ventafridda, V., Cohen, R., Gallacher, D. (2006). Survey of chronic pain in Europe: prevalence, impact on daily life, and treatment. *European Journal of Pain*, *10*, 287-333.

Brislin, R.W. (1986). The wording and translation of reseach instruments, *In* W.J.Lonner, J.W.Berry, editors. *Field methods in cross-cultural research*. Beverly Hills: Sage, 137-164.

Burckhardt, C.S., Clarck, S.R., Bennett, R.M. (1991). The fibromyalgia Impact Questionnaire. Development and validation. *Journal of Rheumatology, 18*, 728-733.
Buysse, D.J., Reynolds, C.F., Monk, T.H., Berman, S.R., Kupfer, D.J. (1989). The Pittsburgh Sleep Quality Index: a new instrument for psychiatric practice and research. *Psychiatry Res., 28*, 193-213.

Charest, J. (1996). Thérapie stratégique : fondements, techniques et application cliniques. *Revue québécoise de psychologie*, *17*(3), 40-73.

Charest, J. (1998). Démarche en thérapie brève deux applications cliniques. *Revue québécoise de psychologie*, *19*(3), 5-27.

Charest, J., Chenard, J.R., Lavignolle, B., Marchand, S. (1996). *Lombalgie : École interactionnelle du dos*, Paris : Masson.

Charest, J., Lavignolle, B., Chenard, J.R., Provencher, M., Marchand, S. (1994). École interactionnelle du dos. *Rhumatologie, 48*(8), 221-237.

Chatain, M., Escourrou, A., Felez, A., Prat, C., Roques, C.F. (1998). École du dos de Toulouse-Rangueil : bilan à 6 ans. Perspective. *Annales de Réadaptation et Médecine Physique, 41*(6), 381.

Chenard, J.R., Lavignolle, B., Charest, J. (1991). *Lombalgie : dix étapes sur les chemins de la guérison*. Paris, Mansson.

Chou, R. (2007). Diagnosis and Treatment of Low Back Pain: A Joint Clinical Practice. Guideline from the American College of Physicians and the American Pain Society. *Annals of Internal Medicine, 147*(7), 478-491.

Clauw, D.J., Crofford, L.J. (2003). Chronic widespread pain and fibromyalgia. What we know, and what we need to know. *Best Practice and Research, 17*(4), 685-701.

Cleeland, C.S., Ryan, K.M. (1994). Pain assessment: global use of the Brief Pain Inventory. *Annals of the Academy of Medicine of Singapore, 23*, 129-138.

Cohen, J. (1988). *Statistical Power Analysis for the Behavioural Sciences* New York: Academic Press (2nd ed.).

Craig, K.D. (2003). Text book of pain. *In* Wall, P.D., Melzack, R., *Emotions and psychobiology* (p.331-344). Churchill Livingstone, Toronto (4nd ed.).

Dauvilliers, Y., Touchon, J. (2001). Le sommeil du fibromyalgique : revue des données cliniques et polygraphiques. *Neurophysiol Clin, 31*, 18-33.

Diabète Québec. Diabète Québec. 2007.

Dionne, C., Bourbonnais, R., Frémont, P., Rossignol, M., Stock, S. (2004). *Le pronostic occupationnel des travailleurs aux prises avec des affections vertébrales*. Études et recherches / Rapport R-356, Montréal, IRSST.

Drummond MF, O'Brien BJ, Stoddart GL, Torrance GW. (1997) Methods for the Economic Evaluation of Health Care Programmes. Oxford: Oxford University Press.

Dworkin, R.H., Nagasako, E.M., Hetzel, R.D., Farrar, J.T. (2001). Handbook of Pain Assessment, *In*: Turk D.C., Melzack R, *Assessment of pain and pain-related quality of life in clinical trials* (p.659-692). New York: Guilford Press (2nd ed.).

Dworkin, R.H., Turk, D.C., Farrar, J.T., Haythornthwaite, J.A., Jensen, M.P., Katz, N.P., Kerns, R.D., Stucki, G., Allen, R.R., Bellamy, N., Carr, D.B., Chandler, J., Cowan, P., Dionne, R., Galer, B.S., Hertz, S., Jadad, A.R., Kramer, L.D., Manning, D.C., Martin, S., McCormick, C.G., McDermott, M.P., McGrath, P., Quessy, S., Rappaport, B.A., Robbins, W., Robinson, J.P., Rothman, M., Royal, M.A., Simon, L., Stauffer, J.W., Stein, W., Tollett, J., Wernicke, J., Witter, J. (2005). Core outcome measures for chronic pain clinical trials: IMMPACT recommendations. *Pain, 113*, 9-19.

Farrar, J. T. (2003). The global assessment of pain and related symptoms. Presented at the second meeting of the Initiative on Methods, Measurement, and Pain Assessment in Clinical Trials. *Site web de IMMPACT-II*. Téléaccessible à l'adresse <www.immpact.org/meetings.html>. Consulté le 5 févirer 2009.

Farrar, J.T., Young, J.P. Jr., LaMoreaux, L., Werth, J.L., Poole, R.M. (2001). Clinical importance of changes in chronic pain intensity measured on an 11-point numerical pain rating scale, 94, 149-58.

Foerster, H.V. (1988). La construction de la réalité. *In* P. Watzlawik, *L'intervention de la réalité* (p.45-69). Paris : Éditions du Seuil.

Gatchel, R.J., Okifuji, A. (2006). Evidence-based scientific data documenting the treatment and cost-effectiveness of comprehensive pain programs for chronic nonmalignant pain. *Journal of Pain, 7,* 779-793.

Gatchel, R.J., Turk, D. (1999). *Psychosocial Factors in Pain: Critical Perspectives*. New York: The Guilford Press.

Gauthier, J., Bouchard, S. (1993). Adaptation canadienne-française de la forme révisée du "State-Trait Anxiety Inventory" de Spielberger. *Revue canadienne des sciences du comportement, 25*, 559-578.

Gauthier, J., Thériault, F., Morin, C., Lawson, J. S. (1982). Adaptation française d'une mesure d'auto-évaluation de l'intensité de la dépression. *Revue québécoise de psychologie, 3,* 1-15.

Gouvernement du Québec. (2006). *Portrait de santé du Québec et de ses regions 2006: les analyses – deuxième rapport national sur l'état de la population du Québec*. Publications du Québec. Québec, Gouvernement du Québec.

Gureje, O., Von Korff, M., Simon, G.E., Gater, R. (1998). Persistent pain and well-being: a World Health Organization study in primary care. *JAMA, 280,* 147-151.

Hadhazy, V., Ezzo, J., Creamer, P., Berman, B. (2000). Mind-body therapies for the treatment of fibromyalgia. A systematic rewiew. *The journal of Rheumatology, 27*(12), 2911-2918.

Haythornthwaite, J.A., Fauerbach, J.A. (2001). Handbook of Pain Assessment, *In*: Turk D.C., Melzack R, *Assessment of acute pain, pain relief and patient satisfaction* (p. 417-430). New York: Guilford Press. (2nd ed.).

Hébert, R., Lévesque, L., Lavoie, J.-P., Vézina, J., Gendron, C., Préville, M., Ducharme, F., Voyer, L. (2004). *Évaluation d'un programme psycho-éducatif de groupe auprès d'aidants de personnes atteintes de démence vivant à domicile*. Rapport de recherché. Centre de recherche sur le viellissement. Institut universitaire de gériatrie de Sherbrooke.

Hirsch-Herpers, B. (2001). La fibromyalgie: symptômes, diagnostic, traitement et recherche. (Trad. par Barbara Hirsch-Herpers). (Révisé par Jean-Charles Boulange-Weill). National fibromyalgia Partnership. Paris .France.

Hodselmans, A.P., Jaegers, S.M., Göeken, L.N. (2001). Short-Term outcome of a back school program for chronic low back pain. *Archives of Physical Medecine and Rehabilitation, 82,* 1099-1105.

Houvenagel, E. (2003). Physiopathologie de la douleur de la fibromyalgie*. Revue du rhumatisme, 70,* 341-320.

Huberman, A.M., Miles, M.B. (1991). *Analyse des données qualitatives.* Belgique : de Boeck.

Irachabal, S., Koleckb, M., Rascleb, N., Bruchon-Schweitzerb, M. (2002). Stratégies de coping des patients douloureux : adaptation française du *coping* strategies questionnaire (CSQ-F). *L'Encéphale, 34*, 47-53.

Jensen, M. P. (2003). The validity and reliability of pain measures for use in clinical trials in adults. Presented at the second meeting of the Initiative on Methods, Measurement, and Pain Assessment in Clinical Trials. *Site web de IMMPACT-II*. Téléaccessible à l'adresse <www.immpact.org/meetings.html>. Consulté le 5 févirer 2009.

Jensen, M.P., Karoly, P. (2001). Handbook of pain assessment, *In* Turk D.C., Melzack R., *Self-report scales and procedures for assessing pain in adults* (p.15-34). New York: Guilford Press (2nd ed.).

Kerns, R.D. (1999). Psychosocial Factors in Pain: Critical Perspectives. *In* Gatchel, R.J., Turk, D. *Family therapy for adults with chronic pain* (p.445-456). New York: The Guilford Press.
Koopmanschap, M. A., Rutten, F.F. (1996). A practical guide for calculating indirect costs of disease. *Pharmacoeconomics, 10*(5), 460-466.

Kopec, J.A., Esdaile, J.M., Abrahamowicz, M., Abenhaim, L., Wood-Dauphinee, S., Lamping, D., Williams, J.I. (1996). The Quebec Back Pain Disability Scale. Conceptualization and development. *J. Clin. Epidemiol*., 49, 151-161.

Kopec, J.A., Sayre, E.C., Esdaile, J.M. (2003). Predictors of back pain in a general population cohort. *Spine, 29*, 70-77.

Kosinki, M., Janagap, C.C., Gajria, K., Schein, J. Psychometric testing and validation of the Chronic Pain Sleep Inventory, Clin Ther 2007, 29 supp : 2562-2577.

Kumpusalo, E., Mantyselka, P., Takala, J. (2000). Chronic pain in primary care. *Family Practice, 17*, 352.

Krsnich-Shriwise, S. (1997). Fibromyalgia syndrome: an overview. *Physical Therapy, 77*(1), 68-75.

Larue, F., Colleau, S.M., Brasseur, L., Cleeland, C.S. (1995). Multicentre study of cancer pain and its treatment in France. *BMJ, 310*, 1034-1037.

Lautenbacher, S., Rollman, G.B. (1997). Possible deficiencies of pain modulation in fibromyalgia. *Clinical Journal of Pain, 13* (3), 189-196.

Lefort, S.M., Gray-Donald, K., Rowat, K.M., Jeans, M.E. (1998). Randomized controlled trial of a community-based psychoeducation program for the self-management of chronic pain. *Pain, 74*, 297-306.

Lemstra, M., et Olszynski, W.P. (2005). The effectiveness of multidisciplinary rehabilitation in the treatment of fibromyalgia: a randomized controlled trial. *The Clinical journal of pain, 21*(2),166-174.

Liljas, B. (1998). How to calculate indirect costs in economic evaluations. *Pharmacoeconomics, 13*(1), 1-7.

Loeser, J.D. (1999). Economic implications of pain management. *Acta Anaesthesiol.Scand, 43*, 957-959.

Loeser, J.D., Melzack, R. (1999). Pain: an overview. *The Lancet*,353, 1607-1609.

Lorig, K., Gonzalez, V.M., Laurent, D.D., Morgan, L., Laris, B.A. (1998). Arthritis self-management program variations: three studies. *Arthritis Care Res., 11*, 448-454.

Lorig, K.R., Ritter, P., Stewart, A.L., Sobel, D.S., Brown, B.W.Jr., Bandura, A., Gonzalez, V.M., Laurent, D.D., Holman, H.R. (2001). Chronic disease self-management program: 2-year health status and health care utilization outcomes. *Med.Care, 39*, 1217-1223.
La Société d'arthrite. La Société d'arthrite. 2007.

Maquet, D., Croisier, J.L., Crielaard, J.M. (2001). Le point sur la fibromyalgia. *Ann Réadaptation Méd Phys, 44*, 316-325.

Mannerkorpi, K., Nyberg, B., Ahlmén, M., Ekdahl, C. (2000). Pool exercise combined with an education program for patients with fibromyalgia syndrome. A prospective, randomized study. *The Journal of Rheumatology, 27(*10), 2473-2481.

Mannerkorpi, K., Iversen, M.D. (2003). Physical exercise in fibromyalgia and related syndromes. *Best practice & research, 17*(4), 629-647.

Mantha, M.M., Lefrançois, P. (2007). Lombalgie. Site téléacessible à l'adresse <www.passeportsante.net>. Consulté le 5 février 2008.

Marchand, S. (1998). *Le phénomène de la douleur*. Chenelièvere / McGraw-Hill, Montreal.

Marty, M., Rozenberg, S., Duplan, B., Thomas, P., Duquesnoy, B., Allaert, F., et la Section Rachis de la Société Française de Rhumatologie. (2004). Qualité du sommeil et lombalgie chronique. *Douleurs, 5*(5), 29.

McNally, J.D., Matheson, D.A., Bakowsky, V.S. (2006). The epidemiology of self-reported fibromyalgia in Canada. *Chronic Dis Can*, 27, 9-16.

McWilliams, L. A., Cox, B. J., Enns, M. W. (2003). Mood and anxiety disorders associated with chronic pain: An examination in a nationally representative sample. *Pain*, 106, 126-133.

Melzack, R., Wall, P.D. (1988). The challenge of pain. Penguin Books, London.

Merskey, H., Bogduk, N. (1994). *Classification of Chronic Pain*, 2nd ed. IASP Press, Seattle.

Millar, W.J. (1996). La douleur chronique. *Rapports sur la santé - Statistiques Canada , 7*, 51-58.

Moulin, D.E., Clark, A.J. Speechley, M., Morley-Forster, P.K. (2002). Chronic pain in Canada: prevalence, treatment, impact and the role of opioid analgesia. Pain Research and Management, 7(4), 179-184.

Ohayon, M.M., Schatzberg, A.F. (2003). Using chronic pain to predict depressive morbidity in the general population. *Arch.Gen.Psychiatry*, 60:39-47.

Passik, S.D., Kirsh, K.L., McDonald, M.V., Ahn, S., Russak, S.M., Martin, L., Rosenfeld, B., Breitbart, W., Portenoy, R.K. (2000). A pilot survey of aberrant drug-taking attitudes and behaviors in samples of cancer and AIDS patients. *Journal Pain Symptom Manage, 19*, 274-286.

Peeples, P.J., Wertheimer, A.I., Mackowiak, J.I., McGhan. W.F. (1997). Controversies in measuring and valuing indirect costs of productivity foregone in a cost of illness evaluation. *J Res Pharm Econ, 8*, 23-32.

Perrot, S., Dumont, D., Guillemin, F., Pouchot, J., Coste, J. (2003). Quality of life in women with fibromyalgia syndrome: validation of the QIF, the French version of the fibromyalgia impact questionnaire. *The Journal of Rheumatology, 30*(5), 1054-1059.

Picavet, H.S., Hazes, J.M.W. (2003). Prevalence of self reported musculoskeletal diseases is high. *Annals of the Rheumatic Diseases, 62*(7), 644-650.

Poiraudeau, S., Nys, A., Revel, M. (2001). Évaluation analytique des moyens thérapeutiques dans la lombalgie : prise en charge physique et fonctionnelle. *Revue de Rhumatisme (Ed. Fr.), 68*, p. 154-159.

Réseau Québécois de l'Asthme et de la MPOC. (2007) Réseau Québécois de l'Asthme et de la MPOC .

Roques, C.F., Felez, A., Marque, P., Chatain, E., Lazorthes, Y. (2002). Étude de faisabilité d`un programme d`évaluation de l`École du dos. *Annales Réadaptation et Médecine Physique, 45*, 257-264.

Romano, J.M., Turner, J.A., Jensen, M.P. (1997). The family environment in chronic pain patients: comparison to controls and relationship to patient functioning. *Journal of Clinical Psychology in Medical Settings, 4*(4), 383-395.

Rosenstiel, A.K., Keefe, F.J. (1983). The use of coping strategies in chronic low back pain patients: relationship to patient characteristics and current adjustment. *Pain, 17*, 33-34.

Rossi, P.H., Freeman, H.E. (1993). Evaluation, a systematic Approach (5th ed.). California: sage Publications Inc.

Rossignol, M., Arsenault, B. (2006). *Guide de pratique clinique CLIP*. Clinique des Lombalgies Interdisciplinaire en Première ligne. Direction de santé publique : Agence de la santé et des services sociaux de Montréal.

Rothermich, E.A., Pathak, D.S. (1999). Productivity-cost controversies in cost-effectiveness analysis: review and research agenda. *Clin.Ther. 21*(1), 255-267.

Sach, T.H., Whynes, D.K. (2003). Measuring indirect costs: is there a problem? *Appl.Health Econ.Health Policy*, *2*(3), 135-139.

Santé Québec. (1995). *Et la santé , ça va*? Rappport de l'Enquête sociale et de santé 1992-1993, volume 1. Ministère de la Santé et des Services sociaux: Les publications du Québec.

Schopflocher, D., Borowski, H., Harstall, C., Juzwishin, D., Ospina, M., Taenzer, P. (2003). Chronic Pain in Alberta: A Portrait from the 1996 National Population Health Survey and the 2001 Canadian Community Health Survey. Edmonton, Alberta, Canada, Health Surveillance, Alberta Health, 1-26.

Schwartz, L., Slater, M.A., Birchler, G.R., Atkinson, J.H. (1991). Depression in spouses of chronic pain in patients: the role of patient pain and anger, and marital satisfaction. *Pain, 44,* 61-67.

Schwartz, L., Slater, M.A., Birchler, G.R. (1996). The role of pain behavior in the modulation of marital conflict in chronic pain couples. *Pain, 65*, 227-233.

Silver, J.K. (2004) *Chronic pain and the family effect on the couple*. A new guide. Havard University Press. Cambrigde, Massachusetts, 20-28.

Snellings, J. (1994). The effect of chronic pain on the family unit. J*ournal of Advanced Nursing, 19*, 543-551.

Spielberger, C. D. (2008). State-Trait Anxiety Inventory for Adults. Mind Garden®. Mind Garden, Inc. 11-9-0080. Site téléaccessible à l'adresse <http:// www.mindgarden.com/products/staisad.htm>. Consulté le 5 février 2009.

Spielberger, C.D., Gorsuch, R.L., Lushene, P.R., Vagg, P. R., Jacobs, A.G. (1983). Manual for the State-Trait Anxiety Inventory (Form Y). Consulting Psychologists Press, Inc.: Palo Alto, CA.

Spielberger, C. D., Krasner, S. S. (1988). *The assessment of State and Trait anxiety* (p.31-51). *In* 'Handbook of Anxiety (Vol. 2). (G. D. Burrows, M. Roth, R. J. NoyesEds.) Elsevier Science Publishers B.V.: Amsterdam.
Statistics Canada. *Health Indicators*. Catalogue no.82-221-XIE, Volume 2002, No. 1. 2002. Ottawa, Canada, Statistics Canada.

Stewart, W.F., Ricci, J.A., Chee, E., Morganstein, D., Lipton, R. (2003). Lost productive time and cost due to common pain conditions in the US workforce. *JAMA, 290*, 2443-2454.

Sullivan, M.J.L., Bishop, S., Pivik, J. (1995). The Pain Catastrophizing Scale: Development and validation. *Psychological Assessment, 7,* :524-532.

Sullivan, M.J.L., Thorn, B., Haythornthwaite, J.A., Keefe, F., Martin, M., Bradley, L.A., Lefebvre, J.C. (2001). Theorical perspectives on the relation between catastrophizing and pain. *The Clinical Journal of Pain, 17*, 52-64.

Turk, D.C., Dworkin, R.H., Allen, R.R., Bellamy, N., Brandenburg, N., Carr, D.B. (2003). Core outcome domains for chronic pain clinical trials: IMMPACT recommendations. *Pain, 106*, 337-345.

Tyler, E.J., Jensen, M.P., Schwartx, L. (2002). The reliability and validity of pain interference measures in persons with cerebral palsy. *Archives of Physycal Medecine and Rehabilitation, 83*, 236-239.

Valim, V., Oliveira, L., Suda, A., Silva, L., Assis, M., Barros Neto, T., Feldman, D., Natour, J. (2003). Aerobic fitness effect in fibromyalgia. *The Journal of Rheumatology, 30*(5),1060-1069.

Vannotti, M., Gennart, M. (2006). L'expéreince pathique de la douleur chronique: une approche phénoménologique. *Cahiers critiques de thérapie familiale et de pratiques de réseaux, 36*, 13-31.

Van Tulder, M.W., Koes, B.W., Bouter, L.M. (1995). A cost-of-illness study of back pain in The Netherlands. *Pain, 62*, 233-240.

Vierck, C.J., Staud, R., Price, D.D., Cannon, R.L., Mauderli, A.P., Martin, D. (2001). The effect of maximal exercise on temporal summation of second pain (windup) in patients with fibromyalgia syndrome. *The Journal of Pain, 2*(6), 334-344.

Villien, F., Yu, M., Barthelemy, P., Jammes, Y. (2005). Training to yoga respiration selectively increases respiratory sensation in healthy man. *Respir Physiol Neurobiol.*, 146, 85-96.

Wall, P.D. (2003).Text book of pain. *In* Wall, P.D., Melzack, R. T*he placebo and placebo reponse*, Churchill Livingstone, Toronto, (4nd ed).

Watson, D.E., Broemeling, A.M., Reid, R.J., Black. C. (2004). *A results-based logic model for primary health care: Laying an evidence-based foundation to guide performance measurement, monitoring and evaluation*. Centre for Health Services and Policy Research. Vancouver, College of Health Disciplines, The University of British Columbia.

Ware, J.E., Kosinski, M., Keller, S.D. (1996). A 12-item short-form health survey - Construction of scales and preliminary tests of reliability and validity. *Medical Care, 34*, 220-233.

Watzlawick, P., Nardone, G. (2000). *Stratégies de la thérapie brève*. Seuil, Paris.

White, K.P., Harth, M. (1999). The occurrence and impact of generalized pain. *Best practice and research, 13*(3), 379-389.

Wigers, S.H. (1996). Fibromyalgia outcome : the predictive value of symptom duration, physical activity, disability pension, and critical life events – a 4,5 year prospective study. *Journal of Psychosomatic Research, 41*(3), 235-243.

Williams, D.A., Cary, M.A., Groner, K.H., Chaplin, W., Glazer,I.J., Rodriguez, A.M., Clauw, D.J. (2002). Improving physical functional status in patients with fibromyalgia: a brief cognitive behavioral intervention. *The Journal of Rhuematology, 29*(6), 1280-1286.

Williams, D.A. (2002). Psychological and behavioral therapies in fibromyalgia and related syndromes. *Best practice and research, 17*(4), 649-665.

Wolfe, F., Anderson, J., Harkness, D., Bennett, R.M., Caro, X.J., Goldenberg, D.L., Russell, I.J., Yunus, M.B. (1997). A prospective, longitudinal, multicenter study of service utilization and costs in fibromyalgia. *Arthritis Rheum. 40*(9),1560-1570.

Wolfe, F., Smythe, H.A, Yunus, M.B., Bennett, R.M., Bombardier, C., Goldenberg, D. L., Tugwell, P., Campbell, E.M., Abeles, M., Clark, P., Fam, A.G., Fraber, S.J., Fiechtner, J.J., Franklin, C.M., Gatter, R.A., Hamaty, D., Lessard, J., Lichtbroun, A.S., Masi, A.T., McCain, G.A., Reynolds, J., Romano, T.J., Russell, I.J., Sheon, R.P. (1990). The American college of rheumatology 1990 criteria for the classification of fibromyalgia – report of multicenter criteria committee. *Arthritis and Rheumatism, 33*(2), 160-172.

Annexe 1

Annonce pour le recrutement

Université du Québec en Abitibi-Témiscamingue

Centre hospitalier universitaire de Sherbrooke
Centre de recherche clinique Étienne-Le Bel

ACCORD
Regroupement des forces pour gérer la douleur chronique

RECHERCHE SUR LA DOULEUR

Nous sommes à la recherche de participants et de participantes pour évaluer une intervention nommée « Écoles interactionnelles » visant à apprendre à gérer la douleur et les principaux symptômes associés à la

Fibromyalgie
et la
Lombalgie (maux de dos)

Pour être éligible, vous devez:

- Souffrir de fibromyalgie ou de maux de dos
- Être âgé(e) de 18 ans et plus
- Être intéressé(e) à appliquer diverses stratégies pour diminuer votre douleur (exercices physiques, relaxation, gestion du stress)
- Être disponible 3 heures semaine pour 9 rencontres réparties sur 3 mois

Informations

819-346-1110 poste **12304**

819-346-1110 poste **12304**

819-346-1110 poste **12304**

819-346-1110 poste **12304**

819-346-1110 poste **12304**

819-346-1110 poste **12304**

819-346-1110 poste **12304**

819-346-1110 poste **12304**

819-346-1110 poste **12304**

819-346-1110 poste **12304**

Annexe 2

Formulaire de présélection

Formulaire de pré-sélection ÉIF et ÉIL

Date : ________________________

Nom : ________________________

Numéro de téléphone : ___________________

-Comment la personne a entendu parler de l'étude? (encerclez)
Annonce journal   Médecin tx   Association   Bouche-à-oreille   Autre

-Brève présentation des Écoles

-Si intérêt, vérification des critères de sélection (répondre oui/non) :

Critères d'inclusion :

- Avoir un diagnostic médical de FM primaire posé depuis au moins 6 mois et établi à partir des critères de l'American College of Rheumatology—i.e., 11/18 points de sensibilité à la douleur générée par une pression équivalente à 4kg (Wolfe et al 1990)
  OU
- Avoir un diagnostic médical de LB non spécifique posé depuis au moins 6 mois (Chou et al 2007)

- Être âgé (e) de 18 à 65 ans
- Être capable de lire, comprendre et compléter des questionnaires en français
- Rapporter une douleur d'intensité moyenne plus grande ou égale à 4/10 au cours des 7 jours précédant l'évaluation initiale.
- Accepter d'être assigné(e) au hasard à l'une ou l'autre des conditions expérimentales.
- Être intéressé(e) et motivé(e) par le type d'intervention proposé
- Accepter de participer à 9 rencontres hebdomadaires étalées sur une période de 11 semaines et à une visite de relance.
- Maintenir un traitement médicamenteux contre la douleur qui soit stable ou ne prendre aucun analgésique pour la durée de l'étude

**À noter que les groupes FM (intervention ou contrôle) ne seront constitués que de femmes.*

Critères d'exclusion :

- Être enceinte ou en phase d'allaitement
- Être atteint d'un cancer actif ou d'une maladie d'origine métabolique non contrôlée
- Souffrir de douleur chronique d'origine autre que FM ou LB (arthrite rhumatoïde, neuropathie diabétique, etc.)
- Souffrir d'une co-morbidité physique ou psychiatrique majeure et suffisamment sévère pour altérer la capacité du sujet à participer à l'étude.
- Bénéficier d'indemnisations de la CSST ou de la SAAQ
- À noter que seront exclus en cours d'étude les sujets qui commencent un nouveau traitement ou analgésique (ou une thérapie physique ou psychologique).

-Si la personne répond aux critères, planifier la visite pour l'évaluation initiale.

-Donner les détails sur cette première visite.

-Si possible, poster le formulaire de consentement pour lecture à la maison.

-Donner le numéro de téléphone pour nous rejoindre au besoin (questions sur l'étude, annulation, etc.).

Date de l'évaluation initiale : ___________________________

Signature de la personne ayant fait cette pré-sélection

____

Annexe 3

Indices mesurés auprès des participants et temps de mesure

Tableau 2. Indices mesurés auprès des participants et temps de mesure

MESURES	PHASES DE L'ÉTUDE	
	**ENTREVUE Évaluation initiale (T_0)**	**SUIVI (T_1 à T_4)**
		(SI CHANGEMENTS)
**Caractéristiques sociodémographiques**		
• Sexe	X	--
• Âge	X	--
• Statut civil	X	X
• Groupe ethnique	X	--
• Langue première	X	--
• Conditions de vie actuelles	X	X
• Niveau de scolarité	X	--
• Statut actuel d'emploi	X	X
**Caractéristiques de la douleur**		
• Durée	X	--
• Fréquence	X	X
• Localisation	X	X
• Intensité (moyenne, pire, présente) (échelle numérique de 0-10) .	X	X
• Impact de la douleur (Items d'interférence du Questionnaire concis de la douleur modifié).	X	X
**Qualité du Sommeil**		
• *Chronic Pain Sleep Inventory*	X	X
**Impact spécifique du désordre douloureux**		
• Impact de la fibromyalgie (Questionnaire d'impact de la fibromyalgie/QIF)	X	X
• Incapacités engendrées par la lombalgie (Échelle d'incapacité du dos de Québec)	X	X
**Traitements analgésiques non pharmacologiques actuels**		
• Interventions médicales (p.ex., bloc, implant)	X	X
• Techniques psychologiques	X	X
• Thérapies physiques	X	X
• Autres	X	X
**Médication analgésique actuelle et autres produits contre la douleur utilisés présentement**		
• Type	X	X
• Posologie	X	X
• Fréquence d'administration	X	X

**Habitudes de consommation du patient**<br>• Cigarettes<br>• Alcool<br>• Drogues	 X<br>X<br>X	 X<br>X<br>X
**Stratégies d'ajustement face à la douleur**<br>• Échelle de dramatisation face à la douleur (PCS)<br>• *Coping Strategy Questionnaire* (CSQ-F)	 X<br>X	 X<br>X
**Fonctionnement psychologique**<br>• Dépression (BDI 1)	 X	 X
**Qualité de vie reliée à la santé**<br>• Qualité de vie physique et psychologique (SF-12 V2)	 X	 X
**Attentes du patient face à l'intervention**<br>• Attentes de changement (intensité de douleur, niveau fonctionnel, qualité de vie)<br>• Attentes re: degré de soulagement	 X<br><br>X	 --<br><br>--
**Impression globale de changement avec l'intervention 9ou groupe contrôle)**<br>• Impression globale de changement (intensité de douleur, niveau fonctionnel, qualité de vie)<br>• Degré de soulagement	 <br>--<br><br>--	 <br>X<br><br>X
**Coûts associés à la douleur : Utilisation des ressources de santé et perte de productivité**<br>• Hospitalisations, visites à l'urgence, visites chez un médecin omnipraticien, un médecin spécialiste ou tout autre professionnel de la santé.<br>• Achat de médicaments, produits naturels ou de fournitures.<br>• Utilisation d'aide domestique rémunérée.<br>• Absences au travail ou cessation du travail domestique non rémunéré.	 <br>X<br><br><br>X<br><br>X<br>X	T_1 à T_3 chez les groupes d'intervention<br>&<br>T_1 et T_2 chez les groupes contrôles

Annexe 4

Questionnaires et instruments de mesure s’adressant aux participants

IDENTIFICATION DU PATIENT<br>□□□-□□□<br>site # pt #	**QUESTIONNAIRE PARTICIPANT(E)**<br>**T0**	**ÉIF**
DATE DE LA VISITE<br>□□ - □□□ - 20□□<br>Jour Mois (ex: JUN) Année		

Pour usage administratif seulement

Complété:	☐ Oui	☐ Non	Si non, raison ____________
Appel téléphonique :	☐ Oui	☐ Non	Si oui, raison ____________

Vous trouverez dans ce document, une série de questions portant sur votre fibromyalgie et ses symptômes. Ces questions sont complétées dans le cadre de l'École interactionnelle de fibromyalgie.

Rappelez-vous qu'il n'y a pas de bonne ou de mauvaises réponses. Répondez aux questions au meilleur de votre connaissance.

✐ Vous devez compléter seul ce questionnaire. Néanmoins, si un problème physique limite votre capacité à écrire, un membre de votre famille ou un ami peut vous aider à écrire vos réponses aux questions, mais il/elle ne doit en aucun moment influencer vos choix.

1. Depuis combien de temps ressentez-vous votre douleur?

☐☐ jours ou ☐☐ mois ou ☐☐ années

2. Comment décrivez-vous la fréquence de votre douleur au cours des 7 derniers jours?

☐1 Présente continuellement

☐2 Présente occasionnellement:

↳ Pendant combien de jours avez-vous ressenti de la douleur au cours des 7 derniers jours? ☐ jours

☐3 Aucune douleur :

↳ Si vous n'avez pas ressenti de douleur au cours des 7 derniers jours, combien de jours vous avez ressenti de la douleur au cours du dernier mois? ☐☐jours

3. Indiquez les circonstances entourant l'apparition de votre douleur
(Cochez la ou les cases qui représentent le mieux votre situation)

☐1 Accident de travail

☐2 Accident avec véhicule motorisé

☐3 Accident à la maison

☐4 Accident de sport

☐5 Accident sur un lieu public

☐6 Durant ou à la suite d'un cancer

☐7 Durant ou à la suite d'une maladie (autre que le cancer) *Spécifiez*: ___________________________

☐8 À la suite d'une chirurgie. *Spécifiez*: ____

☐9 Mouvement /trauma répétitifs

☐10 Événement stressant

☐11 Aucun événement précis

☐12 Autre raison ou événement. *Spécifiez*: ____

4. Sur les diagrammes ci-dessous, coloriez la (ou les) régions où vous ressentez de la douleur.

5. Par la suite, indiquez à l'aide d'une flèche l'endroit qui interfère le plus avec votre vie quotidienne (UN SEUL ENDROIT)

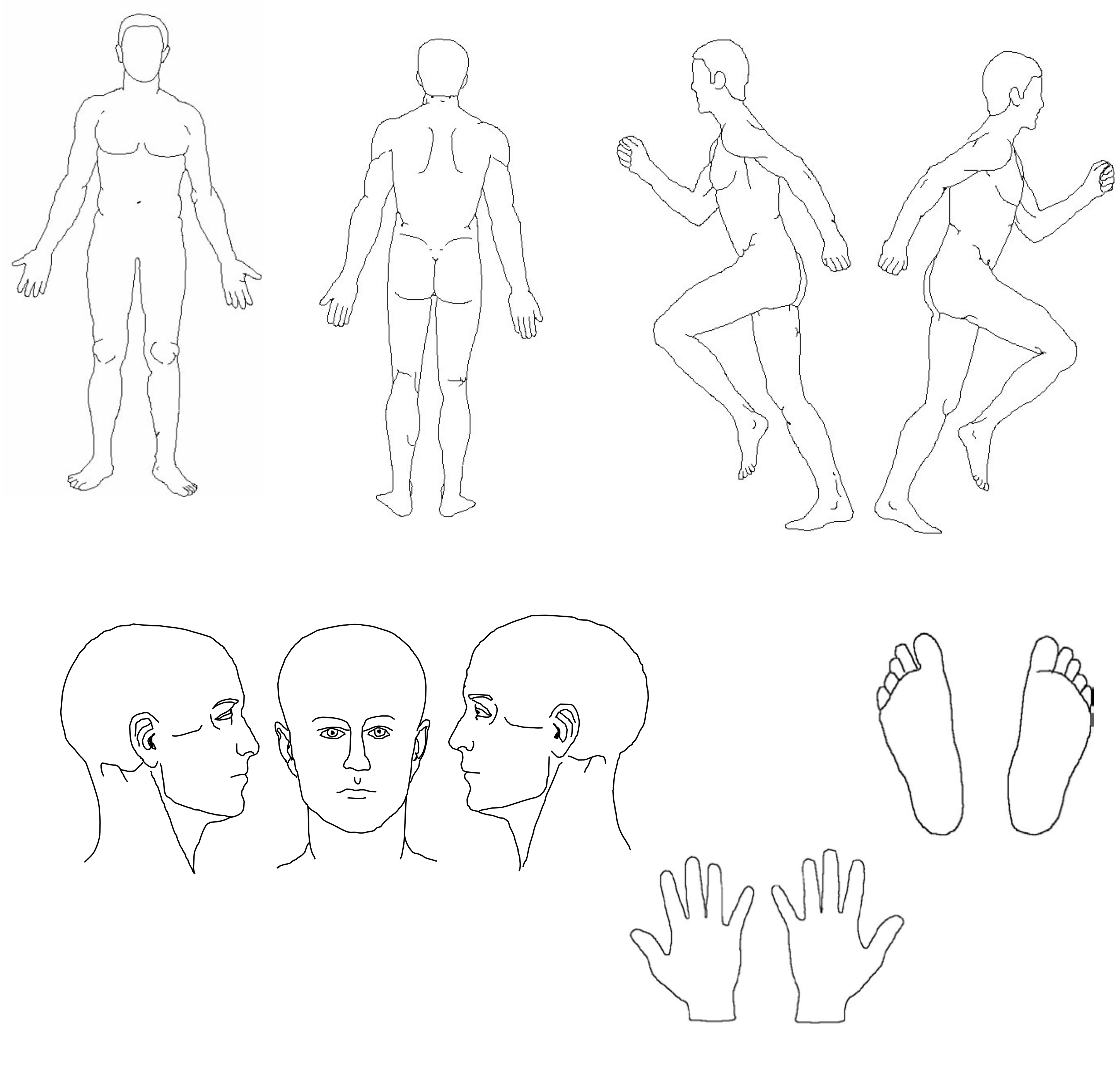

6. Veuillez répondre aux questions suivantes en ne tenant compte que de l'endroit douloureux qui interfère le plus avec votre vie quotidienne

Veuillez choisir sur l'échelle suivante le chiffre qui décrit le mieux :

1. La douleur que vous ressentez MAINTENANT

0	1	2	3	4	5	6	7	8	9	10
Aucune douleur										La pire douleur possible

2. La douleur que vous avez ressentie EN MOYENNE OU EN GÉNÉRAL au cours des 7 derniers jours

0	1	2	3	4	5	6	7	8	9	10
Aucune douleur										La pire douleur possible

3. LA PIRE douleur que vous avez ressentie au cours des 7 derniers jours

0	1	2	3	4	5	6	7	8	9	10
Aucune douleur										La pire douleur possible

*** Si vous n'avez pas ressenti de douleur au cours des 7 derniers jours, veuillez indiquer «0» aux questions a, b et c.

INVENTAIRE DE LA DOULEUR (BPI)

Veuillez choisir sur l'échelle suivante le chiffre qui décrit le mieux comment la douleur ressentie au cours des 7 derniers jours a interféré avec votre (vos):

Si vous n'avez ressenti aucune douleur au cours des 7 derniers jours, veuillez encercler « 0 » sur les échelles

A. Activité générale

	0	1	2	3	4	5	6	7	8	9	10
N'interfère pas											Interfère complètement

B. Humeur

	0	1	2	3	4	5	6	7	8	9	10
N'interfère pas											Interfère complètement

C. Capacité à marcher

	0	1	2	3	4	5	6	7	8	9	10
N'interfère pas											Interfère complètement

D. Travail (inclus le travail à la maison et à l'extérieur)

	0	1	2	3	4	5	6	7	8	9	10
N'interfère pas											Interfère complètement

E. Relations avec les autres

	0	1	2	3	4	5	6	7	8	9	10
N'interfère pas											Interfère complètement

F. Sommeil

	0	1	2	3	4	5	6	7	8	9	10
N'interfère pas											Interfère complètement

INVENTAIRE DE LA DOULEUR (BPI) (suite)

G. Goût de vivre

0	1	2	3	4	5	6	7	8	9	10
N'interfère pas										Interfère complètement

H. Soins personnels

0	1	2	3	4	5	6	7	8	9	10
N'interfère pas										Interfère complètement

I. Activités récréatives

0	1	2	3	4	5	6	7	8	9	10
N'interfère pas										Interfère complètement

J. Activités sociales

0	1	2	3	4	5	6	7	8	9	10
N'interfère pas										Interfère complètement

Êtes-vous capable de :<br>(Veuillez entourer le numéro qui décrit le mieux l'état général dans lequel vous vous trouvez actuellement)	Toujours	La plupart du temps	De temps en temps	Jamais
1. Faire les courses?	0	1	2	3
2. Faire la lessive à la machine?	0	1	2	3
3. Préparer à manger?	0	1	2	3
4. Faire la vaisselle à la main?	0	1	2	3
5. Passer l'aspirateur?	0	1	2	3
6. Faire les lits?	0	1	2	3
7. Marcher plusieurs centaines de mètres?	0	1	2	3
8. Aller voir des amis ou la famille?	0	1	2	3
9. Faire du jardinage?	0	1	2	3
10. Conduire une voiture?	0	1	2	3
11. Monter les escaliers?	0	1	2	3

Au cours des 7 derniers jours (encerclez le chiffre correspondant) :

12. Combien de jours vous-êtes vous senti(e) bien?

0 1 2 3 4 5 6 7

Si vous n'avez pas d'activité professionnelle, passez à la question 15

13. Combien de jours de travail avez vous manqué à cause de la fibromyalgie?

0 1 2 3 4 5 6 7

14. Les jours où vous avez travaillé, les douleurs ou d'autres problèmes liés à votre fibromyalgie vous ont-ils gêné (e) dans votre travail?

0 1 2 3 4 5 6 7 8 9 10

Aucune Gêne — Gêne très importante

Au cours des 7 derniers jours (encerclez le chiffre correspondant) :

15. Avez vous eu des douleurs?

0 1 2 3 4 5 6 7 8 9 10

Aucune douleur — Douleur très importante

16. Avez-vous été fatigué(e)?

0 1 2 3 4 5 6 7 8 9 10

Pas du tout fatigué(e) — Extrêmement fatigué(e)

17. Comment vous êtes-vous senti(e) le matin au réveil ?

0	1	2	3	4	5	6	7	8	9	10
Tout à fait reposé(e) au réveil										Extrêmement fatigué(e) au réveil

18. Vous êtes-vous senti(e) raide ?

0	1	2	3	4	5	6	7	8	9	10
Pas du tout raide										Extrêmement raide

19. Vous êtes-vous senti(e) tendu(e) ou inquiet(e) ?

0	1	2	3	4	5	6	7	8	9	10
Pas du tout tendu(e)										Extrêmement tendu(e)

20. Vous êtes-vous senti(e) déprimé(e) ?

0	1	2	3	4	5	6	7	8	9	10
Pas du tout déprimé(e)										Extrêmement déprimé(e)

SOMMEIL ET DOULEUR

Pour chacune des questions suivantes, choisissez le chiffre qui décrit le mieux à quelle fréquence la douleur a perturbé votre sommeil au cours des 4 dernières semaines

1. À quelle fréquence avez-vous eu de la difficulté à vous endormir à cause de la douleur?

   0 1 2 3 4 5 6 7 8 9 10

   Jamais Toujours

2. À quelle fréquence avez-vous eu besoin de somnifère pour vous aider à vous endormir?

   0 1 2 3 4 5 6 7 8 9 10

   Jamais Toujours

3. À quelle fréquence avez-vous été réveillé par votre douleur durant la nuit?

   0 1 2 3 4 5 6 7 8 9 10

   Jamais Toujours

4. À quelle fréquence avez-vous été réveillé par votre douleur le matin?

   0 1 2 3 4 5 6 7 8 9 10

   Jamais Toujours

5. Veuillez choisir sur l'échelle suivante le chiffre qui décrit le mieux la qualité globale de votre sommeil

   0 1 2 3 4 5 6 7 8 9 10

   Très mauvais Excellent

6. Aviez-vous des problèmes de sommeil avant l'apparition de votre douleur?

   ☐0 Non

   ☐1 Oui

   ☐2 Je ne me souviens pas

RCD

Chacun d'entre nous aura à subir des expériences douloureuses. Cela peut être la douleur associée aux maux de tête, un mal de dent, ou encore la douleur musculaire ou aux articulations. Il nous arrive souvent d'avoir à subir des expériences douloureuses telles que la maladie, une blessure, un traitement dentaire ou une intervention chirurgicale.

Dans le présent questionnaire, nous vous demandons de décrire le genre de pensées et d'émotions que vous avez quand vous avez de la douleur. Vous trouverez ci-dessous treize énoncés décrivant différentes pensées et émotions qui peuvent être associées à la douleur. Veuillez indiquer à quel point vous avez ces pensées et émotions quand vous avez de la douleur.

Quand j'ai de la douleur…	Pas du tout	Quelque peu	De façon modérée	Beaucoup	Tout le temps
1. J'ai peur qu'il n'y aura pas de fin à la douleur	0	1	2	3	4
2. Je sens que je ne peux pas continuer	0	1	2	3	4
3.C'est terrible et je pense que ça ne s'améliorera pas	0	1	2	3	4
4.C'est affreux et je sens que c'est plus fort que moi.	0	1	2	3	4
5.Je sens que je ne peux plus supporter la douleur	0	1	2	3	4
6. J'ai peur que la douleur s'empire	0	1	2	3	4
7. Je ne fais que penser à d'autres expériences douloureuses	0	1	2	3	4
8. Avec inquiétude, je souhaite que la douleur disparaisse	0	1	2	3	4
9. Je ne peux m'empêcher d'y penser	0	1	2	3	4
10. Je ne fais que penser à quel point ça fait mal	0	1	2	3	4
11.Je ne fais que penser à quel point je veux que la douleur disparaisse	0	1	2	3	4
12.Il n'y a rien que je puisse faire pour réduire l'intensité de la douleur	0	1	2	3	4
13. Je me demande si quelque chose de grave va se produire	0	1	2	3	4

Droits réservés © 1995
Michael JL Sullivan

CSQ

Indiquez dans quelle mesure vous utilisez les stratégies proposées ci-dessous pour faire face à votre douleur au quotidien :

Quand j'ai mal …	**Jamais**	**Parfois**	**Souvent**	**Toujours**
1. J'essaie de prendre de la distance par rapport à la douleur, comme si elle était dans le corps de quelqu'un d'autre.	0	1	2	3
2. Je sors de chez moi et je fais quelque chose comme aller au cinéma ou faire des courses.	0	1	2	3
3. J'essaie de penser à quelque chose d'agréable.	0	1	2	3
4. Je n'y pense pas comme si c'était une douleur mais plutôt comme une sensation de lourdeur, de chaleur.	0	1	2	3
5. C'est terrible et j'ai l'impression que jamais ça n'ira mieux.	0	1	2	3
6. Je me dis d'être courageux et de continuer malgré la douleur.	0	1	2	3
7. Je lis.	0	1	2	3
8. Je me dis que je peux dominer ma douleur.	0	1	2	3
9. Je prends mes médicaments.	0	1	2	3
10. Je compte ou je fredonne une chanson dans ma tête.	0	1	2	3
11. J'y pense comme si c'était une autre sensation, comme un engourdissement par exemple.	0	1	2	3
12. C'est affreux et j'ai l'impression que cela me submerge.	0	1	2	3
13. Je joue à des jeux dans ma tête pour éloigner mon esprit de la douleur.	0	1	2	3
14. J'ai l'impression que la vie ne vaut pas la peine d'être vécue.	0	1	2	3
15. Je sais qu'un jour je rencontrerais quelqu'un qui pourra m'aider et soulager un moment ma douleur.	0	1	2	3
16. Je marche beaucoup.	0	1	2	3
17. Je prie Dieu que ça ne dure pas longtemps.	0	1	2	3
18. J'essaie de ne pas y penser comme si c'était mon corps, mais plutôt comme quelque chose séparé de moi.	0	1	2	3
19. Je me détends, je me décontracte.	0	1	2	3
20. Je ne pense pas à la douleur.	0	1	2	3
21. J'essaie de penser à l'avenir, à ce que sera ma vie après que je me sois débarrassé de la douleur.	0	1	2	3

Quand j'ai mal …	Jamais	Parfois	Souvent	Toujour s
22. Je me dis que je n'ai pas mal.	0	1	2	3
23. Je me dis que je ne peux pas laisser la douleur gêner ce que j'ai à faire.	0	1	2	3
24. Je ne porte aucune attention à la douleur.	0	1	2	3
25. Je fais confiance aux médecins qui trouveront un jour un traitement pour ma douleur.	0	1	2	3
26. Peu importe l'intensité de la douleur, je sais que je peux y faire face.	0	1	2	3
27. Je fais comme si elle n'était pas là.	0	1	2	3
28. Je m'inquiète tout le temps de savoir si ça va finir.	0	1	2	3
29. Je me couche.	0	1	2	3
30. Je repense à des moments agréables du passé.	0	1	2	3
31. Je pense à des personnes avec lesquelles j'aime être.	0	1	2	3
32. Je prie pour que la douleur disparaisse.	0	1	2	3
33. Je prends une douche ou un bain.	0	1	2	3
34. J'imagine que la douleur est en dehors de mon corps.	0	1	2	3
35. Je continue comme si de rien n'était.	0	1	2	3
36. Je vois cela comme un défi et ne laisse pas la douleur me perturber.	0	1	2	3
37. Même si j'ai mal, je continue à faire ce que j'ai à faire.	0	1	2	3
38. J'ai l'impression de ne plus pouvoir supporter la douleur.	0	1	2	3
39. J'essaie de ne pas rester seul(e).	0	1	2	3
40. Je l'ignore.	0	1	2	3
41. Je compte sur ma foi en Dieu.	0	1	2	3
42. J'ai l'impression de ne plus pouvoir continuer.	0	1	2	3
43. Je pense aux choses que j'aime faire.	0	1	2	3
44. Je fais n'importe quoi pour éloigner mon esprit de la douleur.	0	1	2	3
45. Je fais quelque chose qui me plaît comme regarder la télévision ou écouter la musique.	0	1	2	3
46. Je fais comme si ça ne faisait pas partie de moi.	0	1	2	3
47. Je reste actif, par exemple en faisant des tâches ménagères ou des projets.	0	1	2	3
48. J'utilise une compresse chauffante.	0	1	2	3

BDI 1

Ce questionnaire contient des groupes d'énoncés. Lisez attentivement **tous les énoncés** pour chaque groupe, puis entourez le chiffre correspondant à l'énoncé qui décrit le **mieux** la façon dont vous vous êtes senti(e) au cours des **sept derniers jours, aujourd'hui compris.** Si plusieurs énoncés semblent convenir également bien, encerclez chacun d'eux. **Veuillez vous assurer d'avoir lu tous les énoncés de chaque groupe avant d'effectuer votre choix.**

1	0	Je ne me sens pas triste
	1	Je me sens triste
	2	Je suis tout le temps triste et je ne peux m'en sortir
	3	Je suis si triste que je ne peux le supporter
2	0	Je ne suis pas particulièrement découragé(e) par l'avenir
	1	Je me sens découragé(e) par l'avenir
	2	J'ai l'impression de n'avoir aucune attente dans la vie
	3	J'ai l'impression que l'avenir est sans espoir et que les choses ne peuvent s'améliorer
3	0	Je ne me considère pas comme un(e) raté(e)
	1	J'ai l'impression d'avoir subi plus d'échecs que le commun des mortels
	2	Quand je pense à mon passé, je ne vois que des échecs
	3	J'ai l'impression d'avoir complètement échoué dans la vie

BDI 1 (suite)

4	0	Je retire autant de satisfaction de la vie qu'auparavant
	1	Je ne retire plus autant de satisfaction de la vie qu'auparavant
	2	Je ne retire plus de satisfaction de quoi que ce soit
	3	Tout me rend insatisfait ou m'ennuie
5	0	Je ne me sens pas particulièrement coupable
	1	Je me sens coupable une bonne partie du temps
	2	Je me sens coupable la plupart du temps
	3	Je me sens continuellement coupable
6	0	Je n'ai pas l'impression d'être puni(e)
	1	J'ai l'impression que je pourrais être puni(e)
	2	Je m'attends à être puni(e)
	3	J'ai l'impression d'être puni(e)
7	0	Je n'ai pas l'impression d'être déçu(e) de moi
	1	Je suis déçu(e) de moi
	2	Je suis dégoûté(e) de moi
	3	Je me haïs

BDI 1 (suite)

8	0	Je n'ai pas l'impression d'être pire que quiconque
	1	Je suis critique de mes faiblesses ou de mes erreurs
	2	Je me blâme tout le temps pour mes erreurs
	3	Je me blâme pour tous les malheurs qui arrivent
9	0	Je ne pense aucunement à me suicider
	1	J'ai parfois l'idée de me suicider, mais je n'irais pas jusqu'à passer aux actes
	2	J'aimerais me suicider
	3	J'aimerais me suicider si j'en avais l'occasion
10	0	Je ne pleure pas plus qu'à l'ordinaire
	1	Je pleure plus qu'avant
	2	Je pleure continuellement maintenant
	3	Avant je pouvais pleurer, mais maintenant j'en suis incapable
11	0	Je ne suis pas plus irrité(e) maintenant qu'auparavant
	1	Je suis agacé(e) ou irrité(e) plus facilement qu'auparavant
	2	Je suis continuellement irrité(e)
	3	Je ne suis plus du tout irrité(e) par les choses qui m'irritaient auparavant

12	0	Je n'ai pas perdu mon intérêt pour les gens
	1	Je suis moins intéressé(e) par les gens qu'autrefois
	2	J'ai perdu la plupart de mon intérêt pour les gens
	3	J'ai perdu tout intérêt pour les gens
13	0	Je prends des décisions aussi facilement qu'avant
	1	Je remets des décisions beaucoup plus qu'auparavant
	2	J'ai beaucoup plus de difficulté à prendre des décisions qu'auparavant
	3	Je ne peux plus prendre de décisions
14	0	Je n'ai pas l'impression que mon apparence soit pire qu'auparavant
	1	J'ai peur de paraître vieux (vieille) ou peu attrayant(e)
	2	J'ai l'impression qu'il y a des changements permanents qui me rendent peu attrayant(e)
	3	J'ai l'impression d'être laid(e)
15	0	Je peux travailler aussi bien qu'avant
	1	Il faut que je fasse des efforts supplémentaires pour commencer quelque chose
	2	Je dois me secouer très fort pour faire quoi que ce soit
	3	Je ne peux faire aucun travail

BDI 1 (suite)

16	0	Je dors aussi bien que d’habitude
	1	Je ne dors pas aussi bien qu’avant
	2	Je me lève une à deux heures plus tôt qu’avant et j’ai du mal à me rendormir
	3	Je me réveille plusieurs heures plus tôt qu’avant et je ne peux me rendormir
17	0	Je ne me sens pas plus fatigué(e) qu’à l’accoutumé
	1	Je me fatigue plus facilement qu’auparavant
	2	Je me fatigue pour un rien
	3	Je suis trop fatigué(e) pour faire quoi que ce soit
18	0	Mon appétit n’est pas pire que d’habitude
	1	Mon appétit n’est pas aussi bon qu’il l’était
	2	Mon appétit a beaucoup diminué
	3	Je n’ai plus d’appétit du tout
19	0	Je n’ai pas perdu de poids dernièrement
	1	J’ai perdu plus de 5 livres
	2	J’ai perdu plus de 10 livres
	3	J’ai perdu plus de 15 livres

Je suis présentement un régime Oui _________ Non __________

BDI 1 (suite)

20	0	Ma santé ne me préoccupe pas plus que d'habitude
	1	Je suis préoccupé(e) par des problèmes de santé comme les douleurs, les maux d'estomac ou la constipation
	2	Mon état de santé me préoccupe beaucoup et il m'est difficile de penser à autre chose
	3	Je suis tellement préoccupé(e) par mon état de santé qu'il m'est impossible de penser à autre chose
21	0	Je n'ai remarqué récemment aucun changement dans mon intérêt pour le sexe
	1	J'ai moins de désirs sexuels qu'auparavant
	2	J'ai maintenant beaucoup moins de désirs sexuels
	3	J'ai perdu tout désir sexuel

HUMEUR

1) Jusqu'à quel point vous êtes-vous senti(e) en colère au cours des 7 dernier jours?

0 1 2 3 4 5 6 7 8 9 10

Pas du tout — Extrêmement

Votre Santé et Votre Bien-Être

Les questions qui suivent portent sur votre santé, telle que vous la percevez. Vos réponses permettront de suivre l'évolution de votre état de santé et de savoir dans quelle mesure vous pouvez accomplir vos activités courantes.

Pour chacune des questions suivantes, cochez la case ☒ correspondant le mieux à votre réponse.

1. En général, diriez-vous que votre santé est:

Excellente	Très bonne	Bonne	Passable	Mauvaise
▼	▼	▼	▼	▼
☐ 1	☐ 2	☐ 3	☐ 4	☐ 5

2. Les questions suivantes portent sur les activités que vous pourriez avoir à faire au cours d'une journée normale. <u>Votre état de santé actuel vous limite-t-il</u> dans ces activités? Si oui, dans quelle mesure?

	Mon état de santé me limite beaucoup	Mon état de santé me limite un peu	Mon état de santé ne me limite pas du tout
	▼	▼	▼
a) Dans les <u>activités modérées</u> comme déplacer une table, passer l'aspirateur, jouer aux quilles ou au golf	☐ 1	☐ 2	☐ 3
b) Pour monter <u>plusieurs</u> étages à pied	☐ 1	☐ 2	☐ 3

3. Au cours des <u>quatre dernières semaines</u>, combien de fois avez-vous eu l'une ou l'autre des difficultés suivantes au travail ou dans vos autres activités quotidiennes <u>à cause de votre état de santé physique</u>?

	Tout le temps	La plupart du temps	Parfois	Rare-ment	Jamais
a) <u>Avez-vous accompli moins</u> de choses que vous l'auriez voulu?	☐1	☐2	☐3	☐4	☐5
b) Avez-vous été limité(e) dans la <u>nature</u> de vos tâches ou de vos autres activités?	☐1	☐2	☐3	☐4	☐5

4. Au cours des <u>quatre dernières semaines</u>, combien de fois avez-vous eu l'une ou l'autre des difficultés suivantes au travail ou dans vos autres activités quotidiennes <u>à cause de l'état de votre moral</u> (comme le fait de vous sentir déprimé(e) ou anxieux(se))?

	Tout le temps	La plupart du temps	Parfois	Rare-ment	Jamais
a) Avez-vous <u>accompli moins</u> de choses que vous l'auriez voulu?	☐1	☐2	☐3	☐4	☐5
b) Avez-vous fait votre travail ou vos autres activités avec <u>moins de soin</u> qu'à l'habitude?	☐1	☐2	☐3	☐4	☐5

5. Au cours des quatre dernières semaines, dans quelle mesure la douleur a-t-elle nui à vos activités habituelles (au travail comme à la maison)?

Pas du tout	Un peu	Moyennement	Beaucoup	Énormément
☐1	☐2	☐3	☐4	☐5

6. Ces questions portent sur les quatre dernières semaines. Pour chacune des questions suivantes, donnez la réponse qui s'approche le plus de la façon dont vous vous êtes senti(e). Au cours des quatre dernières semaines, combien de fois:

	Tout le temps	La plupart du temps	Parfois	Rare-ment	Jamais
a) Vous êtes-vous senti(e) calme et serein(e)?	☐1	☐2	☐3	☐4	☐5
b) Avez-vous eu beaucoup d'énergie?	☐1	☐2	☐3	☐4	☐5
c) Vous êtes-vous senti(e) triste et démoralisé(e)?	☐1	☐2	☐3	☐4	☐5

7. Au cours des quatre dernières semaines, combien de fois votre état physique ou moral a-t-il nui à vos activités sociales (comme visiter des amis, des parents, etc.)?

Tout le temps	La plupart du temps	Parfois	Rarement	Jamais
☐1	☐2	☐3	☐4	☐5

SF-12v2™ Health Survey © 1994, 2002 Health Assessment Lab, Medical Outcomes Trust and Quality Metric Incorporated. All Rights Reserved.
SF-12® is a registered trademark of Medical Outcomes Trust.
(SF-12v2 Standard, Canada (French) Version 2.0)

ATTENTES

Avec l'École interactionnelle de fibromyalgie, vous attendez-vous à ce que, dans trois mois d'ici,

1. Votre douleur se soit :

- ☐0 Considérablement détériorée
- ☐1 Beaucoup détériorée
- ☐2 Un peu détériorée
- ☐3 Inchangée
- ☐4 Un peu améliorée
- ☐5 Beaucoup améliorée
- ☐6 Considérablement améliorée

2. Votre fonctionnement se soit:

- ☐0 Considérablement détérioré
- ☐1 Beaucoup détérioré
- ☐2 Un peu détérioré
- ☐3 Inchangé
- ☐4 Un peu amélioré
- ☐5 Beaucoup amélioré
- ☐6 Considérablement amélioré

Avec l'École interactionnelle de fibromyalgie, vous attendez-vous à ce que, dans trois mois d'ici,

3. Votre qualité de vie se soit :

☐0 Considérablement détériorée
☐1 Beaucoup détériorée
☐2 Un peu détériorée
☐3 Inchangée
☐4 Un peu améliorée
☐5 Beaucoup améliorée
☐6 Considérablement améliorée

4. Avec l'École interactionnelle de fibromyalgie, quel pourcentage de soulagement vous attendez-vous d'avoir dans trois mois d'ici?

	0 %	10	20	30	40	50	60	70	80	90	100%
Aucun soulagement											Soulagement complet

INFORMATION SOCIO-DÉMOGRAPHIQUES

1. DATE DE NAISSANCE :	☐☐ - ☐☐☐ - ☐☐☐☐<br>Jour mois (ex. : JUN) année	2. Sexe ☐1 Femme<br>☐2 Homme
3. ETHNICITÉ :<br>(Cochez la ou les cases appropriées)	☐ **Blanc** (personne ayant des ancêtres Originaires d'Europe, Afrique du Nord ou Moyen Orient)<br>☐ **Hispanique** (Mexicain, Porto Ricain, Cubain, Amérique centrale ou du Sud)<br>☐ **Noir** (personne ayant des ancêtres originaires d'Afrique ou d'ethnicité noire)<br>☐ **Asiatique** (personne ayant des ancêtres originaires d'Orient, d'Asie, d'Inde, Iles du Pacifique, c.-à-d., Chine, Japon, Philippines, Corée, Samoa, etc.)<br>☐ **Amérindien** (personne ayant des ancêtres originaires d'un groupe ou tribu des premières nations d'Amérique du Nord)<br>☐ **Autres** : (spécifiez) _________________	
4. LANGUES<br>Quelle est votre langue première?	☐1 Français<br>☐2 Anglais<br>☐3 Espagnol<br>☐4 Italien<br>☐5 Portugais<br>☐6 Allemand<br>☐7 Arabe<br>☐8 Chinois<br>☐9 Japonais<br>☐10 Vietnamien<br>☐11 Autres : spécifiez___________________	
5. SCOLARITÉ<br>(Cochez le plus haut niveau de scolarité complété)	☐0 Aucune<br>☐1 Primaire<br>☐2 Secondaire<br>☐3 École technique ou CEGEP<br>☐4 Universitaire	

6. CONDITION DE VIE ACTUELLE<br>Avec qui habitez-vous ?<br>(cochez la ou les cases appropriées)	☐ Seul (e)<br>☐ Conjoint (e)<br>☐ Enfant (s)<br>☐ Petits-enfants	☐ Parent (s)<br>☐ Frère, sœur etc.<br>☐ Colocataire (s)<br>☐ Pas de condition de vie stable
7. STATUT CIVIL	☐1 Célibataire<br>☐2 Marié(e) ou union libre<br>☐3 Séparé(e) ou divorcé(e)<br>☐4 Veuf (ve)	
8. TRAVAIL<br>Quelle est votre statut d'emploi <u>actuel</u>?<br>(cochez la ou les cases appropriées)	☐1 Travail à temps complet<br>☐2 Travail à temps partiel<br>☐3 À la maison<br>☐4 Étudiant(e)<br>☐5 Retraité(e)	☐6 Invalidité temporaire<br>☐7 Invalidité permanente<br>☐8 Sans emploi<br>☐9 Mise à pied<br>☐10 Bénévole<br>☐11 Autre. *Spécifiez:* ____________________________

9. REVENU FAMILIAL<br>*(A noter que toutes les informations recueillies dans ce questionnaire demeureront strictement confidentielles et seront traitées sur une base anonyme)*	Quelle catégorie représente le mieux votre revenu familial annuel avant les déductions ?	☐0 Moins de 20 000$<br>☐1 20 000 – 34 999$<br>☐2 35 000 – 49 999$<br>☐3 50 000 – 64 999$<br>☐4 65 000 – 79 999$	☐5 80 000 – 99 999$<br>☐6 100 000 – 119 999$<br>☐7 120 000$ et plus<br>☐8 Je ne désire pas répondre

PRINCIPALE SOURCE DE REVENU

1. Veuillez indiquer votre principale source de revenu. (*Sélectionnez un choix seulement)*

- ☐1 Revenu d'un emploi ou salaire
- ☐2 Prestations d'invalidité de la CSST (Commission de la Santé et Sécurité au travail)
- ☐3 Prestations d'invalidité de la SAAQ (Société d'assurance-automobile du Québec)
- ☐4 Prestations d'invalidité de l'IVAQ (Indemnisation des victimes d'actes criminels).
- ☐5 Prestations d'invalidité de l'employeur.
- ☐6 Prestations d'invalidité d'un programme d'assurance personnelle
- ☐7 Pension de retraite (pension de retraite, sécurité de la vieillesse ou RRQ)
- ☐8 Prestation d'invalidité du régime de pension du Canada
- ☐9 Assurance-emploi
- ☐10 Assistance sociale
- ☐11 Économies personnelles ou investissements
- ☐12 Revenu assuré pour handicap sévère
- ☐13 Membre de la famille
- ☐14 Autre. *Spécifiez*: ________________________________

2. Recevez-vous, actuellement, des prestations d'invalidité de

De la CSST (Commission de la Santé et Sécurité au travail)	☐0 Non	☐1 Oui
De la SAAQ (Société d'Assurances automobile du Québec)	☐0 Non	☐1 Oui
De l'IVAC (Indemnisation des victimes d'actes criminels)	☐0 Non	☐1 Oui
De l'employeur	☐0 Non	☐1 Oui
D'un programme d'assurance personnelle	☐0 Non	☐1 Oui

3. Y a-t-il des démarches en cours pour obtenir des prestations d'invalidité

De la CSST (Commission de la Santé et Sécurité au travail)	☐0 Non	☐1 Oui
De la SAAQ (Société d'Assurances automobile du Québec)	☐0 Non	☐1 Oui
De l'IVAC (Indemnisation des victimes d'actes criminels)	☐0 Non	☐1 Oui
De l'employeur	☐0 Non	☐1 Oui
D'un programme d'assurance personnelle	☐0 Non	☐1 Oui

4. Si oui, y a-t-il un litige en cours en lien avec cette réclamation ☐0 Non ☐1 Oui ☐2 non applicable

HABITUDES DE CONSOMMATION

CIGARETTES

1. Quel énoncé décrit le mieux vos habitudes par rapport à la cigarette?

☐0 Je n'ai jamais fumé
☐1 J'ai déjà fumé mais je ne fume plus: ↳ **1a.** nombre de cigarettes par jour ☐☐ **1b.** Nombre d'années ☐☐
☐2 Je suis un fumeur : ↳ **2a.** nombre de cigarettes par jour ☐☐ **2b.** Nombre d'années ☐☐

ALCOOL

Les questions suivantes portent sur votre consommation d'alcool. Lorsqu'on parle de "boisson alcoolisée" ou d'un "verre", on entend :« Une bouteille ou une canette de bière, ou un verre de bière en fût ; ou un verre de vin ou de boisson rafraîchissante au vin (« cooler ») ; ou un verre ou un cocktail contenant 1½ once de spiritueux. »

1. Au cours des <u>12 derniers mois</u>, à quelle fréquence avez-vous consommé des boissons alcoolisées?

☐0 Jamais	☐4 Une fois par semaine
☐1 Moins d'une fois par mois	☐5 2 à 3 fois par semaine
☐2 Une fois par mois	☐6 4 à 6 fois par semaine
☐3 2 à 3 fois par mois	☐7 Tous les jours

2. Habituellement, combien de verres buvez-vous, en moyenne par semaine? ☐☐ ou par mois? ☐☐

3. À n'importe quel moment au cours de votre vie:

	Non	Oui
a) Avez-vous déjà ressenti le besoin de diminuer votre consommation d'alcool?	☐0	☐1
b) Votre entourage vous a-t-il déjà fait des remarques au sujet de votre consommation d'alcool?	☐0	☐1
c) Avez-vous déjà éprouvé de la gêne ou de la culpabilité parce que vous consommiez de l'alcool?	☐0	☐1
d) Avez-vous déjà senti le besoin de prendre de l'alcool pour vous éveiller le matin?	☐0	☐1

DROGUES

1. Au cours des 12 derniers mois, à quelle fréquence avez-vous pris l'une ou l'autre des substances suivantes?

	Jamais	Moins d'une fois par mois	1 à 3 fois par mois	Une fois par semaine	Plus d'une fois par semaine	Chaque jour
a) Marijuana, cannabis ou hashish	☐0	☐1	☐2	☐3	☐4	☐5
b) Cocaïne ou crack	☐0	☐1	☐2	☐3	☐4	☐5
c) Héroïne	☐0	☐1	☐2	☐3	☐4	☐5
d) Ecstasy	☐0	☐1	☐2	☐3	☐4	☐5
e) Autres: *LSD, Mescaline, PCP, Acide…*	☐0	☐1	☐2	☐3	☐4	☐5

2. À n'importe quel moment au cours de votre vie:

	Non	Oui
a) Avez-vous déjà ressenti le besoin de diminuer votre consommation de drogue?	☐0	☐1
b) Votre entourage vous a-t-il déjà fait des remarques au sujet de votre consommation de drogue?	☐0	☐1
c) Avez-vous déjà éprouvé de la gêne ou de la culpabilité parce que vous consommiez de la drogue?	☐0	☐1
d) Avez-vous déjà senti le besoin de prendre de la drogue pour vous éveiller le matin?	☐0	☐1

Merci beaucoup d’avoir complété ce questionnaire.

***Veuillez vous assurer que vous avez bel et bien répondu à <u>toutes</u> les questions.
Il est très IMPORTANT pour nous qu'il n'y ait <u>pas</u> de données manquantes au moment des analyses.
Si certaines réponses sont manquantes, nous serons dans l'obligation de vous rappeler.

IDENTIFICATION DU PATIENT<br>☐☐☐-☐☐☐<br>site # pt #	**QUESTIONNAIRE PARTICIPANT(E)**<br>**T1-T2-T3-T4**	**ÉIF**
DATE DE LA VISITE<br>☐☐ - ☐☐☐ - 20☐☐<br>Jour Mois (ex: JUN) Année	DATE DE RECEPTION<br>☐☐ - ☐☐☐ - 20☐☐<br>Jour Mois (ex: JUN)<br>Année	

Pour usage administratif seulement			
Complété:	☐ Oui	☐ Non	Si non, raison ____________
Appel téléphonique :	☐ Oui	☐ Non	Si oui, raison ____________

Vous trouverez dans ce document, une série de questions portant sur votre fibromyalgie et ses symptômes. Ces questions sont complétées dans le cadre de l'École interactionnelle de fibromyalgie.

Rappelez-vous qu'il n'y a pas de bonne ou de mauvaises réponses. Répondez aux questions au meilleur de votre connaissance.

- ✐ Vous devez compléter seul ce questionnaire. Néanmoins, si un problème physique limite votre capacité à écrire, un membre de votre famille ou un ami peut vous aider à écrire vos réponses aux questions, mais il/elle ne doit en aucun moment influencer vos choix.
- ✐ Inscrire la date ou les dates auxquelles vous avez complété le questionnaire : ____________________

IMPORTANT :
ASSUREZ-VOUS DE COMPLÉTER TOUTES LES QUESTIONS

1. Depuis combien de temps ressentez-vous votre douleur?

☐☐ jours ou ☐☐ mois ou ☐☐ années

2. Comment décrivez-vous la fréquence de votre douleur au cours des 7 derniers jours?

☐1 Présente continuellement

☐2 Présente occasionnellement:

↳ Pendant combien de jours avez-vous ressenti de la douleur au cours des 7 derniers jours? ☐ jours

☐3 Aucune douleur :

↳ Si vous n'avez pas ressenti de douleur au cours des 7 derniers jours, combien de jours vous avez ressenti de la douleur au cours du dernier mois? ☐☐jours

3. Indiquez les circonstances entourant l'apparition de votre douleur
(Cochez la ou les cases qui représentent le mieux votre situation)

☐1 Accident de travail

☐2 Accident avec véhicule motorisé

☐3 Accident à la maison

☐4 Accident de sport

☐5 Accident sur un lieu public

☐6 Durant ou à la suite d'un cancer

☐7 Durant ou à la suite d'une maladie (autre que le cancer) *Spécifiez*: ____________________________

☐8 À la suite d'une chirurgie. *Spécifiez*: ____

☐9 Mouvement /trauma répétitifs

☐10 Événement stressant

☐11 Aucun événement précis

☐12 Autre raison ou événement. *Spécifiez*: ____

4. Sur les diagrammes ci-dessous, coloriez la (ou les) régions où vous ressentez de la douleur.

5. Par la suite, indiquez à l’aide d’une flèche l’endroit qui interfère le plus avec votre vie quotidienne (UN SEUL ENDROIT)

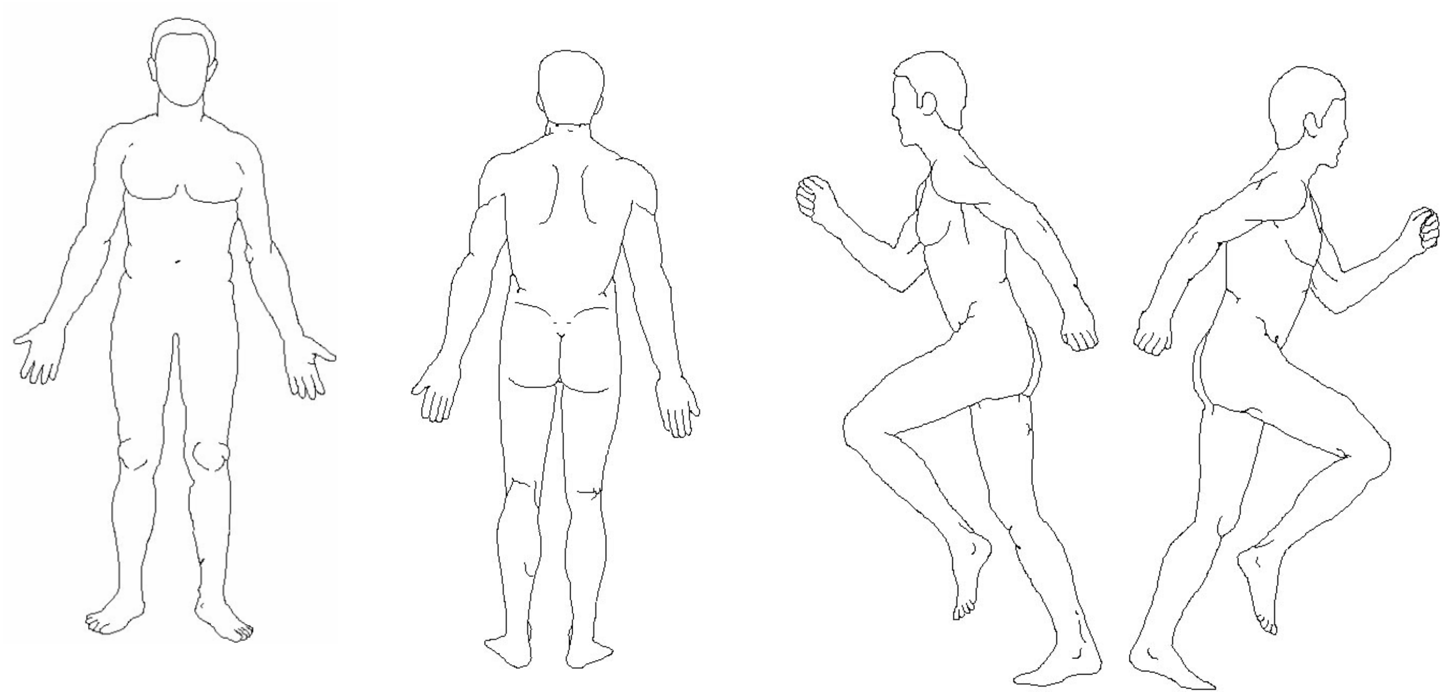

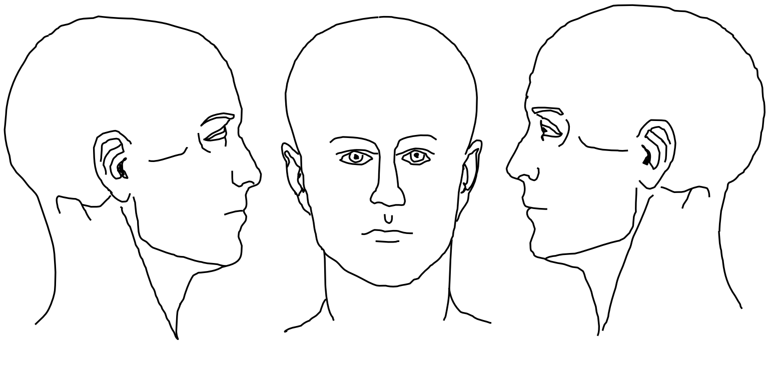

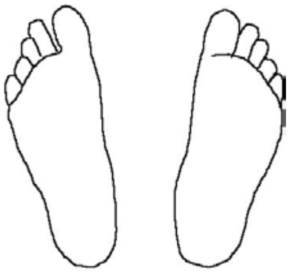

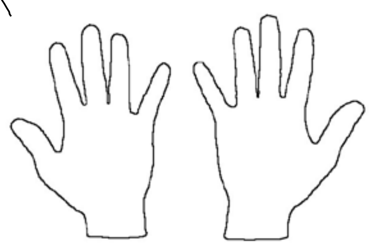

6. Veuillez répondre <u>aux questions suivantes</u> en ne tenant compte que de <u>l'endroit douloureux qui interfère le plus</u> avec votre vie quotidienne

Veuillez choisir sur l'échelle suivante le chiffre qui décrit le mieux :

5. La douleur que vous ressentez MAINTENANT

0	1	2	3	4	5	6	7	8	9	10
Aucune douleur										La pire douleur possible

6. La douleur que vous avez ressentie EN MOYENNE OU EN GÉNÉRAL au cours des <u>7 derniers jours</u>

0	1	2	3	4	5	6	7	8	9	10
Aucune douleur										La pire douleur possible

7. LA PIRE douleur que vous avez ressentie au cours des <u>7 derniers jours</u>

0	1	2	3	4	5	6	7	8	9	10
Aucune douleur										La pire douleur possible

*** Si vous n'avez pas ressenti de douleur au cours des 7 derniers jours, veuillez indiquer «0» aux questions a, b et c.

INVENTAIRE DE LA DOULEUR (BPI)

Veuillez choisir sur l'échelle suivante le chiffre qui décrit le mieux comment la douleur ressentie au cours des 7 derniers jours a interféré avec votre (vos):

Si vous n'avez ressenti aucune douleur au cours des 7 derniers jours, veuillez encercler « 0 » sur les échelles

A. Activité générale

0	1	2	3	4	5	6	7	8	9	10
N'interfère pas										Interfère complètement

B. Humeur

0	1	2	3	4	5	6	7	8	9	10
N'interfère pas										Interfère complètement

C. Capacité à marcher

0	1	2	3	4	5	6	7	8	9	10
N'interfère pas										Interfère complètement

D. Travail (inclus le travail à la maison et à l'extérieur)

0	1	2	3	4	5	6	7	8	9	10
N'interfère pas										Interfère complètement

E. Relations avec les autres

0	1	2	3	4	5	6	7	8	9	10
N'interfère pas										Interfère complètement

F. Sommeil

0	1	2	3	4	5	6	7	8	9	10
N'interfère pas										Interfère complètement

G. Goût de vivre

0	1	2	3	4	5	6	7	8	9	10
N'interfère pas										Interfère complètement

H. Soins personnels

0	1	2	3	4	5	6	7	8	9	10
N'interfère pas										Interfère complètement

I. Activités récréatives

0	1	2	3	4	5	6	7	8	9	10
N'interfère pas										Interfère complètement

J. Activités sociales

0	1	2	3	4	5	6	7	8	9	10
N'interfère pas										Interfère complètement

Êtes-vous capable de : (Veuillez entourer le numéro qui décrit le mieux l'état général dans lequel vous vous trouvez actuellement)	Toujours	La plupart du temps	De temps en temps	Jamais
1. Faire les courses?	0	1	2	3
2. Faire la lessive à la machine?	0	1	2	3
3. Préparer à manger?	0	1	2	3
4. Faire la vaisselle à la main?	0	1	2	3
5. Passer l'aspirateur?	0	1	2	3
6. Faire les lits?	0	1	2	3
7. Marcher plusieurs centaines de mètres?	0	1	2	3
8. Aller voir des amis ou la famille?	0	1	2	3
9. Faire du jardinage?	0	1	2	3
10. Conduire une voiture?	0	1	2	3
11. Monter les escaliers?	0	1	2	3

Au cours des 7 derniers jours (encerclez le chiffre correspondant) :

12. Combien de jours vous-êtes vous senti(e) bien?

0 1 2 3 4 5 6 7

Si vous n'avez pas d'activité professionnelle, passez à la question 15

13. Combien de jours de travail avez vous manqué à cause de la fibromyalgie?

0 1 2 3 4 5 6 7

14. Les jours où vous avez travaillé, les douleurs ou d'autres problèmes liés à votre fibromyalgie vous ont-ils gêné (e) dans votre travail?

0 1 2 3 4 5 6 7 8 9 10

Aucune Gêne — Gêne très importante

Au cours des 7 derniers jours (encerclez le chiffre correspondant) :

15. Avez vous eu des douleurs?

0 1 2 3 4 5 6 7 8 9 10

Aucune douleur — Douleur très importante

16. Avez-vous été fatigué(e)?

0 1 2 3 4 5 6 7 8 9 10

Pas du tout fatigué(e) — Extrêmement fatigué(e)

17. Comment vous êtes-vous senti(e) le matin au réveil ?

0	1	2	3	4	5	6	7	8	9	10

Tout à fait reposé(e) au réveil — Extrêmement fatigué(e) au réveil

18. Vous êtes-vous senti(e) raide ?

0	1	2	3	4	5	6	7	8	9	10

Pas du tout raide — Extrêmement raide

19. Vous êtes-vous senti(e) tendu(e) ou inquiet(e) ?

0	1	2	3	4	5	6	7	8	9	10

Pas du tout tendu(e) — Extrêmement tendu(e)

20. Vous êtes-vous senti(e) déprimé(e) ?

0	1	2	3	4	5	6	7	8	9	10

Pas du tout déprimé(e) — Extrêmement déprimé(e)

SOMMEIL ET DOULEUR

Pour chacune des questions suivantes, choisissez le chiffre qui décrit le mieux à quelle fréquence la douleur a perturbé votre sommeil au cours des 4 dernières semaines

1. À quelle fréquence avez-vous eu de la difficulté à vous endormir à cause de la douleur?

   0 1 2 3 4 5 6 7 8 9 10
   Jamais Toujours

2. À quelle fréquence avez-vous eu besoin de somnifère pour vous aider à vous endormir?

   0 1 2 3 4 5 6 7 8 9 10
   Jamais Toujours

3. À quelle fréquence avez-vous été réveillé par votre douleur durant la nuit?

   0 1 2 3 4 5 6 7 8 9 10
   Jamais Toujours

4. À quelle fréquence avez-vous été réveillé par votre douleur le matin?

   0 1 2 3 4 5 6 7 8 9 10
   Jamais Toujours

5. Veuillez choisir sur l'échelle suivante le chiffre qui décrit le mieux la qualité globale de votre sommeil

   0 1 2 3 4 5 6 7 8 9 10
   Très mauvais Excellent

6. Aviez-vous des problèmes de sommeil avant l'apparition de votre douleur?

☐0 Non
☐1 Oui
☐2 Je ne me souviens pas

Chacun d'entre nous aura à subir des expériences douloureuses. Cela peut être la douleur associée aux maux de tête, un mal de dent, ou encore la douleur musculaire ou aux articulations. Il nous arrive souvent d'avoir à subir des expériences douloureuses telles que la maladie, une blessure, un traitement dentaire ou une intervention chirurgicale.

Dans le présent questionnaire, nous vous demandons de décrire le genre de pensées et d'émotions que vous avez quand vous avez de la douleur. Vous trouverez ci-dessous treize énoncés décrivant différentes pensées et émotions qui peuvent être associées à la douleur. Veuillez indiquer à quel point vous avez ces pensées et émotions quand vous avez de la douleur.

Quand j'ai de la douleur…	Pas du tout	Quelque peu	De façon modérée	Beaucoup	Tout le temps
1. J'ai peur qu'il n'y aura pas de fin à la douleur	0	1	2	3	4
2. Je sens que je ne peux pas continuer	0	1	2	3	4
3.C'est terrible et je pense que ça ne s'améliorera pas	0	1	2	3	4
4.C'est affreux et je sens que c'est plus fort que moi.	0	1	2	3	4
5.Je sens que je ne peux plus supporter la douleur	0	1	2	3	4
6. J'ai peur que la douleur s'empire	0	1	2	3	4
7. Je ne fais que penser à d'autres expériences douloureuses	0	1	2	3	4
8. Avec inquiétude, je souhaite que la douleur disparaisse	0	1	2	3	4
9. Je ne peux m'empêcher d'y penser	0	1	2	3	4
10. Je ne fais que penser à quel point ça fait mal	0	1	2	3	4
11.Je ne fais que penser à quel point je veux que la douleur disparaisse	0	1	2	3	4
12.Il n'y a rien que je puisse faire pour réduire l'intensité de la douleur	0	1	2	3	4
13. Je me demande si quelque chose de grave va se produire	0	1	2	3	4

Droits réservés © 1995
Michael JL Sullivan

CSQ

Indiquez dans quelle mesure vous utilisez les stratégies proposées ci-dessous pour faire face à votre douleur au quotidien :

Quand j'ai mal …	Jamais	Parfois	Souvent	Toujours
1. J'essaie de prendre de la distance par rapport à la douleur, comme si elle était dans le corps de quelqu'un d'autre.	0	1	2	3
2. Je sors de chez moi et je fais quelque chose comme aller au cinéma ou faire des courses.	0	1	2	3
3. J'essaie de penser à quelque chose d'agréable.	0	1	2	3
4. Je n'y pense pas comme si c'était une douleur mais plutôt comme une sensation de lourdeur, de chaleur.	0	1	2	3
5. C'est terrible et j'ai l'impression que jamais ça n'ira mieux.	0	1	2	3
6. Je me dis d'être courageux et de continuer malgré la douleur.	0	1	2	3
7. Je lis.	0	1	2	3
8. Je me dis que je peux dominer ma douleur.	0	1	2	3
9. Je prends mes médicaments.	0	1	2	3
10. Je compte ou je fredonne une chanson dans ma tête.	0	1	2	3
11. J'y pense comme si c'était une autre sensation, comme un engourdissement par exemple.	0	1	2	3
12. C'est affreux et j'ai l'impression que cela me submerge.	0	1	2	3

Quand j'ai mal …	Jamais	Parfois	Souvent	Toujours
13. Je joue à des jeux dans ma tête pour éloigner mon esprit de la douleur.	0	1	2	3
14. J'ai l'impression que la vie ne vaut pas la peine d'être vécue.	0	1	2	3
15. Je sais qu'un jour je rencontrerais quelqu'un qui pourra m'aider et soulager un moment ma douleur.	0	1	2	3
16. Je marche beaucoup.	0	1	2	3
17. Je prie Dieu que ça ne dure pas longtemps.	0	1	2	3
18. J'essaie de ne pas y penser comme si c'était mon corps, mais plutôt comme quelque chose séparé de moi.	0	1	2	3
19. Je me détend, je me décontracte.	0	1	2	3
20. Je ne pense pas à la douleur.	0	1	2	3
21. J'essaie de penser à l'avenir, à ce que sera ma vie après que je me sois débarrassé de la douleur.	0	1	2	3
22. Je me dis que je n'ai pas mal.	0	1	2	3
23. Je me dis que je ne peux pas laisser la douleur gêner ce que j'ai à faire.	0	1	2	3
24. Je ne porte aucune attention à la douleur.	0	1	2	3
25. Je fais confiance aux médecins qui trouveront un jour un traitement pour ma douleur.	0	1	2	3
26. Peu importe l'intensité de la douleur, je sais que je peux y faire face.	0	1	2	3

27. Je fais comme si elle n'était pas là.	0	1	2	3
28. Je m'inquiète tout le temps de savoir si ça va finir.	0	1	2	3
29. Je me couche.	0	1	2	3
30. Je repense à des moments agréables du passé.	0	1	2	3
31. Je pense à des personnes avec lesquelles j'aime être.	0	1	2	3
32. Je prie pour que la douleur disparaisse.	0	1	2	3
33. Je prends une douche ou un bain.	0	1	2	3
34. J'imagine que la douleur est en dehors de mon corps.	0	1	2	3
35. Je continue comme si de rien n'était.	0	1	2	3
36. Je vois cela comme un défi et ne laisse pas la douleur me perturber.	0	1	2	3
37. Même si j'ai mal, je continue à faire ce que j'ai à faire.	0	1	2	3
38. J'ai l'impression de ne plus pouvoir supporter la douleur.	0	1	2	3
39. J'essaie de ne pas rester seul(e).	0	1	2	3
40. Je l'ignore.	0	1	2	3
41. Je compte sur ma foi en Dieu.	0	1	2	3

42. J’ai l’impression de ne plus pouvoir continuer.	0	1	2	3
43. Je pense aux choses que j’aime faire.	0	1	2	3
44. Je fais n’importe quoi pour éloigner mon esprit de la douleur.	0	1	2	3
45. Je fais quelque chose qui me plaît comme regarder la télévision ou écouter la musique.	0	1	2	3
46. Je fais comme si ça ne faisait pas partie de moi.	0	1	2	3
47. Je reste actif, par exemple en faisant des tâches ménagères ou des projets.	0	1	2	3
48. J’utilise une compresse chauffante.	0	1	2	3

BDI 1

Ce questionnaire contient des groupes d'énoncés. Lisez attentivement tous les énoncés pour chaque groupe, puis entourez le chiffre correspondant à l'énoncé qui décrit le mieux la façon dont vous vous êtes senti(e) au cours des sept derniers jours, aujourd'hui compris. Si plusieurs énoncés semblent convenir également bien, encerclez chacun d'eux. Veuillez vous assurer d'avoir lu tous les énoncés de chaque groupe avant d'effectuer votre choix.

1	0	Je ne me sens pas triste
	1	Je me sens triste
	2	Je suis tout le temps triste et je ne peux m'en sortir
	3	Je suis si triste que je ne peux le supporter
2	0	Je ne suis pas particulièrement découragé(e) par l'avenir
	1	Je me sens découragé(e) par l'avenir
	2	J'ai l'impression de n'avoir aucune attente dans la vie
	3	J'ai l'impression que l'avenir est sans espoir et que les choses ne peuvent s'améliorer
3	0	Je ne me considère pas comme un(e) raté(e)
	1	J'ai l'impression d'avoir subi plus d'échecs que le commun des mortels
	2	Quand je pense à mon passé, je ne vois que des échecs
	3	J'ai l'impression d'avoir complètement échoué dans la vie

BDI 1 (suite)

4	0	Je retire autant de satisfaction de la vie qu'auparavant
	1	Je ne retire plus autant de satisfaction de la vie qu'auparavant
	2	Je ne retire plus de satisfaction de quoi que ce soit
	3	Tout me rend insatisfait ou m'ennuie
5	0	Je ne me sens pas particulièrement coupable
	1	Je me sens coupable une bonne partie du temps
	2	Je me sens coupable la plupart du temps
	3	Je me sens continuellement coupable
6	0	Je n'ai pas l'impression d'être puni(e)
	1	J'ai l'impression que je pourrais être puni(e)
	2	Je m'attends à être puni(e)
	3	J'ai l'impression d'être puni(e)
7	0	Je n'ai pas l'impression d'être déçu(e) de moi
	1	Je suis déçu(e) de moi
	2	Je suis dégoûté(e) de moi
	3	Je me haïs

BDI 1 (suite)

8	0	Je n'ai pas l'impression d'être pire que quiconque
	1	Je suis critique de mes faiblesses ou de mes erreurs
	2	Je me blâme tout le temps pour mes erreurs
	3	Je me blâme pour tous les malheurs qui arrivent
9	0	Je ne pense aucunement à me suicider
	1	J'ai parfois l'idée de me suicider, mais je n'irais pas jusqu'à passer aux actes
	2	J'aimerais me suicider
	3	J'aimerais me suicider si j'en avais l'occasion
10	0	Je ne pleure pas plus qu'à l'ordinaire
	1	Je pleure plus qu'avant
	2	Je pleure continuellement maintenant
	3	Avant je pouvais pleurer, mais maintenant j'en suis incapable
11	0	Je ne suis pas plus irrité(e) maintenant qu'auparavant
	1	Je suis agacé(e) ou irrité(e) plus facilement qu'auparavant
	2	Je suis continuellement irrité(e)
	3	Je ne suis plus du tout irrité(e) par les choses qui m'irritaient auparavant

BDI 1 (suite)

12	0	Je n'ai pas perdu mon intérêt pour les gens
	1	Je suis moins intéressé(e) par les gens qu'autrefois
	2	J'ai perdu la plupart de mon intérêt pour les gens
	3	J'ai perdu tout intérêt pour les gens
13	0	Je prends des décisions aussi facilement qu'avant
	1	Je remets des décisions beaucoup plus qu'auparavant
	2	J'ai beaucoup plus de difficulté à prendre des décisions qu'auparavant
	3	Je ne peux plus prendre de décisions
14	0	Je n'ai pas l'impression que mon apparence soit pire qu'auparavant
	1	J'ai peur de paraître vieux (vieille) ou peu attrayant(e)
	2	J'ai l'impression qu'il y a des changements permanents qui me rendent peu attrayant(e)
	3	J'ai l'impression d'être laid(e)
15	0	Je peux travailler aussi bien qu'avant
	1	Il faut que je fasse des efforts supplémentaires pour commencer quelque chose
	2	Je dois me secouer très fort pour faire quoi que ce soit
	3	Je ne peux faire aucun travail

BDI 1 (suite)

16	0	Je dors aussi bien que d'habitude
	1	Je ne dors pas aussi bien qu'avant
	2	Je me lève une à deux heures plus tôt qu'avant et j'ai du mal à me rendormir
	3	Je me réveille plusieurs heures plus tôt qu'avant et je ne peux me rendormir
17	0	Je ne me sens pas plus fatigué(e) qu'à l'accoutumé
	1	Je me fatigue plus facilement qu'auparavant
	2	Je me fatigue pour un rien
	3	Je suis trop fatigué(e) pour faire quoi que ce soit
18	0	Mon appétit n'est pas pire que d'habitude
	1	Mon appétit n'est pas aussi bon qu'il l'était
	2	Mon appétit a beaucoup diminué
	3	Je n'ai plus d'appétit du tout
19	0	Je n'ai pas perdu de poids dernièrement
	1	J'ai perdu plus de 5 livres
	2	J'ai perdu plus de 10 livres
	3	J'ai perdu plus de 15 livres

Je suis présentement un régime Oui _________ Non __________

BDI 1 (suite)

20	0	Ma santé ne me préoccupe pas plus que d'habitude
	1	Je suis préoccupé(e) par des problèmes de santé comme les douleurs, les maux d'estomac ou la constipation
	2	Mon état de santé me préoccupe beaucoup et il m'est difficile de penser à autre chose
	3	Je suis tellement préoccupé(e) par mon état de santé qu'il m'est impossible de penser à autre chose
21	0	Je n'ai remarqué récemment aucun changement dans mon intérêt pour le sexe
	1	J'ai moins de désirs sexuels qu'auparavant
	2	J'ai maintenant beaucoup moins de désirs sexuels
	3	J'ai perdu tout désir sexuel

HUMEUR

1) Jusqu'à quel point vous êtes-vous senti(e) en <u>colère</u> au cours des 7 <u>dernier jours</u>?

0 1 2 3 4 5 6 7 8 9 10

Pas du tout — Extrêmement

Votre Santé et Votre Bien-Être

Les questions qui suivent portent sur votre santé, telle que vous la percevez. Vos réponses permettront de suivre l'évolution de votre état de santé et de savoir dans quelle mesure vous pouvez accomplir vos activités courantes.

Pour chacune des questions suivantes, cochez la case ☒ correspondant le mieux à votre réponse.

1. En général, diriez-vous que votre santé est:

Excellente	Très bonne	Bonne	Passable	Mauvaise
▼	▼	▼	▼	▼
☐ 1	☐ 2	☐ 3	☐ 4	☐ 5

5. Les questions suivantes portent sur les activités que vous pourriez avoir à faire au cours d'une journée normale. Votre état de santé actuel vous limite-t-il dans ces activités? Si oui, dans quelle mesure?

	Mon état de santé me limite beaucoup	Mon état de santé me limite un peu	Mon état de santé ne me limite pas du tout
	▼	▼	▼
a) Dans les activités modérées comme déplacer une table, passer l'aspirateur, jouer aux quilles ou au golf	☐ 1	☐ 2	☐ 3
b) Pour monter plusieurs étages à pied	☐ 1	☐ 2	☐ 3

6. Au cours des quatre dernières semaines, combien de fois avez-vous eu l'une ou l'autre des difficultés suivantes au travail ou dans vos autres activités quotidiennes à cause de votre état de santé physique?

	Tout le temps	La plupart du temps	Parfois	Rare-ment	Jamais
a) Avez-vous accompli moins de choses que vous l'auriez voulu?	☐1	☐2	☐3	☐4	☐5
b) Avez-vous été limité(e) dans la nature de vos tâches ou de vos autres activités?	☐1	☐2	☐3	☐4	☐5

7. Au cours des quatre dernières semaines, combien de fois avez-vous eu l'une ou l'autre des difficultés suivantes au travail ou dans vos autres activités quotidiennes à cause de l'état de votre moral (comme le fait de vous sentir déprimé(e) ou anxieux(se))?

	Tout le temps	La plupart du temps	Parfois	Rare-ment	Jamais
a) Avez-vous accompli moins de choses que vous l'auriez voulu?	☐1	☐2	☐3	☐4	☐5
b) Avez-vous fait votre travail ou vos autres activités avec moins de soin qu'à l'habitude?	☐1	☐2	☐3	☐4	☐5

5. Au cours des quatre dernières semaines, dans quelle mesure la douleur a-t-elle nui à vos activités habituelles (au travail comme à la maison)?

Pas du tout	Un peu	Moyennement	Beaucoup	Énormément
☐1	☐2	☐3	☐4	☐5

6. Ces questions portent sur les quatre dernières semaines. Pour chacune des questions suivantes, donnez la réponse qui s'approche le plus de la façon dont vous vous êtes senti(e). Au cours des quatre dernières semaines, combien de fois:

	Tout le temps	La plupart du temps	Parfois	Rarement	Jamais
a) Vous êtes-vous senti(e) calme et serein(e)?	☐1	☐2	☐3	☐4	☐5
b) Avez-vous eu beaucoup d'énergie?	☐1	☐2	☐3	☐4	☐5
c) Vous êtes-vous senti(e) triste et démoralisé(e)?	☐1	☐2	☐3	☐4	☐5

8. Au cours des quatre dernières semaines, combien de fois votre état physique ou moral a-t-il nui à vos activités sociales (comme visiter des amis, des parents, etc.)?

Tout le temps	La plupart du temps	Parfois	Rarement	Jamais
☐1	☐2	☐3	☐4	☐5

SF-12v2™ Health Survey © 1994, 2002 Health Assessment Lab, Medical Outcomes Trust and Quality Metric Incorporated. All Rights Reserved.
SF-12® is a registered trademark of Medical Outcomes Trust.
(SF-12v2 Standard, Canada (French) Version 2.0)

HABITUDES DE CONSOMMATION

CIGARETTES

1. Quel énoncé décrit le mieux vos habitudes par rapport à la cigarette?

☐0 Je n'ai jamais fumé		
☐1 J'ai déjà fumé mais je ne fume plus:	**1a.** nombre de cigarettes par jour ☐ ☐	**1b.** Nombre d'années ☐ ☐
☐2 Je suis un fumeur :	**2a.** nombre de cigarettes par jour ☐☐	**2b.** Nombre d'années ☐ ☐

ALCOOL

Les questions suivantes portent sur votre consommation d'alcool. Lorsqu'on parle de "boisson alcoolisée" ou d'un "verre", on entend : « Une bouteille ou une canette de bière, ou un verre de bière en fût ; ou un verre de vin ou de boisson rafraîchissante au vin (« cooler ») ; ou un verre ou un cocktail contenant 1½ once de spiritueux. »

2. Au cours des 12 derniers mois, à quelle fréquence avez-vous consommé des boissons alcoolisées?

☐0 Jamais
☐1 Moins d'une fois par mois
☐2 Une fois par mois
☐3 2 à 3 fois par mois
☐4 Une fois par semaine
☐5 2 à 3 fois par semaine
☐6 4 à 6 fois par semaine
☐7 Tous les jours

2. Habituellement, combien de verres buvez-vous, en moyenne par semaine? ☐☐ ou par mois? ☐☐

3. À n'importe quel moment au cours de votre vie:	Non	Oui
a) Avez-vous déjà ressenti le besoin de diminuer votre consommation d'alcool?	☐0	☐1
b) Votre entourage vous a-t-il déjà fait des remarques au sujet de votre consommation d'alcool?	☐0	☐1
c) Avez-vous déjà éprouvé de la gêne ou de la culpabilité parce que vous consommiez de l'alcool?	☐0	☐1
d) Avez-vous déjà senti le besoin de prendre de l'alcool pour vous éveiller le matin?	☐0	☐1

DROGUES

1. Au cours des 12 derniers mois, à quelle fréquence avez-vous pris l’une ou l’autre des substances suivantes?

	Jamai s	Moins d’une fois par mois	1 à 3 fois par mois	Une fois par semaine	Plus d’une fois par semaine	Chaque jour
a) Marijuana, cannabis ou hashish	☐0	☐1	☐2	☐3	☐4	☐5
b) Cocaïne ou crack	☐0	☐1	☐2	☐3	☐4	☐5
c) Héroïne	☐0	☐1	☐2	☐3	☐4	☐5
d) Ecstasy	☐0	☐1	☐2	☐3	☐4	☐5
e) Autres : *LSD, Mescaline, PCP, Acide…*	☐0	☐1	☐2	☐3	☐4	☐5

2. À n’importe quel moment au cours de votre vie:	Non	Oui
a) Avez-vous déjà ressenti le besoin de diminuer votre consommation de drogue?	☐0	☐1
b) Votre entourage vous a-t-il déjà fait des remarques au sujet de votre consommation de drogue?	☐0	☐1
c) Avez-vous déjà éprouvé de la gêne ou de la culpabilité parce que vous consommiez de la drogue?	☐0	☐1
d) Avez-vous déjà senti le besoin de prendre de la drogue pour vous éveiller le matin?	☐0	☐1

INFORMATION SOCIO-DÉMOGRAPHIQUES

1. DATE DE NAISSANCE :	☐☐ - ☐☐☐ - ☐☐☐☐<br>Jour mois (ex. : JUN) année	
2. CONDITION DE VIE ACTUELLE<br>Avec qui habitez-vous? (cochez la ou les cases appropriées)	☐1 Seul (e)<br>☐2 Conjoint (e)<br>☐3 Enfant (s)<br>☐4 Petits-enfants	☐5 Parent (s)<br>☐6 Frère, sœur, etc.<br>☐7 Colocataire (s)<br>☐8 Pas de condition de vie stable
3. STATUT CIVIL	☐1 Célibataire<br>☐2 Marié(e) ou union libre<br>☐3 Séparé(e) ou divorcé(e)<br>☐4 Veuf (ve)	
4. TRAVAIL<br>Quel est votre statut d'emploi <u>actuel</u>? (cochez la ou les cases appropriées)	☐1 Travail à temps complet<br>☐2 Travail à temps partiel<br>☐3 À la maison<br>☐4 Étudiant(e)<br>☐5 Retraité(e)	☐6 Invalidité temporaire<br>☐7 Invalidité permanente<br>☐8 Sans emploi<br>☐9 Mise à pied<br>☐10 Bénévole<br>☐11 Autre. *Spécifiez:* ______________________
8. REVENU FAMILIAL<br>(À noter que toutes les informations recueillies dans ce questionnaire demeureront strictement confidentielles et seront traitées sur une base anonyme)	Quelle catégorie représente le mieux votre revenu familial annuel avant les déductions?	☐0 Moins de 20 000 \$<br>☐1 20 000 – 34 999 \$<br>☐2 35 000 – 49 999 \$<br>☐3 50 000 – 64 999 \$<br>☐4 65 000 – 79 999 \$<br>☐5 80 000 – 99 999 \$<br>☐6 100 000 – 119 999 \$<br>☐7 120 000\$ et plus<br>☐8 Je ne désire pas répondre

PRINCIPALE SOURCE DE REVENU

1. Veuillez indiquer votre principale source de revenu. (*Sélectionnez un choix seulement)*

- ☐1 Revenu d'un emploi ou salaire
- ☐2 Prestations d'invalidité de la CSST (Commission de la Santé et Sécurité au travail)
- ☐3 Prestations d'invalidité de la SAAQ (Société d'assurance-automobile du Québec)
- ☐4 Prestations d'invalidité de l'IVAQ (Indemnisation des victimes d'actes criminels).
- ☐5 Prestations d'invalidité de l'employeur.
- ☐6 Prestations d'invalidité d'un programme d'assurance personnelle
- ☐7 Pension de retraite (pension de retraite, sécurité de la vieillesse ou RRQ)
- ☐8 Prestation d'invalidité du régime de pension du Canada
- ☐9 Assurance-emploi
- ☐10 Assistance sociale
- ☐11 Économies personnelles ou investissements
- ☐12 Revenu assuré pour handicap sévère
- ☐13 Membre de la famille
- ☐14 Autre. *Spécifiez*: ________________________________

2. Recevez-vous, actuellement, des prestations d'invalidité de

De la CSST (Commission de la Santé et Sécurité au travail)	☐0 Non	☐1 Oui
De la SAAQ (Société d'Assurances automobile du Québec)	☐0 Non	☐1 Oui
De l'IVAC (Indemnisation des victimes d'actes criminels)	☐0 Non	☐1 Oui
De l'employeur	☐0 Non	☐1 Oui
D'un programme d'assurance personnelle	☐0 Non	☐1 Oui

3. Y a-t-il des démarches en cours pour obtenir des prestations d'invalidité

De la CSST (Commission de la Santé et Sécurité au travail)	☐0 Non	☐1 Oui
De la SAAQ (Société d'Assurances automobile du Québec)	☐0 Non	☐1 Oui
De l'IVAC (Indemnisation des victimes d'actes criminels)	☐0 Non	☐1 Oui
De l'employeur	☐0 Non	☐1 Oui
D'un programme d'assurance personnelle	☐0 Non	☐1 Oui

4. Si oui, y a-t-il un litige en cours en lien avec cette réclamation ☐0 Non ☐1 Oui ☐2 non applicable

SUIVI

Au cours des <u>3 derniers mois</u>, est-ce que :

1. Votre douleur s'est:

☐0 Considérablement détériorée
☐1 Beaucoup détériorée
☐2 Un peu détériorée
☐3 Inchangée
☐4 Un peu améliorée
☐5 Beaucoup améliorée
☐6 Considérablement améliorée

2. Votre fonctionnement s'est:

☐0 Considérablement détérioré
☐1 Beaucoup détérioré
☐2 Un peu détérioré
☐3 Inchangé
☐4 Un peu amélioré
☐5 Beaucoup amélioré
☐6 Considérablement amélioré

3. Votre qualité de vie s'est:

☐0 Considérablement détériorée
☐1 Beaucoup détériorée
☐2 Un peu détériorée
☐3 Inchangée
☐4 Un peu améliorée
☐5 Beaucoup améliorée
☐6 Considérablement améliorée

4. Veuillez indiquer sur l'échelle suivante le pourcentage de soulagement de votre douleur que vous avez observé au cours des 3 derniers mois.

0 % 10 20 30 40 50 60 70 80 90 100%

Aucun soulagement — Soulagement complet

Merci beaucoup d'avoir complété ce questionnaire.
***Veuillez vous assurer que vous avez bel et bien répondu à <u>toutes</u> les questions.
Il est très IMPORTANT pour nous qu'il n'y ait <u>pas</u> de données manquantes au moment des analyses.

Annexe 5

Indices mesurés auprès des conjoints et temps de mesure

MESURES	PHASES DE L'ÉTUDE	
	**ENTREVUE Évaluation initiale (T_0)**	**SUIVI (T_1 à T_2)**
**Caractéristiques sociodémographiques**<br>• Sexe<br>• Âge<br>• Statut civil<br>• Groupe ethnique<br>• Langue première<br>• Conditions de vie actuelles<br>• Niveau de scolarité<br>• Statut actuel d'emploi	 X<br>X<br>X<br>X<br>X<br>X<br>X<br>X	 --<br>--<br>--<br>--<br>--<br>--<br>--<br>--
**Échelle de satisfaction de la relation avec le proche**	X	X
**Qualité de vie**<br>• Santé perçue	X	X
**Échelle de perception de l'efficacité personnelle**	X	X
**Qualité de vie reliée à la santé**<br>• Qualité de vie physique et psychologique (SF-12 V2)	X	X
**Fonctionnement psychologique**<br>• Anxiété (STAI)<br>• Dépression (BDI 1)<br>• Humeur	 X<br>X<br>X	 X<br>X<br>X
**Stratégies d'adaptation**	X	X
**Expériences d'attachement amoureux**	--	X (T2 seulement)
**Échelle d'ajustement dyadique**	--	X (T2 seulement)
**Questionnaire sur la communication**	--	X (T2 seulement)

Annexe 6

Questionnaires et instruments de mesures s'adressant aux conjoints

IDENTIFICATION DU PATIENT<br>☐☐☐-☐☐☐<br>site # pt #	**QUESTIONNAIRE CONJOINT(E)**	**ÉIF**
DATE D'ENVOI<br>☐☐ - ☐☐☐ - 20☐☐<br>Jour Mois (ex: JUN) Année	DATE DE RECEPTION<br>☐☐ - ☐☐☐ - 20☐☐<br>Jour Mois (ex: JUN) Année	

Pour usage administratif seulement			
Complété:	☐ Oui	☐ Non	Si non, raison ____________________
Appel téléphonique :	☐ Oui	☐ Non	Si oui, raison ____________________

Vous trouverez dans ce document, la troisième série de questions. Ces questions sont complétées dans le cadre de l'École interactionnelle de fibromyalgie – conjoint(e).

Rappelez-vous qu'il n'y a pas de bonnes ou de mauvaises réponses. Répondez aux questions au meilleur de votre connaissance.

✐ Vous devez compléter seul ce questionnaire. Néanmoins, si un problème physique limite votre capacité à écrire, un membre de votre famille ou un ami peut vous aider à écrire vos réponses aux questions, mais il/elle ne doit en aucun moment influencer vos choix.

IMPORTANT :
ASSUREZ-VOUS DE COMPLÉTER TOUTES LES QUESTIONS

SATISFACTION DE LA RELATION AVEC LE PROCHE

	Très satisfaisante	Plutôt satisfaisante	Plutôt insatisfaisante	Très insatisfaisante
Comment qualifiez-vous la relation avec la personne fibromyalgique ou lombalgique? (cochez une seule réponse)	☐ 4	☐ 3	☐ 2	☐ 1

SANTÉ PERÇUE

	Excellente	Très bonne	Bonne	Moyenne	Mauvaise
Comparativement à d'autres personnes de votre âge, diriez-vous que votre santé est en générale : (cochez une seule réponse)	☐ 5	☐ 4	☐ 3	☐ 2	☐ 1

EFFICACITÉ PERSONNELLE

Quel degré de confiance avez-vous en votre capacité d'assumer votre rôle d'aidant auprès de votre proche?
(Encerclez une seule réponse)

0 10 20 30 40 50 60 70 80 90 100

Pas du tout confiant — Très confiant

Dans quelle mesure croyez-vous qu'il est possible de faire quelque chose pour continuer d'assumer votre rôle d'aidant?
(Encerclez une seule réponse)

0 10 20 30 40 50 60 70 80 90 100

Pas du tout possible — Tout à fait possible

Dans quelle mesure est-il important pour vous de continuer d'assumer votre rôle d'aidant?
(Encerclez une seule réponse)

0 10 20 30 40 50 60 70 80 90 100

Pas du tout important — Très important

Votre Santé et Votre Bien-être

Les questions qui suivent portent sur votre santé, telle que vous la percevez. Vos réponses permettront de suivre l'évolution de votre état de santé et de savoir dans quelle mesure vous pouvez accomplir vos activités courantes.

Pour chacune des questions suivantes, cochez la case ☒ correspondant le mieux à votre réponse.

1. En général, diriez-vous que votre santé est :

Excellente	Très bonne	Bonne	Passable	Mauvaise
☐ 1	☐ 2	☐ 3	☐ 4	☐ 5

9. Les questions suivantes portent sur les activités que vous pourriez avoir à faire au cours d'une journée normale. <u>Votre état de santé actuel vous limite-t-il</u> dans ces activités? Si oui, dans quelle mesure?

	Mon état de santé me limite beaucoup	Mon état de santé me limite un peu	Mon état de santé ne me limite pas du tout
a) Dans les <u>activités modérées</u> comme déplacer une table, passer l'aspirateur, jouer aux quilles ou au golf	☐ 1	☐ 2	☐ 3
b) Pour monter <u>plusieurs</u> étages à pied	☐ 1	☐ 2	☐ 3

10. Au cours des quatre dernières semaines, combien de fois avez-vous eu l'une ou l'autre des difficultés suivantes au travail ou dans vos autres activités quotidiennes à cause de votre état de santé physique?

	Tout le temps	La plupart du temps	Parfois	Rare-ment	Jamais
a) Avez-vous accompli moins de choses que vous l'auriez voulu?	☐ 1	☐ 2	☐ 3	☐ 4	☐ 5
b) Avez-vous été limité(e) dans la nature de vos tâches ou de vos autres activités?	☐ 1	☐ 2	☐ 3	☐ 4	☐ 5

11. Au cours des quatre dernières semaines, combien de fois avez-vous eu l'une ou l'autre des difficultés suivantes au travail ou dans vos autres activités quotidiennes à cause de l'état de votre moral (comme le fait de vous sentir déprimé(e) ou anxieux(se))?

	Tout le temps	La plupart du temps	Parfois	Rare-ment	Jamais
a) Avez-vous accompli moins de choses que vous l'auriez voulu?	☐ 1	☐ 2	☐ 3	☐ 4	☐ 5
b) Avez-vous fait votre travail ou vos autres activités avec moins de soin qu'à l'habitude?	☐ 1	☐ 2	☐ 3	☐ 4	☐ 5

5. Au cours des quatre dernières semaines, dans quelle mesure la douleur a-t-elle nui à vos activités habituelles (au travail comme à la maison)?

Pas du tout	Un peu	Moyennement	Beaucoup	Énormément
☐ 1	☐ 2	☐ 3	☐ 4	☐ 5

6. Ces questions portent sur les quatre dernières semaines. Pour chacune des questions suivantes, donnez la réponse qui s'approche le plus de la façon dont vous vous êtes senti(e). Au cours des quatre dernières semaines, combien de fois :

	Tout le temps	La plupart du temps	Parfois	Rare-ment	Jamais
a) Vous êtes-vous senti(e) calme et serein(e)?	☐ 1	☐ 2	☐ 3	☐ 4	☐ 5
b) Avez-vous eu beaucoup d'énergie?	☐ 1	☐ 2	☐ 3	☐ 4	☐ 5
c) Vous êtes-vous senti(e) triste et démoralisé(e)?	☐ 1	☐ 2	☐ 3	☐ 4	☐ 5

9. Au cours des quatre dernières semaines, combien de fois votre état physique ou moral a-t-il nui à vos activités sociales (comme visiter des amis, des parents, etc.)?

Tout le temps	La plupart du temps	Parfois	Rarement	Jamais
☐ 1	☐ 2	☐ 3	☐ 4	☐ 5

SF-12v2™ Health Survey © 1994, 2002 Health Assessment Lab, Medical Outcomes Trust and Quality Metric Incorporated. All Rights Reserved.
SF-12® is a registered trademark of Medical Outcomes Trust.
(SF-12v2 Standard, Canada (French) Version 2.0)

STAI

Veuillez lire chaque énoncé et indiquez comment vous vous sentez **maintenant**, c'est-à-dire **à ce moment précis.**

Cochez **une seule réponse** par question.

Présentement...	Pas du tout	Un peu	Modérément	Beaucoup
1. Je me sens calme.	☐ 4	☐ 3	☐ 2	☐ 1
2. Je me sens en sécurité.	☐ 4	☐ 3	☐ 2	☐ 1
3. Je suis tendu(e).	☐ 1	☐ 2	☐ 3	☐ 4
4. Je me sens surmené(e).	☐ 1	☐ 2	☐ 3	☐ 4
5. Je me sens tranquille.	☐ 4	☐ 3	☐ 2	☐ 1
6. Je me sens bouleversé(e).	☐ 1	☐ 2	☐ 3	☐ 4
7. Je suis préoccupé(e) par des malheurs possibles.	☐ 1	☐ 2	☐ 3	☐ 4
8. Je me sens comblé(e).	☐ 4	☐ 3	☐ 2	☐ 1
9. Je me sens effrayé(e).	☐ 1	☐ 2	☐ 3	☐ 4
10. Je me sens à l'aise.	☐ 4	☐ 3	☐ 2	☐ 1
11. Je me sens sûr(e) de moi.	☐ 4	☐ 3	☐ 2	☐ 1
12. Je me sens nerveux(se).	☐ 1	☐ 2	☐ 3	☐ 4
13. Je suis affolé(e).	☐ 1	☐ 2	☐ 3	☐ 4
14. Je me sens indécis(e).	☐ 1	☐ 2	☐ 3	☐ 4
15. Je suis détendu(e).	☐ 4	☐ 3	☐ 2	☐ 1
16. Je me sens satisfait(e).	☐ 4	☐ 3	☐ 2	☐ 1
17. Je suis préoccupé(e).	☐ 1	☐ 2	☐ 3	☐ 4
18. Je me sens tout(e) mêlé(e).	☐ 1	☐ 2	☐ 3	☐ 4
19. Je sens que j'ai les nerfs solides.	☐ 4	☐ 3	☐ 2	☐ 1
20. Je me sens bien.	☐ 4	☐ 3	☐ 2	☐ 1

STAI (SUITE)

Veuillez lire chaque énoncé et indiquez comment vous vous sentez **en général**, c'est-à-dire **la plus part du temps.**

Cochez **une seule réponse** par question.

En général…	Presque jamais	Quelquefois	Souvent	Presque toujours
21. Je me sens bien.	☐ 4	☐ 3	☐ 2	☐ 1
22. Je me sens nerveux(se) et agité(e).	☐ 1	☐ 2	☐ 3	☐ 4
23. Je me sens content(e) de moi-même.	☐ 4	☐ 3	☐ 2	☐ 1
24. Je voudrais être aussi heureux(se) que les autres semblent l'être.	☐ 1	☐ 2	☐ 3	☐ 4
25. J'ai l'impression d'être un(e) raté(e).	☐ 1	☐ 2	☐ 3	☐ 4
26. Je me sens reposé(e).	☐ 4	☐ 3	☐ 2	☐ 1
27. Je suis d'un grand calme.	☐ 4	☐ 3	☐ 2	☐ 1
28. Je sens que les difficultés s'accumulent au point où je n'arrive pas à les surmonter.	☐ 1	☐ 2	☐ 3	☐ 4
29. Je m'en fais trop pour des choses qui n'en valent pas vraiment la peine.	☐ 1	☐ 2	☐ 3	☐ 4
30. Je suis heureux(se).	☐ 4	☐ 3	☐ 2	☐ 1
31. J'ai des pensées troublantes.	☐ 1	☐ 2	☐ 3	☐ 4
32. Je manque de confiance en moi.	☐ 1	☐ 2	☐ 3	☐ 4
33. Je me sens en sécurité.	☐ 4	☐ 3	☐ 2	☐ 1
34. Prendre des décisions m'est facile.	☐ 4	☐ 3	☐ 2	☐ 1
35. Je sens que je ne suis pas à la hauteur de la situation.	☐ 1	☐ 2	☐ 3	☐ 4
36. Je suis satisfait(e).	☐ 4	☐ 3	☐ 2	☐ 1
37. Des idées sans importance me passent par la tête et me tracassent.	☐ 1	☐ 2	☐ 3	☐ 4
38. Je prends les désappointements tellement à cœur que je n'arrive pas à les chasser de mon esprit.	☐ 1	☐ 2	☐ 3	☐ 4
39. Je suis une personne qui a les nerfs solides.	☐ 4	☐ 3	☐ 2	☐ 1
40. Je deviens tendu(e) ou bouleversé(e) quand je songe à mes préoccupations et à mes intérêts récents.	☐ 1	☐ 2	☐ 3	☐ 4

BDI 1

Ce questionnaire contient des groupes d'énoncés. Lisez attentivement **tous les énoncés** pour chaque groupe, puis entourez le chiffre correspondant à l'énoncé qui décrit le **mieux** la façon dont vous vous êtes senti(e) au cours des **sept derniers jours**, **aujourd'hui compris.** Si plusieurs énoncés semblent convenir également bien, encerclez chacun d'eux. **Veuillez vous assurer d'avoir lu tous les énoncés de chaque groupe avant d'effectuer votre choix.**

1.	0	Je ne me sens pas triste
	1	Je me sens triste
	2	Je suis tout le temps triste et je ne peux m'en sortir
	3	Je suis si triste que je ne peux le supporter
2.	0	Je ne suis pas particulièrement découragé(e) par l'avenir
	1	Je me sens découragé(e) par l'avenir
	2	J'ai l'impression de n'avoir aucune attente dans la vie
	3	J'ai l'impression que l'avenir est sans espoir et que les choses ne peuvent s'améliorer
3.	0	Je ne me considère pas comme un(e) raté(e)
	1	J'ai l'impression d'avoir subi plus d'échecs que le commun des mortels
	2	Quand je pense à mon passé, je ne vois que des échecs
	3	J'ai l'impression d'avoir complètement échoué dans la vie
4.	0	Je retire autant de satisfaction de la vie qu'auparavant
	1	Je ne retire plus autant de satisfaction de la vie qu'auparavant
	2	Je ne retire plus de satisfaction de quoi que ce soit
	3	Tout me rend insatisfait ou m'ennuie

5.	0	Je ne me sens pas particulièrement coupable
	1	Je me sens coupable une bonne partie du temps
	2	Je me sens coupable la plupart du temps
	3	Je me sens continuellement coupable
6.	0	Je n’ai pas l’impression d’être puni(e)
	1	J’ai l’impression que je pourrais être puni(e)
	2	Je m’attends à être puni(e)
	3	J’ai l’impression d’être puni(e)
7.	0	Je n’ai pas l’impression d’être déçu(e) de moi
	1	Je suis déçu(e) de moi
	2	Je suis dégoûté(e) de moi
	3	Je me haïs
8.	0	Je n’ai pas l’impression d’être pire que quiconque
	1	Je suis critique de mes faiblesses ou de mes erreurs
	2	Je me blâme tout le temps pour mes erreurs
	3	Je me blâme pour tous les malheurs qui arrivent
9.	0	Je ne pense aucunement à me suicider
	1	J’ai parfois l’idée de me suicider, mais je n’irais pas jusqu’à passer aux actes
	2	J’aimerais me suicider
	3	J’aimerais me suicider si j’en avais l’occasion
10.	0	Je ne pleure pas plus qu’à l’ordinaire
	1	Je pleure plus qu’avant
	2	Je pleure continuellement maintenant
	3	Avant je pouvais pleurer, mais maintenant j’en suis incapable

BDI 1 (SUITE)

11.	0	Je ne suis pas plus irrité(e) maintenant qu'auparavant
	1	Je suis agacé(e) ou irrité(e) plus facilement qu'auparavant
	2	Je suis continuellement irrité(e)
	3	Je ne suis plus du tout irrité(e) par les choses qui m'irritaient auparavant
12.	0	Je n'ai pas perdu mon intérêt pour les gens
	1	Je suis moins intéressé(e) par les gens qu'autrefois
	2	J'ai perdu la plupart de mon intérêt pour les gens
	3	J'ai perdu tout intérêt pour les gens
13.	0	Je prends des décisions aussi facilement qu'avant
	1	Je remets des décisions beaucoup plus qu'auparavant
	2	J'ai beaucoup plus de difficulté à prendre des décisions qu'auparavant
	3	Je ne peux plus prendre de décisions
14.	0	Je n'ai pas l'impression que mon apparence soit pire qu'auparavant
	1	J'ai peur de paraître vieux (vieille) ou peu attrayant(e)
	2	J'ai l'impression qu'il y a des changements permanents qui me rendent peu attrayant(e)
	3	J'ai l'impression d'être laid(e)
15.	0	Je peux travailler aussi bien qu'avant
	1	Il faut que je fasse des efforts supplémentaires pour commencer quelque chose
	2	Je dois me secouer très fort pour faire quoi que ce soit
	3	Je ne peux faire aucun travail

16.	0	Je dors aussi bien que d'habitude
	1	Je ne dors aussi bien que d'habitude
	2	Je me lève une à deux heures plus tôt qu'avant et j'ai du mal à me rendormir
	3	Je me réveille plusieurs heures plus tôt qu'avant et je ne peux me rendormir
17.	0	Je ne me sens pas plus fatigué(e) qu'à l'accoutumée
	1	Je me fatigue plus facilement qu'auparavant
	2	Je me fatigue pour un rien
	3	Je suis trop fatigué(e) pour faire quoi que ce soit
18.	0	Mon appétit n'est pas pire que d'habitude
	1	Mon appétit n'est pas aussi bon qu'il l'était
	2	Mon appétit a beaucoup diminué
	3	Je n'ai plus d'appétit du tout
19.	0	Je n'ai pas perdu de poids dernièrement
	1	J'ai perdu plus de 5 livres
	2	J'ai perdu plus de 10 livres
	3	J'ai perdu plus de 15 livres

Je suis présentement un régime Oui _________ Non __________

BDI 1 (SUITE)

20.	0	Ma santé ne me préoccupe pas plus que d'habitude
	1	Je suis préoccupé(e) par des problèmes de santé comme les douleurs, les maux d'estomac ou la constipation
	2	Mon état de santé me préoccupe beaucoup et il m'est difficile de penser à autre chose
	3	Je suis tellement préoccupé(e) par mon état de santé qu'il m'est impossible de penser à autre chose
21.	0	Je n'ai remarqué récemment aucun changement dans mon intérêt pour le sexe
	1	J'ai moins de désirs sexuels qu'auparavant
	2	J'ai maintenant beaucoup moins de désirs sexuels
	3	J'ai perdu tout désir sexuel

HUMEUR

1) Jusqu'à quel point vous êtes-vous senti(e) en colère au cours des 7 derniers jours?

0 1 2 3 4 5 6 7 8 9 10

Pas du tout — Extrêmement

Trickett et Putnam, 2003

STRATÉGIES D'ADAPTATION

Veuillez lire chaque énoncé. Il s'agit de différentes stratégies que peuvent utiliser les gens afin de faire face aux difficultés qu'ils vivent. Indiquez à quelle fréquence vous avez utilisé de ces stratégies, afin de faire face à vos difficultés vis-à-vis la fibromyalgie de votre conjoint(e). Cochez **une seule réponse** par question.

	Pas utilisé	Utilisé de temps en temps	Utilisé souvent	Beaucoup utilisé
1. J'en ai parlé à quelqu'un pour en savoir plus sur cette situation.	☐ 0	☐ 1	☐ 2	☐ 3
2. J'ai espéré qu'un miracle se produise.	☐ 0	☐ 1	☐ 2	☐ 3
3. J'ai accepté mon destin, c'était peut-être juste de la malchance.	☐ 0	☐ 1	☐ 2	☐ 3
4. J'ai continué comme si rien ne s'était passé.	☐ 0	☐ 1	☐ 2	☐ 3
5. J'ai essayé de regarder les choses du bon côté.	☐ 0	☐ 1	☐ 2	☐ 3
6. J'ai accepté la sympathie et la compréhension de quelqu'un.	☐ 0	☐ 1	☐ 2	☐ 3
7. Ceci m'a donné le goût de faire quelque chose de créatif.	☐ 0	☐ 1	☐ 2	☐ 3
8. J'ai essayé de tout oublier.	☐ 0	☐ 1	☐ 2	☐ 3
9. J'ai reçu de l'aide professionnelle.	☐ 0	☐ 1	☐ 2	☐ 3
10. J'ai conçu un plan d'action et je l'ai suivi.	☐ 0	☐ 1	☐ 2	☐ 3
11. J'ai trouvé une façon d'exprimer mes émotions.	☐ 0	☐ 1	☐ 2	☐ 3
12. J'ai parlé à quelqu'un qui pouvait faire quelque chose de concret à propos du problème.	☐ 0	☐ 1	☐ 2	☐ 3
13. J'ai redécouvert ce qui était important dans la vie.	☐ 0	☐ 1	☐ 2	☐ 3
14. J'ai effectué des transformations pour que tout se termine bien.	☐ 0	☐ 1	☐ 2	☐ 3
15. J'ai demandé conseil à un parent ou à un(e) ami(e) que je respectais.	☐ 0	☐ 1	☐ 2	☐ 3
16. J'ai parlé à quelqu'un de mes sentiments.	☐ 0	☐ 1	☐ 2	☐ 3
17. J'ai tenu bon et je me suis battu(e) pour ce qui me tenait à cœur.	☐ 0	☐ 1	☐ 2	☐ 3
18. Je savais ce qui devait être fait, alors j'ai redoublé d'efforts pour que ça puisse fonctionner.	☐ 0	☐ 1	☐ 2	☐ 3
19. J'ai changé quelque chose en moi.	☐ 0	☐ 1	☐ 2	☐ 3
20. J'ai souhaité que la situation disparaisse ou prenne fin.	☐ 0	☐ 1	☐ 2	☐ 3
21. J'ai imaginé des solutions miracles.	☐ 0	☐ 1	☐ 2	☐ 3

EXPERIENCES D'ATTACHEMENT AMOUREUX

Consigne : Les énoncés suivants se rapportent à la manière dont vous vous sentez à l'intérieur de vos relations amoureuses. Nous nous intéressons à la manière dont vous vivez généralement ces relations et non seulement à ce que vous vivez dans votre relation actuelle. Répondez à chacun des énoncés en indiquant jusqu'à quel point vous êtes en accord ou en désaccord. Inscrivez le chiffre correspondant à votre choix dans l'espace réservé à cet effet selon l'échelle suivante :

Fortement en désaccord			Neutre/Partagé(e)			Fortement en accord
1	2	3	4	5	6	7

1 ______ Je préfère ne pas montrer mes sentiments profonds à mon/ma partenaire.

2 ______ Je m'inquiète à l'idée d'être abandonné(e).

3 ______ Je me sens très à l'aise lorsque je suis près de mon/ma partenaire amoureux(se).

4 ______ Je m'inquiète beaucoup au sujet de mes relations.

5 ______ Dès que mon/ma partenaire se rapproche de moi, je sens que je m'en éloigne.

6 ______ J'ai peur que mes partenaires amoureux(ses) ne soient pas autant attaché(e)s à moi que je le suis à eux(elles).

7 ______ Je deviens mal à l'aise lorsque mon/ma partenaire amoureux(se) veut être très près de moi.

8 ______ Je m'inquiète pas mal à l'idée de perdre mon/ma partenaire.

9 ______ Je ne me sens pas à l'aise de m'ouvrir à mon/ma partenaire.

10 ______ Je souhaite souvent que les sentiments de mon/ma partenaire envers moi soient aussi forts que les miens envers lui/elle.

11 ______ Je veux me rapprocher de mon/ma partenaire, mais je ne cesse de m'en éloigner.

12 ______ Je cherche souvent à me fondre entièrement avec mes partenaires amoureux(se) et ceci les fait parfois fuir.

13 ______ Je deviens nerveux(se) lorsque mes partenaires se rapprochent trop de moi.

14 ______ Je m'inquiète à l'idée de me retrouver seul(e).

15 ______ Je me sens à l'aise de partager mes pensées intimes et mes sentiments avec mon/ma partenaire.

16 ______ Mon désir d'être très près des gens les fait fuir parfois.

17 ______ J'essaie d'éviter d'être trop près de mon/ma partenaire.

18 ______ J'ai un grand besoin que mon/ma partenaire me rassure de son amour.

Fortement en désaccord			Neutre/Partagé(e)			Fortement en accord
1	2	3	4	5	6	7

19 ______ Il m'est relativement facile de me rapprocher de mon/ma partenaire.

20 ______ Parfois, je sens que je force mes partenaires à me manifester davantage leurs sentiments et leur engagement.

21 ______ Je me permets difficilement de compter sur mes partenaires amoureux(ses).

22 ______ Il ne m'arrive pas souvent de m'inquiéter d'être abandonné(e).

23 ______ Je préfère ne pas être trop près de mes partenaires amoureux(ses).

24 ______ Lorsque je n'arrive pas à faire en sorte que mon/ma partenaire s'intéresse à moi, je deviens peiné(e) ou fâché(e).

25 ______ Je dis à peu près tout à mon/ma partenaire.

26 ______ Je trouve que mes partenaires ne veulent pas se rapprocher de moi autant que je le voudrais.

27 ______ Habituellement, je discute de mes préoccupations et de mes problèmes avec mon/ma partenaire.

28 ______ Lorsque je ne vis pas une relation amoureuse, je me sens quelque peu anxieux(se) et insécure.

29 ______ Je me sens à l'aise de compter sur mes partenaires amoureux(ses).

30 ______ Je deviens frustré(e) lorsque mon/ma partenaire n'est pas là aussi souvent que je le voudrais.

31 ______ Cela ne me dérange pas de demander du réconfort, des conseils ou de l'aide à mes partenaires amoureux(ses).

32 ______ Je deviens frustré(e) si mes partenaires amoureux(ses) ne sont pas là quand j'ai besoin d'eux.

33 ______ Cela m'aide de me tourner vers mon/ma partenaire quand j'en ai besoin.

34 ______ Lorsque mes partenaires amoureux(ses) me désapprouvent, je me sens vraiment mal vis-à-vis de moi-même.

35 ______ Je me tourne vers mon/ma partenaire pour différentes raisons, entre autres pour avoir du réconfort et pour me faire rassurer.

36 ______ Je suis contrarié(e) lorsque mon/ma partenaire passe du temps loin de moi.

QEAA; Lafontaine & Lussier, 2003

ÉCHELLE D'AJUSTEMENT DYADIQUE

Ce questionnaire s'intéresse à votre perception de votre vie de couple. Il s'agit donc de votre opinion personnelle. Ne soyez pas préoccupé(e) de ce que peut ou pourrait répondre votre partenaire. Pour chaque question, indiquez votre réponse en encerclant le chiffre qui correspond à votre réponse. Assurez-vous de répondre à toutes les questions.

La plupart des gens rencontrent des problèmes dans leurs relations. Indiquez dans quelle mesure vous et votre partenaire êtes en accord ou en désaccord sur chacun des points suivants:

		Toujours d'accord	Presque toujours d'accord	Parfois d'accord	Souvent en désaccord	Presque toujours en désaccord	Toujours en désaccord
1.	Le budget familial	5	4	3	2	1	0
2.	Le domaine des sports et de la récréation	5	4	3	2	1	0
3.	Les questions religieuses	5	4	3	2	1	0
4.	Les manifestations d'affection	5	4	3	2	1	0
5.	Les amis	5	4	3	2	1	0
6.	Les relations sexuelles	5	4	3	2	1	0
7.	Les conventions sociales (se comporter de façon correcte et appropriée)	5	4	3	2	1	0
8.	La façon de voir la vie	5	4	3	2	1	0
9.	Les relations avec les parents et les beaux-parents	5	4	3	2	1	0
10.	Les buts, objectifs et choses jugées importantes	5	4	3	2	1	0
11.	La quantité de temps passé ensemble	5	4	3	2	1	0
12.	La manière de prendre des décisions importantes	5	4	3	2	1	0
13.	Les tâches à faire à la maison	5	4	3	2	1	0
14.	Les intérêts de loisir et les activités de détente	5	4	3	2	1	0
15.	Les décisions concernant le travail (métier/ profession/ carrière)	5	4	3	2	1	0

	Toujours	La plupart du temps	Plus souvent qu'autre-ment	Occasion-nellement	Rarement	Jamais
16. Est-ce qu'il vous arrive ou est-ce qu'il vous est déjà arrivé d'envisager un divorce, une séparation ou de mettre fin à votre relation actuelle?	0	1	2	3	4	5
17. Combien de fois arrive-t-il, à vous ou à votre partenaire, de quitter la maison après une chicane de ménage?	0	1	2	3	4	5
18. De façon générale, pouvez-vous dire que les choses vont bien entre vous et votre partenaire?	5	4	3	2	1	0
19. Vous confiez-vous à votre partenaire?	5	4	3	2	1	0
20. Avez-vous déjà regretté de vous être mariés (ou de vivre ensemble)?	0	1	2	3	4	5
21. Combien de fois vous arrive-t-il de vous disputer avec votre partenaire?	0	1	2	3	4	5
22. Combien de fois vous arrive-t-il, vous et votre partenaire, de vous taper sur les nerfs?	0	1	2	3	4	5

	Tous les jours	Presque chaque jour	A l'occasion	Rarement	Jamais
23. Embrassez-vous votre partenaire?	4	3	2	1	0

	Dans tous	Dans la majorité	Dans quelques-uns	Dans très peu	Dans aucun
24. Partagez-vous ensemble des intérêts extérieurs à la maison?	4	3	2	1	0

D'après vous, combien de fois les événements suivants se produisent-ils?

	Jamais	Moins qu'une fois par mois	Une ou deux fois par mois	Une ou deux fois par semaine	Une fois par jour	Plus souvent
25. Avoir un échange d'idées stimulant entre vous deux?	0	1	2	3	4	5
26. Rire ensemble?	0	1	2	3	4	5
27. Discuter calmement de quelque chose?	0	1	2	3	4	5
28. Travailler ensemble sur quelque chose?	0	1	2	3	4	5

Les couples ne sont pas toujours d'accord. Indiquez si les situations suivantes ont provoqué des différences d'opinions ou des problèmes dans votre relation au cours des dernières semaines. (Cochez oui ou non).

	Oui	Non	
29.	0	1	Être trop fatigué(e) pour avoir des relations sexuelles.
30.	0	1	Ne pas manifester son amour.

31. Les cases sur la ligne suivante correspondent à différents degrés de bonheur dans votre relation. La case centrale "heureux" correspond au degré de bonheur retrouvé dans la plupart des relations. Entourez le chiffre qui correspond le mieux au degré de bonheur de votre couple.

Extrêmement malheureux	Assez malheureux	Un peu malheureux	Heureux	Très heureux	Extrêmement heureux	Parfaitement heureux
0	1	2	3	4	5	6

32. Lequel des énoncés suivants décrit le mieux ce que vous ressentez face à l'avenir de votre relation? (Choisissez une seule réponse en entourant le chiffre correspondant.)

5 Je désire désespérément que ma relation réussisse et je ferais presque n'importe quoi pour que ça arrive.

4 Je désire énormément que ma relation réussisse et je ferai tout ce qui est en mon pouvoir pour que cela se réalise.

3 Je désire énormément que ma relation réussisse et je vais faire ma juste part pour que cela se réalise.

2 Ce serait bien si ma relation réussissait mais je ne peux pas faire beaucoup plus que ce que je fais maintenant pour y arriver.

1 Ce serait bien si cela réussissait mais je refuse de faire davantage que ce que je fais maintenant pour maintenir cette relation.

0 Ma relation ne pourra jamais réussir et je ne peux rien faire de plus pour la maintenir.

DAS; Spanier, 1976

QUESTIONNAIRE SUR LA COMMUNICATION

Consigne : Veuillez indiquer comment votre conjoint(e) et vous réagissez lorsque vous rencontrez des problèmes dans votre relation conjugale. En utilisant l'échelle de 1 (très improbable) à 9 (très probable), encerclez le chiffre qui correspond le plus à votre réponse.

LORSQU'UN PROBLÈME SURVIENT DANS NOTRE RELATION...		Très improbable								Très probable
1.	Nous évitons tous les deux d'en discuter.	1	2	3	4	5	6	7	8	9
2.	Nous essayons tous les deux d'en parler.	1	2	3	4	5	6	7	8	9
3.	Mon (ma) conjoint(e) commence la discussion alors que j'essaie de l'éviter.	1	2	3	4	5	6	7	8	9
4.	Je commence la discussion alors que mon (ma) conjoint(e) essaie de l'éviter.	1	2	3	4	5	6	7	8	9
5.	Chacun de nous exprime ses sentiments à l'autre.	1	2	3	4	5	6	7	8	9
6.	Chacun de nous blâme, accuse et critique l'autre.	1	2	3	4	5	6	7	8	9
7.	Chacun de nous suggère des solutions et des façons de s'entendre.	1	2	3	4	5	6	7	8	9
8.	Mon (ma) conjoint(e) se montre insistant(e) et fait des demandes alors que je me retire, je me tais ou je refuse de continuer la discussion.	1	2	3	4	5	6	7	8	9
9.	Je me montre insistant(e) et je fais des demandes alors que mon (ma) conjoint(e) se retire, se tait ou refuse de continuer la discussion.	1	2	3	4	5	6	7	8	9
10.	Mon (ma) conjoint(e) me fait des reproches alors que je me défends.	1	2	3	4	5	6	7	8	9
11.	Je fais des reproches à mon (ma) conjoint(e), alors qu'il (elle) se défend.	1	2	3	4	5	6	7	8	9

Communication Pattern Questionnaire-Short Form
(CPQ-SF; Christensen & Heavy, 1990)

INFORMATIONS PERSONNELLES

1. DATE DE NAISSANCE :	☐☐ - ☐☐☐ - ☐☐☐☐<br>Jour mois (ex. : JUN) année	2. Sexe ☐1 Femme<br>☐2 Homme

3. ETHNICITÉ :<br>(Cochez la ou les cases appropriées)	☐1 **Blanc** (personne ayant des ancêtres originaires d'Europe, Afrique du Nord ou Moyen-Orient)<br><br>☐2 **Noir** (personne ayant des ancêtres originaires d'Afrique ou d'ethnicité noire)<br><br>☐3 **Amérindien** (personne ayant des ancêtres originaires d'un groupe ou tribu des premières nations d'Amérique du Nord)	☐4 **Hispanique** (Mexicain, Portoricain, Cubain, Amérique centrale ou du Sud)<br><br>☐5 **Asiatique** (personne ayant des ancêtres originaires d'Orient, d'Asie, d'Inde, Iles du Pacifique, c.-à-d., Chine, Japon, Philippines, Corée, Samoa, etc.)<br><br>☐5 **Autres** : (spécifiez) ________________
4. LANGUES<br>Quelle est votre langue première?	☐1 Français<br>☐2 Anglais<br>☐3 Espagnol<br>☐4 Italien<br>☐5 Portugais	☐6 Allemand<br>☐7 Arabe<br>☐8 Chinois<br>☐9 Japonais<br>☐10 Vietnamien<br>☐11 Autres : spécifiez__________________
5. SCOLARITÉ<br>(Cochez le plus haut niveau de scolarité complété)	☐0 Aucune<br>☐1 Primaire<br>☐2 Secondaire	☐3 École technique ou CEGEP<br>☐4 Universitaire

6. CONDITION DE VIE ACTUELLE<br>Avec qui habitez-vous? (cochez la ou les cases appropriées)	☐1 Seul (e)<br>☐2 Conjoint (e)<br>☐3 Enfant (s)<br>☐4 Petits-enfants	☐5 Parent (s)<br>☐6 Frère, sœur, etc.<br>☐7 Colocataire (s)<br>☐8 Pas de condition de vie stable

7. STATUT CIVIL	☐1 Célibataire<br>☐2 Marié(e) ou union libre<br>☐3 Séparé(e) ou divorcé(e)<br>☐4 Veuf (ve)
10. TRAVAIL<br><br>Quel est votre statut d'emploi actuel?<br>(cochez la ou les cases appropriées)	☐1 Travail à temps complet<br>☐2 Travail à temps partiel<br>☐3 À la maison<br>☐4 Étudiant(e)<br>☐5 Retraité(e)<br>☐6 Invalidité temporaire<br>☐7 Invalidité permanente<br>☐8 Sans emploi<br>☐9 Mise à pied<br>☐10 Bénévole<br>☐11 Autre. *Spécifiez:* ___________________________

11. REVENU FAMILIAL<br>*(À noter que toutes les informations recueillies dans ce questionnaire demeureront strictement confidentielles et seront traitées sur une base anonyme)*	Quelle catégorie représente le mieux votre revenu familial annuel avant les déductions?	☐0 Moins de 20 000 $<br>☐1 20 000 – 34 999 $<br>☐2 35 000 – 49 999 $<br>☐3 50 000 – 64 999 $<br>☐4 65 000 – 79 999 $	☐5 80 000 – 99 999 $<br>☐6 100 000 – 119 999 $<br>☐7 120 000$ et plus<br>☐8 Je ne désire pas répondre

Merci beaucoup d'avoir complété ce questionnaire.

***Veuillez vous assurer que vous avez bel et bien répondu à toutes les questions.
Il est très IMPORTANT pour nous qu'il n'y ait pas de données manquantes au moment des analyses.
Si certaines réponses sont manquantes, nous serons dans l'obligation de vous rappeler.

Annexe 7

Questionnaires sur l’utilisation des soins de santé (volet coût)

ACCORD
Regroupement des forces pour gérer la douleur chronique

Questionnaire téléphonique sur l'utilisation de ressources de santé et la perte de productivité

Instructions :

- Avant de débuter le questionnaire téléphonique, veuillez s.v.p. avoir devant vous TOUS les médicaments que vous utilisez actuellement.
- TOUTES les informations que nous vous demanderons aujourd'hui concernent les 3 DERNIERS MOIS → *Donc depuis* ______________________ *environ!*

ID patient :

☐☐☐ - ☐☐☐

Entrevue :

■ 0 T_0
☐ 1 T_1
☐ 2 T_2
☐ 3 T_3
☐ 4 T_4

Date :

☐☐ - ☐☐☐ - 20☐☐
Jour Mois (ex: JUN) Année

Interviewer :

☐ 0 A. Lacasse
☐ 1 ____________________

UTILISATION DE RESSOURCES DE SANTÉ

Instructions :
Les questions suivantes concernent les soins de santé utilisés :

- **AU COURS DES 3 DERNIERS MOIS**
- **À CAUSE DE VOTRE DOULEUR**.
- **POUR VOTRE SANTÉ SEULEMENT** et non pour la santé de votre conjoint ou de vos enfants.

1. HOSPITALISATIONS

Au cours des 3 derniers mois, avez-vous été hospitalisé(e) à cause de votre douleur? ☐1 Oui ☐0 Non

1.1. Si oui : Combien de fois avez-vous été hospitalisé(e)? Nombre de fois : ______

1.2. Si oui : Veuillez s.v.p. me préciser les dates de ces hospitalisations, la durée, les raisons, la distance approximative entre l'hôpital et votre foyer, le moyen de transport utilisé pour vous y rendre ainsi que les frais de stationnement déboursés s'il y a lieu :

**DATE** JOUR-MOIS-ANNÉE	**DURÉE**	**RAISON(S)**	**DISTANCE DE VOTRE FOYER**	**MOYEN DE TRANSPORT** EX: VOITURE, TAXI, AUTOBUS, ETC.	**FRAIS DE STATIONNEMENT**
………………	………jours	……………………	………km	………………………..	……………$
………………	………jours	……………………	………km	………………………..	……………$
………………	………jours	……………………	………km	………………………..	……………$
………………	………jours	……………………	………km	………………………..	……………$
………………	………jours	……………………	………km	………………………..	……………$
………………	………jours	……………………	………km	………………………..	……………$
………………	………jours	……………………	………km	………………………..	……………$

2. VISITES À L'URGENCE

<u>Au cours des 3 derniers mois</u>, avez-vous eu des visites à l'urgence à cause de votre douleur? ☐1 Oui ☐0 Non

2.1. Si oui : Combien de fois avez-vous été à l'urgence? Nombre de fois : ______

2.2. Si oui : Veuillez s.v.p. me préciser les dates de ces visites, la durée, les raisons, la distance approximative entre l'urgence et votre foyer, le moyen de transport utilisé pour vous y rendre ainsi que les frais de stationnement déboursés s'il y a lieu :

**DATE** JOUR-MOIS-ANNÉE	**DURÉE**	**RAISON(S)**	**DISTANCE DE VOTRE FOYER**	**MOYEN DE TRANSPORT** EX: VOITURE, TAXI, AUTOBUS, ETC.	**FRAIS DE STATIONNEMENT**
………………	………jours	……………………	……………km	………………………..	……………$
………………	………jours	……………………	……………km	………………………..	……………$
………………	………jours	……………………	……………km	………………………..	……………$
………………	………jours	……………………	……………km	………………………..	……………$
………………	………jours	……………………	……………km	………………………..	……………$

3. VISITES CHEZ LE MÉDECIN

<u>Au cours des 3 derniers mois</u>, avez-vous consulté un médecin à cause de votre douleur? ☐1 Oui ☐0 Non

3.1. Si oui : Combien de visites avez-vous eues chez les différents médecins suivants :

- Médecin de famille (omnipraticien)	Nombre de fois : _____
- Anesthésiste	Nombre de fois : _____
- Neurochirurgien	Nombre de fois : _____
- Neurologue	Nombre de fois : _____
- Orthopédiste	Nombre de fois : _____
- Physiatre	Nombre de fois : _____
- Psychiatre	Nombre de fois : _____
- Radiologiste	Nombre de fois : _____
- Rhumatologue	Nombre de fois : _____
- Clinique de la douleur	Nombre de fois : _____
- Autre : ______________________________________	Nombre de fois : _____

3.2. Si oui : Veuillez s.v.p. me préciser les dates, le type de médecin rencontré, les raisons de ces visites, la distance approximative entre les cliniques médicales et votre foyer, le moyen de transport utilisé pour vous y rendre ainsi que les frais de stationnement déboursés s'il y a lieu :

**DATE** JOUR-MOIS-ANNÉE	**TYPE DE MÉDECIN**	**RAISON(S)**	**DISTANCE DE VOTRE FOYER**	**MOYEN DE TRANSPORT** EX : VOITURE, TAXI, AUTOBUS, ETC.	**FRAIS DE STATIONNEMENT**
…………………	……………………	……………………	……………km	………………………..	……………$
…………………	……………………	……………………	……………km	………………………..	……………$
…………………	……………………	……………………	……………km	………………………..	……………$
…………………	……………………	……………………	……………km	………………………..	……………$
…………………	……………………	……………………	……………km	………………………..	……………$

4. VISITES CHEZ UN AUTRE PROFESSIONNEL DE LA SANTÉ

<u>Au cours des 3 derniers mois</u>, avez-vous consulté un autre professionnel de la santé à cause de votre douleur? ☐1 Oui ☐0 Non

4.1. Si oui : Combien de visites avez-vous eues chez ces différents professionnels :

- Infirmière	Nombre de fois : _____
- Acupuncteur	Nombre de fois : _____
- Art-thérapeute	Nombre de fois : _____
- Chiropraticien	Nombre de fois : _____
- Ergothérapeute	Nombre de fois : _____
- Homéopathe	Nombre de fois : _____
- Hypnothérapeute	Nombre de fois : _____
- Kinésiologue / Kinésiothérapeute	Nombre de fois : _____
- Massothérapeute	Nombre de fois : _____
- Naturopathe	Nombre de fois : _____
- Ostéopathe	Nombre de fois : _____
- Pharmacien	Nombre de fois : _____
- Physiothérapeute	Nombre de fois : _____
- Psychologue	Nombre de fois : _____
- Travailleur social	Nombre de fois : _____
- Autre : ________________________________	Nombre de fois : _____

(SUITE)

4.2. Si oui : Veuillez s.v.p. me préciser les dates, le type de professionnel rencontré, les raisons de ces visites, la distance entre le lieu de la visite et votre foyer, le moyen de transport utilisé pour vous y rendre, les frais de stationnement déboursés ainsi que le remboursement de ces consultations s'il y a lieu :

**DATE**<br>JOUR-MOIS-ANNÉE	**TYPE DE PROFESSIONNEL**	**RAISON(S)**	**DISTANCE DE VOTRE FOYER**	**MOYEN DE TRANSPORT**<br>EX: VOITURE, TAXI, AUTOBUS, ETC.	**FRAIS DE STATIONNEMENT**	**MONTANT PAYÉ POUR LES SOINS**	**ALLEZ-VOUS ÊTRE REMBOURSÉ(E)?**	**SI OUI, % <u>OU</u> MONTANT REMBOURSÉ?**	**SOURCE DU REMBOURSEMENT?**<br>EX: RAMQ, ASSUREUR PRIVÉ, CSST, ETC.
……………………	………………	………………	…………km	………………………	……………$	…………$	☐Oui ☐Non	……………	…………………………
……………………	………………	………………	…………km	………………………	……………$	…………$	☐Oui ☐Non	……………	…………………………
……………………	………………	………………	…………km	………………………	……………$	…………$	☐Oui ☐Non	……………	…………………………
……………………	………………	………………	…………km	………………………	……………$	…………$	☐Oui ☐Non	……………	…………………………
……………………	………………	………………	…………km	………………………	……………$	…………$	☐Oui ☐Non	……………	…………………………
……………………	………………	………………	…………km	………………………	……………$	…………$	☐Oui ☐Non	……………	…………………………
……………………	………………	………………	…………km	………………………	……………$	…………$	☐Oui ☐Non	……………	…………………………
……………………	………………	………………	…………km	………………………	……………$	…………$	☐Oui ☐Non	……………	…………………………
……………………	………………	………………	…………km	………………………	……………$	…………$	☐Oui ☐Non	……………	…………………………
……………………	………………	………………	…………km	………………………	……………$	…………$	☐Oui ☐Non	……………	…………………………
……………………	………………	………………	…………km	………………………	……………$	…………$	☐Oui ☐Non	……………	…………………………
……………………	………………	………………	…………km	………………………	……………$	…………$	☐Oui ☐Non	……………	…………………………

INTERVENTIONS POUR VOTRE DOULEUR

---

1. <u>**Au cours des 3 derniers mois**</u>, est-ce que vous avez subi une intervention afin de soulager/traiter **VOTRE DOULEUR**?

   Exemples : Infiltration, Bloc, Chirurgie, Implant d'une pompe, Péridurale, etc.

   ☐1 Oui ☐0 Non

   1.1. Si oui, précisez :

**Date** *JOUR-MOIS-ANNÉE*	**Type d'intervention**
……………………..	………………………………………………………
……………………..	………………………………………………………
……………………..	………………………………………………………
……………………..	………………………………………………………
……………………..	………………………………………………………
……………………..	………………………………………………………
……………………..	………………………………………………………
……………………..	………………………………………………………

Injection épidurale
Infusion épidurale continue
Bloc caudal/épidurale caudale
Injection intradiscale
Bloc facettaire
Bloc branche médiane
Bloc foraminal
Bloc paravertébral
Bloc du nerf d'Arnold
Bloc d'un nerf intercostal
Bloc d'un nerf périphérique
Injection de point gachette
Injection intra-articulaire
Bloc régional intraveineux/bloc veineux
Injection de toxine botulinique
Injection intrathécale
Infusion intrathécale continue
Neurostimulation moelle épinière
Bloc sympathique (anesthésique local)
stellaire
coeliaque
lombaire
ganglion impar
Bloc sympathique (agent neurolytique)
stellaire
coeliaque
lombaire
ganglion impar
Neurolyse intrathécale
Neurolyse branche médiane
Neurolyse nerf périphérique
IDET
Coblation
Infiltration subcicatricielle
Infiltration musculaire
Perfusion zylocaïne

ACHAT DE MÉDICAMENTS EN VENTE LIBRE POUR VOTRE DOULEUR

1. **<u>Au cours des 3 derniers mois</u>**, est-ce que vous <u>ou</u> un membre de votre famille avez acheté des médicaments **en vente libre** afin de soulager/traiter **VOTRE DOULEUR**? *Exemples : Advil®, Motrin®, Tylenol®, etc.*

☐1 Oui ☐0 Non

ATTENTION : Médicaments qui ne nécessitent pas la prescription d'un médecin lors de l'achat.

1.1. Si oui, précisez :

**Date**<br>*Jour-Mois-Année*	**Nom du médicament pour votre douleur**	**Coût total incluant les taxes**	**NB d'achats depuis les 3 derniers mois**	**Allez-vous être remboursé(e)?**	**Si oui, % <u>ou</u> montant remboursé?**	**Source du remboursement?** Ex: RAMQ, assureur privé, CSST, etc.	**Utilisé actuellement?**	**Dose**	**Unités**	**Fréquence**
………………	……………………	………..$	…… x	☐Oui ☐Non	…………..	………………	☐Oui ☐Non		☐0 mg ☐1 mcg ☐2 ui	___ fois par: ☐jr ☐sem ☐mois
………………	……………………	………..$	…… x	☐Oui ☐Non	…………..	………………	☐Oui ☐Non		☐0 mg ☐1 mcg ☐2 ui	___ fois par: ☐jr ☐sem ☐mois

……………..	……………………	………..$	……<br>x	☐Oui<br>☐Non	………....	…………….	☐Oui<br>☐Non		☐0 mg ☐1 mcg ☐2 ui	___ fois par:<br>☐jr ☐sem ☐mois
……………..	……………………	………..$	……<br>x	☐Oui<br>☐Non	………....	…………….	☐Oui<br>☐Non		☐0 mg ☐1 mcg ☐2 ui	___ fois par:<br>☐jr ☐sem ☐mois
……………..	……………………	………..$	……<br>x	☐Oui<br>☐Non	………....	…………….	☐Oui<br>☐Non		☐0 mg ☐1 mcg ☐2 ui	___ fois par:<br>☐jr ☐sem ☐mois

2. [*Mis à part ce qui est mentionné ci-haut* (les nommer si applicable)] Utilisez-vous actuellement des médicaments **en vente libre** afin de soulager/traiter **VOTRE DOULEUR?**

☐1 Oui ☐0 Non

2.1. Si oui, précisez :

Date<br>*Jour-Mois-Année*	Nom du médicament pour votre douleur	Coût total incluant les taxes	NB d'achats depuis la dernière entrevue	Utilisé actuellement?	Dose	Unités	Fréquence

....	....	0 $	0 x	☐Oui<br>☐Non		☐0 mg ☐1 mcg ☐2 ui	____ fois par:<br>☐jr ☐sem ☐mois
....	....	0 $	0 x	☐Oui<br>☐Non		☐0 mg ☐1 mcg ☐2 ui	____ fois par:<br>☐jr ☐sem ☐mois
....	....	0 $	0 x	☐Oui<br>☐Non		☐0 mg ☐1 mcg ☐2 ui	____ fois par:<br>☐jr ☐sem ☐mois
....	....	0 $	0 x	☐Oui<br>☐Non		☐0 mg ☐1 mcg ☐2 ui	____ fois par:<br>☐jr ☐sem ☐mois
....	....	0 $	0 x	☐Oui<br>☐Non		☐0 mg ☐1 mcg ☐2 ui	____ fois par:<br>☐jr ☐sem ☐mois
....	....	0 $	0 x	☐Oui<br>☐Non		☐0 mg ☐1 mcg ☐2 ui	____ fois par:<br>☐jr ☐sem ☐mois

ACHAT DE MÉDICAMENTS PRESCRITS POUR VOTRE DOULEUR

1. **Au cours des 3 derniers mois**, est-ce que vous ou un membre de votre famille avez acheté des **médicaments prescrits** afin de soulager/traiter **VOTRE DOULEUR**?

   Exemples : Naproxène, Amitriptyline (Elavil®), Pregabaline (Lyrica®), etc.

   ☐1 Oui ☐0 Non

ATTENTION : Certains médicaments tels que les antidépresseurs et les anticonvulsivants peuvent êtres prescrits pour la douleur.

Si le sujet a acheté un médicament de ce genre, DEMANDER s'il souffre de dépression ou d'épilepsie?

NE PAS INSCRIRE les médicaments non reliés à la douleur.
Exemples : Antidépresseur pour la dépression uniquement OU Anticonvulsivant pour l'épilepsie uniquement.

Si le patient ne sait pas si son médicament est pour la dépression ou la douleur l'INSCRIRE.

1.1. Si oui, précisez :

**Date** *Jour-Mois-Année*	**Nom du médicament pour votre douleur**	**NB d'achats depuis les 3 derniers mois**	**Allez-vous être remboursé(e)?**	**Si oui, % ou montant remboursé?**	**Source du remboursement?** Ex: RAMQ, assureur privé, CSST, etc.	**Utilisé actuellement?**	**Dose**	**Unités**	**Fréquence**
………………	……………………..	………… **x**	☐Oui ☐Non	…………..	………………	☐Oui ☐Non		☐0 mg ☐1 mcg ☐2 ui	___ fois par: ☐jr ☐sem ☐mois
………………	……………………..	………… **x**	☐Oui ☐Non	…………..	………………	☐Oui ☐Non		☐0 mg ☐1 mcg ☐2 ui	___ fois par: ☐jr ☐sem ☐mois
………………	……………………..	………… **x**	☐Oui ☐Non	…………..	………………	☐Oui ☐Non		☐0 mg ☐1 mcg ☐2 ui	___ fois par: ☐jr ☐sem ☐mois

(suite)

**Date**<br>*Jour-Mois-Année*	**Nom du médicament pour votre douleur**	**NB d'achats depuis les 3 derniers mois**	**Allez-vous être remboursé(e)?**	**Si oui, % ou montant remboursé?**	**Source du remboursement?** Ex: RAMQ, assureur privé, CSST, etc.	**Utilisé actuellement?**	**Dose**	**Unités**	**Fréquence**
…………….	……………………	……….. x	☐Oui<br>☐Non	…………	…………….	☐Oui<br>☐Non		☐0 mg ☐1 mcg ☐2 ui	___ fois par:<br>☐jr ☐sem ☐mois
…………….	……………………	……….. x	☐Oui<br>☐Non	…………	…………….	☐Oui<br>☐Non		☐0 mg ☐1 mcg ☐2 ui	___ fois par:<br>☐jr ☐sem ☐mois
…………….	……………………	……….. x	☐Oui<br>☐Non	…………	…………….	☐Oui<br>☐Non		☐0 mg ☐1 mcg ☐2 ui	___ fois par:<br>☐jr ☐sem ☐mois
…………….	……………………	……….. x	☐Oui<br>☐Non	…………	…………….	☐Oui<br>☐Non		☐0 mg ☐1 mcg ☐2 ui	___ fois par:<br>☐jr ☐sem ☐mois
…………….	……………………	……….. x	☐Oui<br>☐Non	…………	…………….	☐Oui<br>☐Non		☐0 mg ☐1 mcg ☐2 ui	___ fois par:<br>☐jr ☐sem ☐mois
…………….	……………………	……….. x	☐Oui<br>☐Non	…………	…………….	☐Oui<br>☐Non		☐0 mg ☐1 mcg ☐2 ui	___ fois par:<br>☐jr ☐sem ☐mois
…………….	……………………	……….. x	☐Oui<br>☐Non	…………	…………….	☐Oui<br>☐Non		☐0 mg ☐1 mcg ☐2 ui	___ fois par:<br>☐jr ☐sem ☐mois

………………	……………………	………… **x**	☐Oui<br>☐Non	………….	………………	☐Oui<br>☐Non		☐$_0$ mg ☐$_1$ mcg ☐$_2$ ui	___ fois par:<br>☐jr ☐sem ☐mois

1.2. Si oui, <u>au cours des 3 derniers mois</u>, est-ce que vous <u>ou</u> un membre de votre famille avez acheté des **médicaments prescrits <u>afin de soulager les effets indésirables des médicaments pour</u> VOTRE DOULEUR**?

Exemples : Antiacides, laxatifs… : Senokot®, Nexium®, Prevacid®, Losec®, Pantoloc®, Pepcid®, Zantac®, etc.

ATTENTION : Ne pas inscrire les médicaments <u>non reliés</u> à la prise de médicaments pour la douleur.

Exemple : Laxatif pour un problème de constipation chronique.

**Date** *Jour-Mois-Année*	**Nom du médicament pour votre douleur**	**NB d'achats depuis les 3 derniers mois**	**Allez-vous être remboursé(e)?**	**Si oui, % <u>ou</u> montant remboursé?**	**Source du remboursement?** Ex: RAMQ, assureur privé, CSST, etc.	**Utilisé actuellement?**	**Dose**	**Unités**	**Fréquence**
……………	……………………	………… **x**	☐Oui ☐Non	…………	……………	☐Oui ☐Non		☐0 mg ☐1 mcg ☐2 ui	___ fois par: ☐jr ☐sem ☐mois
……………	……………………	………… **x**	☐Oui ☐Non	…………	……………	☐Oui ☐Non		☐0 mg ☐1 mcg ☐2 ui	___ fois par: ☐jr ☐sem ☐mois
……………	……………………	………… **x**	☐Oui ☐Non	…………	……………	☐Oui ☐Non		☐0 mg ☐1 mcg ☐2 ui	___ fois par: ☐jr ☐sem ☐mois
……………	……………………	………… **x**	☐Oui ☐Non	…………	……………	☐Oui ☐Non		☐0 mg ☐1 mcg ☐2 ui	___ fois par: ☐jr ☐sem ☐mois

………………	……………………	………… **x**	☐Oui<br>☐Non	…………..	………………	☐Oui<br>☐Non		☐0 mg ☐1 mcg ☐2 ui	____ fois par:<br>☐jr ☐sem ☐mois

ACHAT DE PRODUITS NATURELS POUR VOTRE DOULEUR

1. **Au cours des 3 derniers mois**, est-ce que vous ou un membre de votre famille avez acheté des **produits naturels** afin de soulager/traiter **VOTRE DOULEUR**?

   Exemples : Cannabis, Haschich, Herbes, Produits homéopathiques, Baume du tigre® etc.

   ☐1 Oui ☐0 Non

   1.1. Si oui, précisez :

**Date**<br>Jour-Mois-Année	**Nom du produit naturel pour votre douleur**	**Coût total incluant les taxes**
………………	………………………………………..	……………$
………………	………………………………………..	……………$
………………	………………………………………..	……………$
………………	………………………………………..	……………$
………………	………………………………………..	……………$
………………	………………………………………..	……………$
………………	………………………………………..	……………$
………………	………………………………………..	……………$
………………	………………………………………..	……………$
………………	………………………………………..	……………$

2. [*Mis à part ce qui est mentionné ci-haut* (les nommer si applicable)] Utilisez-vous actuellement des **produits naturels** afin de soulager/traiter **VOTRE DOULEUR?**

☐1 Oui ☐0 Non

2.1. Si oui, précisez :

**Date** *Jour-Mois-Année*	**Nom du produit naturel pour votre douleur**	**Coût total incluant les taxes**	**Utilisé actuellement?**
………………	………………………………………	**0 $**	☐Oui ☐Non
………………	………………………………………	**0 $**	☐Oui ☐Non
………………	………………………………………	**0 $**	☐Oui ☐Non
………………	………………………………………	**0 $**	☐Oui ☐Non
………………	………………………………………	**0 $**	☐Oui ☐Non
………………	………………………………………	**0 $**	☐Oui ☐Non

ACHAT DE FOURNITURES POUR VOTRE DOULEUR

1. **<u>Au cours des 3 derniers mois</u>**, est-ce que vous <u>ou</u> un membre de votre famille avez acheté des **fournitures** afin de soulager/traiter **VOTRE DOULEUR**?

   Exemples : Support lombaire, Béquilles, Chaise roulante, Sac Magique® etc.
   ☐1 Oui ☐0 Non

   1.1. Si oui, précisez :

**Date**<br>Jour-Mois-Année	**Nom de la fourniture pour votre douleur**	**Coût total incluant les taxes**	**Allez-vous être remboursé(e)?**	**Si oui, % <u>ou</u> montant remboursé?**	**Source du remboursement?**<br>Ex: RAMQ, assureur privé, CSST, etc.

………………	………………………..	…………..$	☐Oui ☐Non	………………..	………………………..
………………	………………………..	…………..$	☐Oui ☐Non	………………..	………………………..
………………	………………………..	…………..$	☐Oui ☐Non	………………..	………………………..
………………	………………………..	…………..$	☐Oui ☐Non	………………..	………………………..
………………	………………………..	…………..$	☐Oui ☐Non	………………..	………………………..
………………	………………………..	…………..$	☐Oui ☐Non	………………..	………………………..
………………	………………………..	…………..$	☐Oui ☐Non	………………..	………………………..
………………	………………………..	…………..$	☐Oui ☐Non	………………..	………………………..
………………	………………………..	…………..$	☐Oui ☐Non	………………..	………………………..
………………	………………………..	…………..$	☐Oui ☐Non	………………..	………………………..

EFFETS INDÉSIRABLES

1. Maintenant, nous allons discuter des effets indésirables que vous auriez pu avoir **À CAUSE DE VOTRE TRAITEMENT ACTUEL CONTRE LA DOULEUR** (médicaments analgésiques ou produits naturels)?

☐0 Non applicable, car aucun médicament, cannabis ou produit naturel n'a été utilisé pour soulager la douleur.

ATTENTION : Ne pas inscrire les effets indésirables non reliés au traitement de la douleur.

Exemples : Gain de poids relié à la cessation tabagique,
Vomissements reliés à une intoxication alimentaire.

a) Vertige / étourdissement?	☐0 aucun	☐1 léger	☐2 modéré	☐3 sévère
b) Somnolence ?	☐0 aucun	☐1 léger	☐2 modéré	☐3 sévère
c) Confusion ?	☐0 aucun	☐1 léger	☐2 modéré	☐3 sévère
d) Nausée ?	☐0 aucun	☐1 léger	☐2 modéré	☐3 sévère
e) Vomissement?	☐0 aucun	☐1 léger	☐2 modéré	☐3 sévère
f) Perte de mémoire ?	☐0 aucun	☐1 léger	☐2 modéré	☐3 sévère
g) Bouche sèche ?	☐0 aucun	☐1 léger	☐2 modéré	☐3 sévère
h) Démangeaison ?	☐0 aucun	☐1 léger	☐2 modéré	☐3 sévère
i) Inconfort abdominal ?	☐0 aucun	☐1 léger	☐2 modéré	☐3 sévère
j) Constipation ?	☐0 aucun	☐1 léger	☐2 modéré	☐3 sévère
k) Ralentissement du débit urinaire ?	☐0 aucun	☐1 léger	☐2 modéré	☐3 sévère
l) Fatigue ?	☐0 aucun	☐1 léger	☐2 modéré	☐3 sévère
m) Insomnie ?	☐0 aucun	☐1 léger	☐2 modéré	☐3 sévère
n) Enflure ?	☐0 aucun	☐1 léger	☐2 modéré	☐3 sévère
o) Gain de poids ?	☐0 aucun	☐1 léger	☐2 modéré	☐3 sévère
p) Vision brouillée ?	☐0 aucun	☐1 léger	☐2 modéré	☐3 sévère
q) Baisse de libido?	☐0 aucun	☐1 léger	☐2 modéré	☐3 sévère

Toujours en raison de votre traitement contre la douleur!

r) Hallucinations?	☐0 aucun	☐1 léger	☐2 modéré	☐3sévère
s) Cauchemars?	☐0 aucun	☐1 léger	☐2 modéré	☐3sévère
t) Autres : (spécifiez) __________	☐0 aucun	☐1 léger	☐2 modéré	☐3 sévère
u) Autres : (spécifiez) __________	☐0 aucun	☐1 léger	☐2 modéré	☐3 sévère

AIDE DOMESTIQUE RÉMUNÉRÉ

1. **<u>Au cours des 3 derniers mois</u>,** avez-vous rémunéré d'autres personnes (membre de la famille, ami, voisin, etc.) pour vous aider avec les tâches ménagères ou familiales **À CAUSE DE VOTRE DOULEUR?**

Même si cette rémunération n'est pas déclarée, soyez certains que cette information demeurera confidentielle!

OUI	NON
☐1	☐0

1.1. Le cas échéant, veuillez s.v.p. me préciser les dates, le nombre d'heures d'aide avec les tâches ménagères ou familiales, le montant total payé par vous ainsi que le type d'aide apporté :

DATE<br>*JOUR-MOIS-ANNÉE*	TOTAL D'HEURES	MONTANT TOTAL PAYÉ	TYPE D'AIDE APPORTÉE
………………	……………hr	……………$	………………………
………………	……………hr	……………$	………………………
………………	……………hr	……………$	………………………
………………	……………hr	……………$	………………………
………………	……………hr	……………$	………………………
………………	……………hr	……………$	………………………
………………	……………hr	……………$	………………………
………………	……………hr	……………$	………………………
………………	……………hr	……………$	………………………
………………	……………hr	……………$	………………………

AUTRES FRAIS

1. Mis à part toutes les informations que vous avez fournies jusqu'à présent, avez-vous eu <u>d'autres frais</u> à assumer pour soigner **VOTRE DOULEUR** ou résultant de votre douleur **<u>au cours des 3 derniers mois</u>**? ☐1 Oui ☐0 Non

Exemples : Dépenses payées par vos proches, rénovations, etc.

1.1. Si oui, précisez :

**DATE** *JOUR-MOIS-ANNÉE*	**TYPE DE FRAIS**	**MONTANT TOTAL PAYÉ**
………………	……………………………………………	……………$
………………	……………………………………………	……………$
………………	……………………………………………	……………$
………………	……………………………………………	……………$
………………	……………………………………………	……………$
………………	……………………………………………	……………$

PERTE DE PRODUCTIVITÉ

1. <u>Au cours des 3 derniers mois</u>, aviez-vous un travail rémunéré?

☐1 Oui ☐0 Non

SI « **OUI**» ADMINISTRER LA SECTION **A** ET **B**

SI « **NON** » ADMINISTRER LA SECTION **B SEULEMENT**

SECTION A

1.1. <u>Au cours des 3 derniers mois</u>, combien de jours avez-vous été **absent(e**) de votre travail rémunéré en raison d'une **VISITE MÉDICALE RELIÉE AU TRAITEMENT DE VOTRE DOULEUR**?
Incluant la participation à une école interactionnelle, les rendez-vous à la clinique de la douleur, les hospitalisations, les visites à l'urgence, les visites chez un médecin, les visites chez un professionnel de la santé et les visites à la pharmacie, etc.

Nombre de journées: ___________

1.2. <u>Au cours des 3 derniers mois,</u> combien de jours avez-vous été **absent(e)** de votre travail rémunéré **À CAUSE DE VOTRE DOULEUR**?
Exemples : Vous aviez trop mal pour travailler, vous étiez inconfortable, vous êtes arrivé en retard, etc.

Nombre de journées: ___________

1.3. En moyenne, <u>combien de jours par semaine</u> êtes-vous normalement supposé travaillé?

Nombre de journées: ___________ /semaine

1 journée = 7.5 ou 8 heures

1.4. Grâce à votre travail rémunéré, quel était **VOTRE** revenu annuel avant impôts au cours des 3 derniers mois?

☐0 Moins de 10 000 $
☐1 Entre 10 000 $ et 19 999 $
☐2 Entre 20 000 $ et 29 999 $
☐3 Entre 30 000 $ et 39 999 $
☐4 Entre 40 000 $ et 49 999 $
☐5 Entre 50 000 $ et 59 999 $
☐6 Entre 60 000 $ et 69 999 $
☐7 Entre 70 000 $ et 79 999 $
☐8 Entre 80 000 $ et 89 000 $
☐9 Entre 90 000 $ et 99 000 $
☐10 100 000 $ et plus ☐ Ne désire pas répondre

SECTION B

Les questions suivantes portent sur le travail au foyer NON RÉMUNÉRÉ, incluant les tâches domestiques et celles liées aux soins des enfants et des proches.

1.5. Au cours des 3 derniers mois, combien de jours avez-vous été obligé(e) de cesser votre **travail au foyer non rémunéré** en raison d'une **VISITE MÉDICALE RELIÉE AU TRAITEMENT DE VOTRE DOULEUR**?

Incluant la participation à une école interactionnelle, les rendez-vous à la clinique de la douleur, les hospitalisations, les visites à l'urgence, les visites chez un médecin, les visites chez un professionnel de la santé et les visites à la pharmacie, etc.

Nombre de journées: ___________

1.6. Au cours des 3 derniers mois, combien de jours avez-vous été obligé(e) de cesser votre **travail au foyer non rémunéré À CAUSE DE VOTRE DOULEUR**?

Exemples : Vous aviez trop mal pour effectuer vos tâches, vous étiez inconfortable etc.

Nombre de journées: ___________

PROPENSITÉ À PAYER POUR L'INTERVENTION

Pour finir, une petite mise en situation…
Nous allons faire comme si vous aviez à payer pour participer aux Écoles Interactionnelles offertes dans votre région. Bien sûr, dans la réalité, votre participation présente ou future aux Écoles Interactionnelles est entièrement gratuite!

Compte tenu de vos symptômes douloureux, quel montant maximum seriez-vous prêt à payer pour participer à l'École interactionnelle de :

_________$

LE QUESTIONNAIRE SE TERMINE ICI.

EST-CE QUE VOUS AVEZ DES QUESTIONS?

SI CE N'EST PAS DÉJÀ FAIT, JE VOUS INVITE ÉGALEMENT À RETOURNER VOTRE QUESTIONNAIRE PAPIER

JE VOUS REMERCIE BEAUCOUP DE VOTRE PARTICIPATION!

NOUS NOUS REPARLERONS DANS ENVIRON 2 MOIS POUR UNE ENTREVUE SIMILAIRE.

Annexe 8

Formulaire d'information et de consentement à la recherche
pour le participant

CENTRE HOSPITALIER UNIVERSITAIRE DE SHERBROOKE
Hôpital Fleurimont, 3001, 12e Avenue Nord, Fleurimont (Québec) J1H 5N4
Hôtel-Dieu, 580, rue Bowen Sud, Sherbrooke (Québec) J1G 2E8
Téléphone : (819) 346-1110

FORMULAIRE D'INFORMATION ET DE CONSENTEMENT À LA RECHERCHE

Titre du projet: **DÉVELOPPEMENT, IMPLANTATION ET ÉVALUATION D'INTERVENTIONS DE TYPE «ÉCOLE INTERACTIONNELLE» ADAPTÉES À DES PERSONNES SOUFFRANT DE FIBROMYALGIE OU DE LOMBALGIE - PARTICIPANT**

Numéro et date du projet ***Programme Accord – Volet 3A – Partie 1***

Organisme subventionnaire Instituts de recherche en santé du Canada (IRSC)

Chercheurs principaux:
Patricia Bourgault, inf. Ph.D. École des sciences infirmières, FMSS, Université de Sherbrooke
Serge Marchand, Ph.D. Service de neurochirurgie, FMSS, Université de Sherbrooke

Chercheurs associés :
Isabelle Gaumond, Ph.D. Université du Québec en Abitibi-Témiscamingue
Jacques Charest, Ph.D. Université du Québec en Abitibi-Témiscamingue
Anaïs Lacasse-Morris, Ph.D. Université du Québec en Abitibi-Témiscamingue
Manon Choinière, Ph.D., Centre de recherche du Centre hospitalier de l'Université de Montréal,
Dominique Dion, M.D., M.Sc. Centre de recherche du Centre hospitalier de l'Université de Montréal

Collaborateurs:
Christian Cloutier, M.D., Service de neurochirurgie, Centre hospitalier universitaire de Sherbrooke
Sylvie Lamoureux, M.A.Ps., Clinique multidisciplinaire de douleur chronique, Centre hospitalier universitaire de Sherbrooke

Étudiante :
Émilie Lagueux, Erg., programme de maîtrise en sciences cliniques

---

Pour de plus amples renseignements, vous pouvez contacter :
Patricia Bourgault au 819 346-1110 poste 12885 du lundi au vendredi de 8h à 17h

En cas d'urgence médicale, veillez contacter votre médecin traitant ou vous présenter à l'urgence.

CÉR chez l'humain du CHUS
APPROUVÉ 26 nov 09

Nous sollicitons votre participation à un projet de recherche. Cependant, avant d'accepter de participer à ce projet, veuillez prendre le temps de lire, de comprendre et de considérer attentivement les renseignements qui suivent. Si vous acceptez de participer au projet de recherche, vous devrez signer le consentement à la fin du présent document et nous vous en remettrons une copie pour vos dossiers.

Ce formulaire d'information et de consentement vous explique le but de ce projet de recherche, les procédures, les avantages, les risques et inconvénients, de même que les personnes avec qui communiquer au besoin. Il peut contenir des mots que vous ne comprenez pas. Nous vous invitons à poser toutes les questions nécessaires au chercheur responsable du projet ou aux autres personnes affectées au projet de recherche et à leur demander de vous expliquer tout mot ou renseignement qui n'est pas clair.

NATURE ET OBJECTIFS DU PROJET DE RECHERCHE

Il existe différentes possibilités de traitement pour la fibromyalgie (FM) et la lombalgie (maux de dos) (LB). Plusieurs de ces traitements n'atteignent pas les objectifs visés notamment face à la réduction de la douleur, l'amélioration du sommeil et de la qualité de vie. Les traitements sont le plus souvent à base de médicaments associés ou non à une approche physique ou psychologique. Il est cependant reconnu scientifiquement que la combinaison de différentes approches donne les meilleurs résultats surtout si l'intervention est interdisciplinaire (divers professionnels de la santé travaillant au même objectif). Le but de la présente étude est d'évaluer l'efficacité à court et à long terme d'interventions interdisciplinaires de type « École interactionnelle de fibromyalgie (ÉIF) et de lombalgie (ÉIL) » pour réduire la sévérité de la douleur chez des personnes souffrant de fibromyalgie ou de lombalgie. Parallèlement, nous procéderons à une évaluation de coûts reliés à la douleur et à l'utilisation des ressources de santé, ce qui permettra d'obtenir des données préliminaires sur l'impact des ÉIF et ÉIL.

Cette étude s'adresse aux personnes de 18 et plus, souffrant de symptômes de fibromyalgie ou de lombalgie depuis au moins six mois (le diagnostic sera confirmé par un médecin lors de l'évaluation initiale). Pour participer à l'étude, vous devez également répondre aux critères suivants : Rapporter une douleur d'intensité moyenne plus grande ou égale à 4 sur 10 au cours des 7 jours précédant l'évaluation initiale; être intéressé et motivé par le type d'intervention proposé; accepter de participer à 8 rencontres étalées sur une période de 11 semaines et à une visite de relance (6 mois plus tard). Vous devez également accepter de ne pas introduire de nouvelle(s) classe(s) de médicaments contre la douleur pour la durée de l'École (11 semaines). Vous pouvez continuer d'utiliser les analgésiques et/ou co-analgésiques ou les diminuer et les cesser durant la période couverte pas l'École mais ceci doit être spontané. Le but de l'École de FM n'est cependant PAS d'amener les patients à diminuer ou cesser leurs médicaments contre la douleur mais ceci peut se produire lorsque les symptômes sont mieux gérés. Vous acceptez également de ne pas introduire de nouvelle(s) modalité(s) thérapeutique(s) pour la durée de l'École (11 semaines) (ex. :bloc anesthésique, acupuncture, physiothérapie, thérapie de groupe, hypnose, etc).

CÉR chez l'humain du CHUS
APPROUVÉ 26 nov 09 [illegible]

DÉROULEMENT DU PROJET DE RECHERCHE

Cette étude se déroulera à l'École de réadaptation de l'Université de Sherbrooke (bâtiment annexé au Centre hospitalier universitaire de Sherbrooke à Fleurimont) à l'automne 2009 et au printemps 2010 (ÉIF) et au printemps 2010 et à l'automne 2010 (ÉIL). Votre participation implique les activités suivantes : une rencontre d'évaluation initiale et la présence aux 9 séances des Écoles interactionnelles et la réponse à des questionnaires.

L'étude comporte deux groupes de participants : un groupe appelé « intervention » composé de 16 personnes souffrant de fibromyalgie et 16 de lombalgie et un groupe appelé « témoin » également composé de 16 personnes souffrant de fibromyalgie et 16 de lombalgie. C'est le hasard qui déterminera à quel groupe vous serez assigné. La détermination des groupes se fera suite à la rencontre d'évaluation initiale. Le tableau ci-dessous précise les dates des différentes rencontres pour les deux groupes.

Déroulement de l'étude et collecte des données

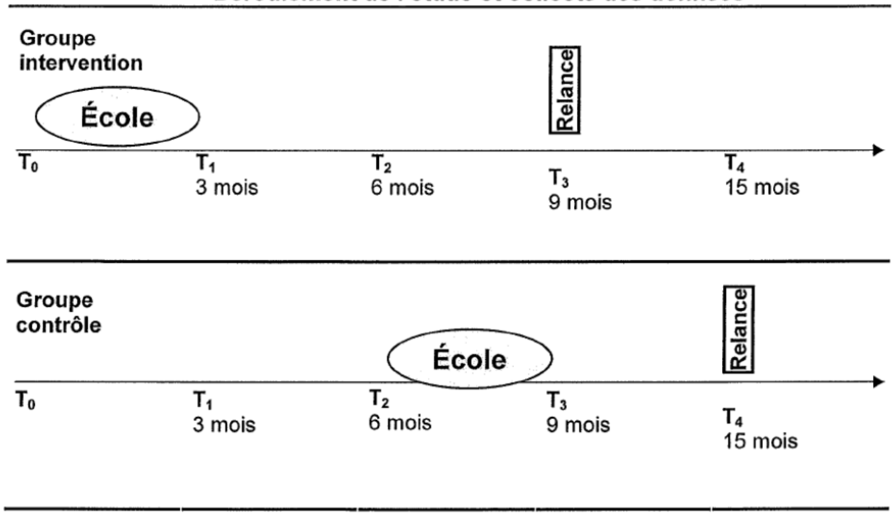

Évaluation initiale : Un premier rendez-vous sera fixé au site de l'étude afin de confirmer le diagnostic de FM ou de LB (par le médecin collaborateur à l'étude) ainsi que pour vérifier votre intérêt pour apprendre à mieux gérer votre douleur (objectif de l'intervention) et la disponibilité de votre conjoint pour participer à l'étude (pas obligatoire). Des données sociodémographiques seront recueillies (sexe, année de naissance, ethnicité, langue première, scolarité, condition de vie actuelle, statut civil, statut d'emploi, etc.) à ce moment.

CÉR chez l'humain du CHUS
APPROUVÉ 26 [illegible]

Lors de l'évaluation initiale et des évaluations suivantes (3, 6, 9 et 15 mois), nous vous demanderons (groupe intervention et groupe témoin) de compléter les questionnaires listés ci-dessous. Il vous prendra environ 60 minutes pour les compléter. Vous pouvez demander de les compléter à domicile dans ce cas, une enveloppe pré-affranchie vous sera remise.

Questionnaires

Pour tous les participants

- Caractéristiques sociodémographiques (statut civil, condition de vie actuelle, statut d'emploi)
- Caractéristiques de la douleur
- Qualité du sommeil
- Consommation tabac, alcool et drogue
- Traitements pharmacologiques et non pharmacologiques contre la douleur
- Stratégies d'ajustement face à la douleur
- Niveau de dépression
- Qualité de vie reliée à la santé
- Attentes du patient face à l'intervention
- Impression globale de changement
- Coûts reliés à la douleur et à l'utilisation des ressources de santé*
  *Pour ce questionnaire, vous recevrez un appel téléphonique de Mme Anaïs Lacasse-Morris qui dirige cette partie de l'étude.

Pour les participants des ÉIF (Fibromyalgie)

- Impact de la fibromyalgie

Pour les participants des EIL (Lombalgie)
Incapacités engendrées par la lombalgie

Groupe Intervention – Écoles interactionnelles : Ces Écoles comportent des séances d'information sur votre problème de santé (fibromyalgie ou lombalgie) et ses principaux symptômes, des prescriptions de tâches à effectuer, des exercices physiques individualisés, des techniques de relaxation et des stratégies de gestion du stress. Le détail des rencontres est présenté à la fin de ce document. Les rencontres se font en groupe de 6 à 8 personnes. Chaque rencontre dure environ 2 heures 30 minutes. Suite aux rencontres, vous serez invité à remplir un carnet de route. Le carnet de route est votre outil afin de suivre vos progrès. Lors de la première rencontre, vous compléterez un journal de la douleur. Ces documents vous appartiennent et vous pourrez les conserver.

Toutes les rencontres en groupe seront filmées et vous pourrez être identifié. Le visionnement des enregistrements est réservé exclusivement aux participants qui seront absents à une rencontre de même qu'aux membres de l'équipe de recherche et à l'équipe des intervenants afin de s'assurer que les écoles sont menées de façon adéquate et uniforme. Les enregistrements vidéo seront conservés dans un tiroir fermé à clef dans le bureau de Patricia Bourgault, à l'École des sciences infirmières de

CÉR chez l'humain du CHUS
APPROUVÉ 26 [illegible]

l'Université de Sherbrooke. Il est également possible que certains enregistrements servent à la formation de futurs intervenants qui appliqueront les ÉI dans d'autres centres au Québec. Nous vous demanderons votre permission à la fin des ÉI pour utiliser ces enregistrements et vous serez invité à signer un document à cet effet. Précisons néanmoins que ces enregistrements ne circuleront pas. Ils resteront en possession des chercheurs (PB et SM) qui les utiliseront lors de formations. En aucun temps ces enregistrements ne serviront à d'autres objets que ceux mentionnés précédemment.

Groupe Témoin : Si vous avez été assigné au groupe témoin, votre participation à l'École interactionnelle sera retardée de 6 mois mais elle sera en tout point identique à celle décrite précédemment. Le décalage nous permet de mesurer l'effet du temps sur votre condition i.e est-ce que l'amélioration est due à l'intervention ou si simplement votre condition peut s'améliorer sans celle-ci.

Afin d'assurer une uniformité des ÉI, une supervision sera effectuée lors des quatre premières séances. Patricia Bourgault assurera cette supervision qui s'adresse aux intervenants.

COLLABORATION DU SUJET DE RECHERCHE

Si vous êtes assigné au groupe Intervention, votre participation implique que vous assistiez à toutes les rencontres et compléterez les questionnaires (étalés sur une période de 15 mois) de même que le carnet de route et le journal de la douleur décrits précédemment. Vous devrez également compléter ces mêmes questionnaires aux moments inscrits au tableau. Enfin, votre adhésion à l'École demande que vous appliquiez les stratégies proposées telles la routine motrice, la marche, la relaxation ainsi que d'autres stratégies touchant le sommeil et la nutrition.

Si vous êtes assigné au groupe Témoin, nous vous demanderons de compléter des questionnaires dans 3 mois et dans 6 mois. Puis, vous participerez à l'École interactionnelle selon exactement la même formule que celle décrite précédemment.

RISQUES POUVANT DÉCOULER DE LA PARTICIPATION DU SUJET AU PROJET DE RECHERCHE

Il est possible que vous ressentiez de la douleur lorsque vous débuterez les activités physiques inclues dans l'École interactionnelle ou lorsque que vous reprendrez vos activités physiques quotidiennes. Cependant, ces douleur devraient s'estomper à mesure que votre corps se réhabituera à bouger. Si jamais ces douleurs persistent, vous pourrez en parler à l'équipe d'intervenants qui verront avec vous les raisons de ces douleurs (e.g. mouvement non-adapté à votre condition) et au besoin, vous référeront au médecin collaborateur à cette étude ou à votre médecin traitant. Si vous éprouvez des difficultés au plan psychologique, les intervenants des ÉI vous référeront, si vous le souhaitez, à un psychologue ne participant pas à l'intervention. Également si les questionnaires démontrent une humeur dépressive importante, nous vous téléphonerons et au besoin contacterons votre médecin traitant.

CÉR chez l'humain du CHUS
APPROUVÉ 26 nov 09 VB

Les chercheurs responsables de l'étude évalueront constamment l'intervention qui vous est offerte. Si jamais, les connaissances acquises démontraient que l'intervention n'est plus adéquate pour tous ou pour vous en particulier, ils cesseront l'étude sans tarder et s'assureront que votre suivi est adéquat. Pour ce faire, nous vous demanderons d'identifier votre médecin traitant afin que nous puissions, si vous l'acceptez, l'aviser de votre participation à l'intervention et au besoin de sa cessation.

INCONVÉNIENTS POUVANT DÉCOULER DE LA PARTICIPATION DU SUJET AU PROJET DE RECHERCHE

Si vous êtes assigné au groupe Témoin, vous verrez votre participation à l'École interactionnelle retardée, ce qui pourrait constituer un inconvénient. Toutefois, nous pourrons vous assurer que l'École sera en tout point identique à celle que vous auriez reçue si vous aviez été assigné au Groupe Intervention.

Le fait de répondre à des questionnaires ne vous causera aucun inconvénient sinon le temps nécessaire pour les compléter. Il en va de même pour les rencontres qui vous demanderont environ 2 heures 30 par semaine (présence). De plus, l'intervention ou le fait de répondre aux questionnaires pourrait faire ressortir des sentiments de tristesse.

AVANTAGES POUVANT DÉCOULER DE LA PARTICIPATION DU SUJET AU PROJET DE RECHERCHE

Il se peut que vous retiriez un bénéfice personnel de votre participation à ce projet de recherche, mais nous ne pouvons pas le garantir. L'intervention a été élaborée afin que vous puissiez mieux gérer votre problème de santé. Votre douleur pourra possiblement diminuer et votre qualité de vie pourrait s'améliorer. L'expérience des chercheurs dans le domaine a démontré que ce type d'intervention pouvait être efficace afin de rejoindre les objectifs mentionnés précédemment.

Par ailleurs, les informations découlant de ce projet de recherche pourraient contribuer à l'avancement des connaissances dans le traitement interdisciplinaire de la fibromyalgie et de la lombalgie.

ALTERNATIVE À LA PARTICIPATION AU PROJET DE RECHERCHE

Vous n'êtes pas obligé de participer à ce projet de recherche pour être traité pour votre fibromyalgie ou votre lombalgie simple. Votre médecin traitant pourra vous parler d'autres possibilités de traitement à base de médicaments ou de traitements de réadaptation.

PARTICIPATION VOLONTAIRE ET POSSIBILITÉ DE RETRAIT DU PROJET DE RECHERCHE

Votre participation à ce projet de recherche est volontaire. Vous êtes donc libre de refuser d'y participer. Vous pouvez également vous retirer de ce projet à n'importe quel moment, sans avoir à donner de raisons, en faisant connaître votre décision au chercheur responsable du projet ou à l'un de ses assistants. Les enregistrements ne

CÉR chez l'humain du CHUS
APPROUVÉ 26 nov 09 [illegible]

seront cependant pas détruits à moins que vous ne le demandiez. Si la technologie nous le permet, dans l'éventualité de votre retrait, nous pourrons brouiller votre visage.

Votre décision de ne pas participer à ce projet de recherche ou de vous en retirer n'aura aucune conséquence sur la qualité des soins et des services auxquels vous avez droit ou sur vos relations avec le chercheur responsable du projet et les autres intervenants.

Toute nouvelle connaissance acquise durant le déroulement de l'étude qui pourrait affecter votre décision de continuer d'y participer vous sera communiquée sans délai.

CONFIDENTIALITÉ

Durant votre participation à ce projet, le chercheur responsable du projet ainsi que son personnel recueilleront et consigneront dans un dossier de recherche les renseignements vous concernant. Seuls les renseignements nécessaires pour répondre aux objectifs scientifiques de l'étude seront recueillis.

Tous les renseignements recueillis au cours du projet demeureront strictement confidentiels dans les limites prévues par la loi. Les données en elles-mêmes ou combinées aux données provenant d'autres projets, pourront être partagées dans le cadre du *Programme Accord.* Afin de préserver votre identité et la confidentialité de ces renseignements, vous ne serez identifié que par un numéro de code. La clé du code reliant votre nom à votre dossier de recherche sera conservée par la chercheure responsable du projet (Patricia Bourgault) dans un local barré.

Les chercheurs responsables du projet utiliseront les données à des fins de recherche dans le but de répondre aux objectifs scientifiques du projet décrits dans le formulaire d'information et de consentement.

Les données pourront être publiées dans des revues scientifiques ou partagées avec d'autres personnes lors de discussions scientifiques. Aucune publication ou communication scientifique ne renfermera quoi que ce soit qui puisse permettre de vous identifier.

À des fins de surveillance et de contrôle, votre dossier de recherche pourra être consulté par une personne mandatée par le Comité d'éthique de la recherche en santé chez l'humain du CHUS ou par l'établissement, par une personne mandatée par des organismes publics autorisés. Toutes ces personnes et ces organismes adhèrent à une politique de confidentialité.

À des fins de protection, notamment afin de pouvoir communiquer avec vous rapidement vos noms et prénoms, vos coordonnées et la date de début et de fin de votre participation au projet, seront conservés pendant la durée de l'étude dans un répertoire maintenu par le chercheur, lequel sera gardé dans un classeur fermé à clé.

Vous avez le droit de consulter votre dossier de recherche pour vérifier les renseignements recueillis et les faire rectifier au besoin et ce, aussi longtemps que le

CÉR chez l'humain du CHUS
APPROUVÉ 26 [illegible]

chercheur responsable du projet ou l'établissement détiennent ces informations. Cependant, afin de préserver l'intégrité scientifique de l'étude, vous pourriez n'avoir accès à certaines de ces informations qu'une fois l'étude terminée.

COMPENSATION

Vous n'aurez rien à débourser pour participer aux Écoles interactionnelles de fibromyalgie ou de lombalgie évaluées dans ce projet de recherche. Cependant, les frais encourus comme le transport sont à votre charge mais le stationnement vous sera remboursé lors de la visite initiale et ensuite à la fin des ÉI.

DROITS DU SUJET ET INDEMNISATION EN CAS DE PRÉJUDICE

Si vous deviez subir quelque préjudice que ce soit dû à votre participation au projet de recherche, vous êtes protégé par les lois en vigueur au Québec. En acceptant de participer à cette étude, vous ne renoncez à aucun de vos droits ni ne libérez les chercheurs ou l'établissement où se déroule ce projet de recherche de leurs responsabilités civile et professionnelle.

FINANCEMENT DU PROJET DE RECHERCHE

Les chercheurs ont reçu des fonds des Instituts de recherche en santé du Canada pour mener à bien ce projet de recherche. Les fonds reçus couvrent les frais reliés à ce projet de recherche.

PERSONNES-RESSOURCES

Si vous avez des questions concernant le projet de recherche ou si vous avez des questions sur l'intervention qui vous est offerte dans le cadre de ce projet de recherche ou si vous croyez que vous éprouvez un problème de santé relié à votre participation au projet de recherche, vous pouvez communiquer avec la chercheure responsable du projet de recherche, Patricia Bourgault, au 819 346-1110 poste 12885.

Pour toute question concernant vos droits en tant que sujet participant à ce projet de recherche ou si vous avez des plaintes ou des commentaires à formuler vous pouvez communiquer avec la Commissaire aux plaintes et à la qualité du CHUS au numéro suivant : 819-346-1110, poste 14525.

SURVEILLANCE DES ASPECTS ÉTHIQUES

Le Comité d'éthique de la recherche en santé chez l'humain du CHUS a approuvé ce projet de recherche et en assure le suivi. De plus, nous nous engageons à lui soumettre pour approbation toute révision et toute modification apportée au protocole de recherche ou au formulaire d'information et de consentement.

Si vous désirez rejoindre l'un des membres de ce comité vous pouvez communiquer avec le Service de soutien à l'éthique de la recherche du CHUS au numéro 819-346-1110, poste 12856.

CÉR chez l'humain du CHUS
APPROUVÉ 26 [illegible]

CONSENTEMENT

Je déclare avoir lu le présent formulaire d'information et de consentement, particulièrement quant à la nature de ma participation au projet de recherche et l'étendue des risques qui en découlent. Je reconnais qu'on m'a expliqué le projet, qu'on a répondu à toutes mes questions et qu'on m'a laissé le temps voulu pour prendre une décision.

Je consens librement et volontairement à participer à ce projet.

J'accepte que l'on informe mon médecin *traitant / de famille* de ma participation à cette étude.

☐ **OUI** ☐ **NON**

Nom du médecin traitant et clinique : ______________________________

______________________	______________________	__________
Nom du participant /participante (lettres moulées)	*Signature du participant/participante*	*Date*
______________________	______________________	__________
Nom du témoin (lettres moulées)	*Signature du témoin*	*Date*
______________________	______________________	__________
Nom de la personne qui obtient le consentement (lettres moulées)	*Signature de la personne qui obtient le consentement*	*Date*

ENGAGEMENT DU CHERCHEUR

Je certifie qu'on a expliqué au sujet de recherche les termes du présent formulaire d'information et de consentement, que j'ai répondu aux questions que le sujet de recherche avait à cet égard et que j'ai clairement indiqué qu'il demeure libre de mettre un terme à sa participation, et ce, sans préjudice.

Je m'engage à respecter ce qui a été convenu au formulaire d'information et de consentement et à en remettre copie signée au sujet de recherche.

______________________	______________________	__________
Nom du chercheur (lettres moulées)	*Signature du chercheur*	*Date*

CÉR chez l'humain du CHUS
APPROUVÉ 26 nov 09 [illegible]

TABLEAU DÉTAILLÉ DES ÉCOLES INTERACTIONNELLES

Sessions	Contenus ÉIF/EIL
SÉANCE 1<br><br>CONTRAT	• Présentation de la fibromyalgie ou de la lombalgie.<br>• Présentation de chaque étape de l'école.<br>• Présentation des membres du groupe (nom, occupation, début symptômes, brève histoire de la maladie).<br>• Formulation d'un contrat : (a) établir trois objectifs personnels mesurables à atteindre à la fin de l'école, (b) déterminer le changement minimal acceptable visé à la fin de l'intervention (c) définir le temps consacré aux activités découlant de l'école (45min/j, 6 fois/sem.).<br>• Apprentissage de la respiration diaphragmatique (position assise).<br>• Tâche : 3 fois par jour faire 3 respirations diaphragmatiques (au minimum).
SÉANCE 2<br>SYMPTÔME	• Retour sur la rencontre précédente.<br>• Présentation des symptômes de la FM ou de la LB et des quatre symptômes qui seront touchés pendant l'école : la douleur, la fatigue, le sommeil et le stress.<br>• Identification des stratégies des participants qui sont efficaces pour améliorer la qualité du **sommeil**.<br>• Compléter avec les autres stratégies.<br>• Tâche : essayer deux stratégies pour favoriser le sommeil et compléter pendant la semaine le carnet de route (balle pression, marche selon ma capacité 3 fois/sem.).
SÉANCE 3<br>PRÉPARATION PHYSIQUE	• Retour sur la rencontre précédente.<br>• Rationnel de l'activité physique et son influence sur les symptômes.<br>• Test physique.<br>• Identification des stratégies des participants qui sont efficaces pour la **douleur**.<br>• Compléter avec les autres stratégies.<br>• Tâche : marche selon ma capacité 3 fois par semaine, poursuivre les autres stratégies, pratique routine motrice 6 fois/semaine et compléter le carnet de route pendant la semaine.
SÉANCE 4<br>GESTION DU STRESS	• Retour sur la rencontre précédente.<br>• Routine motrice<br>• Rationnel de la force mental sur la modulation de la douleur ainsi que sur la gestion du stress.<br>• Pratique d'une technique de relaxation active.<br>• Identification des stratégies des participants qui sont efficaces pour gérer le **stress**.<br>• Compléter avec les autres stratégies.<br>• Tâche : relaxation 3 fois par semaine, poursuivre les autres stratégies et compléter pendant la semaine le carnet de route.
SÉANCE 5<br>GESTION DES CAPACITÉS<br>(ÉIF)	• Retour sur la rencontre précédente.<br>• Pratique de la routine en classe.<br>• Apprendre à : (a) identifier ses limites, (b) respecter ses limites, (c) valoriser ses capacités (principe d'économie d'énergie, conservation de l'énergie, ergonomie).et d) pourquoi et comment bien se nourrir, avec une emphase sur le guide alimentaire canadien.<br>• Identification des stratégies des participants qui sont efficaces pour diminuer la **fatigue (augmentation de l'énergie)**.<br>• Essai de nouveaux aliments santé<br>• Compléter avec les autres stratégies.<br>• Tâche : appliquer deux des stratégies de gestion des capacités et manger de façon équilibrée et intégrer deux nouveaux aliments, poursuivre les autres stratégies et compléter pendant la semaine le carnet de route, poursuivre les autres stratégies et

CÉR chez l'humain du CHUS
APPROUVÉ 26 nov 09 [illegible]

	compléter pendant la semaine le carnet de route.<br>• Pratique d'une technique de relaxation passive.
**ANATOMIE (ÉIL)**	• Retour sur la rencontre précédente.<br>• Comprendre l'anatomie et le fonctionnement de la colonne vertébrale et adapter son environnement.<br>• Pratiques de positions à adopter.<br>• Identification des stratégies de mobilisation qui sont efficaces pour diminuer la **fatigue (augmentation de l'énergie)**..<br>• Compléter avec les autres stratégies.<br>• Tâche : appliquer deux des stratégies de mobilisation iet ntégrer deux nouvelles positions, poursuivre les autres stratégies et compléter pendant la semaine le carnet de route, poursuivre les autres stratégies et compléter pendant la semaine le carnet de route
**PAUSE 1 SEMAINE**	• Travail autonome.
**SÉANCE 6 CHRONICITÉ**	• Retour sur la rencontre précédente.<br>• Pratique de la routine motrice en classe.<br>• Modulation de la douleur, vivre avec la douleur chronique et les conséquences indésirables d'une guérison (stratégie, outil de gestion).<br>• Poursuite des tâches précédentes et compléter pendant la semaine le carnet de route.<br>• Technique de relaxation (*focussing*)
**PAUSE 1 SEMAINE**	• Travail autonome.
**SÉANCE 7 TRAITEMENTS**	• Retour sur la rencontre précédente.<br>• Routine motrice en classe.<br>• Rationnel des traitements pharmacologiques et non pharmacologiques et de la chirurgie (ÉIL seulement).<br>• Poursuite des tâches précédentes et compléter pendant la semaine le carnet de route.
**PAUSE 2 SEMAINES**	• Travail autonome.
**SÉANCE 8 CONDITION INITIALE**	• Retour sur la rencontre précédente.<br>• Discussion du cheminement parcouru durant l'école.<br>• Réévaluation du temps à consacrer à l'activité physique, la relaxation et les autres stratégies pendant les semaines à venir.<br>• Diplôme<br>• Poursuite des tâches précédentes et compléter pendant les semaines le carnet de route.
**SÉANCE 9 RELANCE (6 MOIS PLUS TARD)**	• Retour sur les semaines précédentes.<br>• Discussion du cheminement parcouru depuis la fin de l'école.<br>• Discussion des stratégies appliquées et qui fonctionnent.<br>• Ajustement des routines motrices individualisées<br>• Poursuite des tâches précédentes.

CÉR chez l'humain du CHUS
APPROUVÉ 26 [illegible] 05 [illegible]

À la fin de l'étude :

CONSENTEMENT À UTILISER L'ENREGISTREMENT POUR LA FORMATION

Je consens librement et volontairement à ce que les enregistrement de l'ÉI à laquelle j'ai participé servent à la formation de futurs intervenants.

Je comprends que ces enregistrements ne seront utilisés que pour la formation.

☐ **OUI** ☐ **NON**

______________________	______________________	__________
Nom du participant /participante (lettres moulées)	*Signature du participant/participante*	*Date*
______________________	______________________	__________
Nom du témoin (lettres moulées)	*Signature du témoin*	*Date*
______________________	______________________	__________
Nom de la personne qui obtient le consentement (lettres moulées)	*Signature de la personne qui obtient le consentement*	*Date*

Annexe 9

Formulaire d'information et de consentement à la recherche
pour le conjoint

CENTRE HOSPITALIER UNIVERSITAIRE DE SHERBROOKE
Hôpital Fleurimont, 3001, 12e Avenue Nord, Fleurimont (Québec) J1H 5N4
Hôtel-Dieu, 580, rue Bowen Sud, Sherbrooke (Québec) J1G 2E8
Téléphone : (819) 346-1110

FORMULAIRE D'INFORMATION ET DE CONSENTEMENT A LA RECHERCHE

Titre du projet: **DÉVELOPPEMENT, IMPLANTATION ET ÉVALUATION D'INTERVENTIONS DE TYPE «ÉCOLE INTERACTIONNELLE» ADAPTÉES À DES PERSONNES SOUFFRANT DE FIBROMYALGIE OU DE LOMBALGIE - CONJOINT**

Numéro et date du projet ***Programme Accord – Volet 3A***

Organisme subventionnaire Institut de recherche en santé du Canada (IRSC)

Chercheurs principaux:
Patricia Bourgault, inf., Ph.D., École des sciences infirmières, FMSS, Université de Sherbrooke
Serge Marchand, Ph.D., Service de neurochirurgie, FMSS, Université de Sherbrooke

Chercheurs associés :
Manon Choinière, Ph.D., Centre de recherche du Centre hospitalier de l'Université de Montréal
Dominique Dion, M.D., M.Sc., Centre de recherche du Centre hospitalier de l'Université de Montréal
Christian Cloutier, M.D., Service de neurochirurgie, Centre hospitalier universitaire de Sherbrooke
Sylvie Lamoureux, M.A. Ps., Clinique multidisciplinaire de douleur chronique, Centre hospitalier universitaire de Sherbrooke

Étudiante :
Emilie Lagueux, erg., étudiante au programme de maîtrise en sciences cliniques de l'Université de Sherbrooke

Nous sollicitons votre participation à un projet de recherche. Cependant, avant d'accepter de participer à ce projet, veuillez prendre le temps de lire, de comprendre et de considérer attentivement les renseignements qui suivent. Si vous acceptez de participer au projet de recherche, vous devrez signer le consentement à la fin du présent document et nous vous en remettrons une copie pour vos dossiers.

Ce formulaire d'information et de consentement vous explique le but de ce projet de recherche, les procédures, les avantages et les inconvénients, de même que les personnes avec qui communiquer au besoin. Il peut contenir des mots que vous ne comprenez pas. Nous vous invitons à poser toutes les questions nécessaires au chercheur responsable du projet ou aux autres personnes affectées au projet de recherche et à leur demander de vous expliquer tout mot ou renseignement qui n'est pas clair.

Pour de plus amples renseignements, vous pouvez contacter Patricia Bourgault au 819 346-1110 poste 12885 du lundi au vendredi de 8h à 17h.

NATURE ET OBJECTIFS DU PROJET DE RECHERCHE

Il existe différentes possibilités de traitement pour la fibromyalgie (FM) et la lombalgie (maux de dos) (LB). Plusieurs de ces traitements n'atteignent pas les objectifs visés notamment face à la réduction de la douleur, l'amélioration du sommeil et de la qualité de vie. Les traitements sont le plus souvent à base de médicaments associés ou non à une composante physique ou psychologique. Il est cependant reconnu scientifiquement que la combinaison de différentes approches donnent les meilleurs résultats surtout si l'intervention est interdisciplinaire (divers professionnels de la santé travaillant au même objectif). Le but de la présente étude est d'évaluer l'efficacité à court et à long terme d'interventions interdisciplinaires de type École interactionnelle de fibromyalgie (ÉIF) et de lombalgie (ÉIL) pour réduire la sévérité de la douleur chez des personnes souffrant de ces syndromes de douleur chronique.

De plus, comme la douleur peut avoir un impact sur la famille et les proches, nous évaluerons également si le fait de participer à cette intervention a un impact positif chez le conjoint. Plus précisément, il s'agira de voir si l'intervention permet d'augmenter la qualité de vie, de diminuer les niveaux d'anxiété et de dépression et d'augmenter le sentiment d'efficacité et le niveau de santé perçus chez ceux-ci.

DÉROULEMENT DU PROJET DE RECHERCHE

Cette étude se déroulera à l'École de réadaptation de l'Université de Sherbrooke (bâtiment annexé au Centre hospitalier universitaire de Sherbrooke à Fleurimont) à l'automne 2009 et au printemps 2010. L'étude comporte deux groupes un appelé groupe intervention suivra l'École à l'automne. Le groupe témoin, verra sa participation à l'École interactionnelle retardée de 6 mois mais elle sera en tout point identique à celle décrite précédemment. C'est le hasard qui déterminera à quel groupe votre conjoint sera assigné. Votre participation implique de répondre à des questionnaires.

Lors la première séance de l'École, nous recueillerons des données sociodémographiques et personnelles (sexe, année de naissance, ethnicité, langue première, scolarité, condition de vie actuelle, statut civil, statut d'emploi, revenu familial, satisfaction en regard du revenu, principale source de revenu, type de lien avec la personne FM ou LB, durée de l'aide apportée et le type d'aide apportée).

Par la suite, au même moment que votre conjoint participant aux ÉI (3, 6, 9 et 15 mois), nous vous demanderons de compléter les questionnaires listés ci-dessous. Il vous prendra environ 30 minutes pour les compléter.

- Qualité de vie
- Anxiété
- Inventaire de dépression
- Échelle de satisfaction de la relation avec le proche
- Échelle de perception de la santé
- Échelle de perception de l'efficacité personnelle
- Attente face à l'intervention pour votre conjoint
- Attachement amoureux
- Satisfaction conjugale
- Communication

COLLABORATION DU SUJET DE RECHERCHE

Votre participation implique que complétiez les questionnaires présentés précédemment.

INCONVÉNIENTS POUVANT DÉCOULER DE LA PARTICIPATION DU SUJET AU PROJET DE RECHERCHE

Les questionnaires ne causent aucun inconvénient sinon le temps nécessaire pour les compléter soit environ 30 minutes.

AVANTAGES POUVANT DÉCOULER DE LA PARTICIPATION DU SUJET AU PROJET DE RECHERCHE

Il se peut que vous retiriez un bénéfice personnel de votre participation à ce projet de recherche en comprenant mieux le problème de santé de votre conjoint, mais nous ne pouvons pas le garantir. Par ailleurs, les informations découlant de ce projet de recherche pourraient contribuer à l'avancement des connaissances dans le traitement interdisciplinaire de la fibromyalgie et de la lombalgie ainsi que sur l'impact de cette intervention sur le conjoint.

PARTICIPATION VOLONTAIRE ET POSSIBILITÉ DE RETRAIT DU PROJET DE RECHERCHE

Votre participation à ce projet de recherche est volontaire. Vous êtes donc libre de refuser d'y participer. Vous pouvez également vous retirer de ce projet à n'importe quel moment, sans avoir à donner de raisons, en faisant connaître votre décision au chercheur responsable du projet ou à l'un de ses assistants. Cela n'empêchera pas votre conjoint de continuer à participer.

Toute nouvelle connaissance acquise durant le déroulement de l'étude qui pourrait affecter votre décision de continuer d'y participer vous sera communiquée sans délai.

CONFIDENTIALITÉ

Durant votre participation à ce projet, le chercheur responsable du projet ainsi que son personnel recueilleront et consigneront dans un dossier de recherche les renseignements vous concernant. Seuls les renseignements nécessaires pour répondre aux objectifs scientifiques de l'étude seront recueillis.

Tous ces renseignements recueillis au cours du projet demeureront strictement confidentiels dans les limites prévues par la loi. Afin de préserver votre identité et la confidentialité de ces renseignements, vous ne serez identifié que par un numéro de code. La clé du code reliant votre nom à votre dossier de recherche sera conservée par la chercheure responsable du projet (Patricia Bourgault).

Le chercheur responsable du projet utilisera les données à des fins de recherche dans le but de répondre aux objectifs scientifiques du projet décrits dans le formulaire d'information et de consentement.

Les données pourront être publiées dans des revues médicales ou partagées avec d'autres personnes lors de discussions scientifiques. Aucune publication ou communication scientifique ne renfermera quoi que ce soit qui puisse permettre de vous identifier.

À des fins de surveillance et de contrôle, votre dossier de recherche pourra être consulté par une personne mandatée par le Comité d'éthique de la recherche en santé chez l'humain du CHUS ou par l'établissement, par une personne mandatée par des organismes publics autorisés. Toutes ces personnes et ces organismes adhèrent à une politique de confidentialité.

À des fins de protection, notamment afin de pouvoir communiquer avec vous rapidement vos noms et prénoms, vos coordonnées et la date de début et de fin de votre participation au projet, seront conservés pendant un an dans un répertoire sécurisé maintenu par le chercheur.

Vous avez le droit de consulter votre dossier de recherche pour vérifier les renseignements recueillis et les faire rectifier au besoin et ce, aussi longtemps que le chercheur responsable du projet ou l'établissement détiennent ces informations. Cependant, afin de préserver l'intégrité scientifique de l'étude, vous pourriez n'avoir accès à certaines de ces informations qu'une fois l'étude terminée.

COMPENSATION

Les frais encourus comme le transport et le stationnement sont à votre charge.

DROITS DU SUJET ET INDEMNISATION EN CAS DE PRÉJUDICE

Si vous deviez subir quelque préjudice que ce soit dû à votre participation au projet de recherche, vous êtes protégé par les lois en vigueur au Québec. En acceptant de participer à cette étude, vous ne renoncez à aucun de vos droits ni ne libérez les chercheurs ou l'établissement où se déroule ce projet de recherche de leurs responsabilités civile et professionnelle.

FINANCEMENT DU PROJET DE RECHERCHE

Les chercheurs ont reçu des fonds des Instituts de recherche en santé du Canada pour mener à bien ce projet de recherche. Les fonds reçus couvrent les frais reliés à ce projet de recherche.

PERSONNES-RESSOURCES

Si vous avez des questions concernant le projet de recherche ou si vous avez des questions sur l'intervention qui vous est offerte dans le cadre de ce projet de recherche ou si vous croyez que vous éprouvez un problème de santé relié à votre participation au projet de recherche, vous pouvez communiquer avec la chercheure responsable du projet de recherche, Patricia Bourgault, au 819 346-1110 poste 12885.

Pour toute question concernant vos droits en tant que sujet participant à ce projet de recherche ou si vous avez des plaintes ou des commentaires à formuler vous pouvez communiquer avec la Commissaire aux plaintes et à la qualité du CHUS au numéro suivant : 819-346-1110, poste 14525.

SURVEILLANCE DES ASPECTS ÉTHIQUES

Le Comité d'éthique de la recherche en santé chez l'humain du CHUS a approuvé ce projet de recherche et en assure le suivi. De plus, nous nous engageons à lui soumettre pour approbation toute révision et toute modification apportée au protocole de recherche ou au formulaire d'information et de consentement.

Si vous désirez rejoindre l'un des membres de ce comité vous pouvez communiquer avec le Service de soutien à l'éthique de la recherche du CHUS au numéro 819-346-1110, poste 12856.

CONSENTEMENT

Je déclare avoir lu le présent formulaire d'information et de consentement, particulièrement quant à la nature de ma participation au projet de recherche et l'étendue des risques qui en découlent. Je reconnais qu'on m'a expliqué le projet, qu'on a répondu à toutes mes questions et qu'on m'a laissé le temps voulu pour prendre une décision.

Je consens librement et volontairement à participer à ce projet.

______________________	______________________	__________
Nom du participant /participante<br>(lettres moulées)	*Signature du participant/participante*	*Date*

______________________	______________________	__________
Nom du témoin<br>(lettres moulées)	*Signature du témoin*	*Date*

______________________	______________________	__________
Nom de la personne qui obtient le consentement (lettres moulées)	*Signature de la personne qui obtient le consentement*	*Date*

ENGAGEMENT DU CHERCHEUR

Je certifie qu'on a expliqué au sujet de recherche les termes du présent formulaire d'information et de consentement, que j'ai répondu aux questions que le sujet de recherche avait à cet égard et que j'ai clairement indiqué qu'il demeure libre de mettre un terme à sa participation, et ce, sans préjudice.

Je m'engage à respecter ce qui a été convenu au formulaire d'information et de consentement et à en remettre copie signée au sujet de recherche.

______________________	______________________	__________
Nom du chercheur (lettres moulées)	*Signature du chercheur*	*Date*

Annexe 10

Autorisation de financement des IRSC

1+1 Canadian 1 nstitutes of Health Research — Instituts de recherche en santé du Canada

AUTHORIZATION FOR FUNDING

CIHR (Canadian Institutes of Health Research) has approved funding as detailed below. Subject to the approbation of funding by Parliament, these funds will be made available to the business officer at the indicated institution for disbursement.

AUTORISATION DE FINANCEMENT

IRSC (Instituts de recherche en santé du Canada) vous accorde les fonds qu'indiqué ci-dessous. Suivant l'affectation des crédits par le Parlement du Canada, les fonds seront mis à la disposition du trésorier de l'établisseme indiqué qui s'occupera des versements.

200702CAH-175475-CAH-CFCK-17834

14/03/2

Institution Paid/Établissement chargé d'administrer les fonds: Institut de cardiologie de Montréal

Recipient(s)/Bénéficiaire(s): CHOINIÈRE, Manon
Anesthésiologie
Faculté de médecine
Université de Montréal

Docteur Serge MARCHAND, Dr. Terrence Joseph MONTAGUE, Docteure Dominique DION

Program/Programme: Subvention d'équipe: Alliances communautaires pour la recherche en santé et l'échange des connaissances sur la douleu
Nouvelle subvention
ln Partnership with/En partenariat avec: AstraZeneca Canada Inc.; CIHR - Inst of Musculoskel Hlth &Arth; CIHR - Inst of Neurosc Mental Hlth &A
Agreement number / Numéro de l'entente: 00599 - 000

Primary Institutellnstitut principal: Appareil locomoteur et arthrite

Project TitlelTitre du projet:

Le Programme ACCORD - Application Concertée des Connaissances et des Ressources en Douleur

Co-investigator(s) & Associates/Supervisor(s)/HostlCo-chercheur(s)/Directeur(s) de recherche/Hôte:

Professeur Patricia BOURGAUL T, Dr. Celeste JOHNSTON, Docteure Lyne LALONDE, Professeure Diane LAMARRE, et coll.

PAYMENT DETAILS/DÉTAILS DES VERSEMENTS — Funding Reference Number/ No. de Référence du financement: CAH - 86787

Period / Période	Type	Amount by Type / Montant par type	Total by Fiscal Year / Total par exercice	
01/01/2008 à 31/03/2008	Fonctionnement	$125,000	$125,000	2007-08
01/04/2008 à 31/03/2009	Fonctionnement	$499,706	$499,706	2008-09
01/04/2009 à 31/03/2010	Fonctionnement	$498,893	$498,893	2009-10
01/04/2010 à 31/03/2011	Fonctionnement	$497,778	$497,778	2010-11
01/04/2011 à 31/03/2012	Fonctionnement	$494,787	$494,787	2011-12
01/04/2012 à 31/12/2012	Fonctionnement	$373,300	$373,300	2012-13

i Progress Report Required: li Rapport des progrès réalisés requis: 01/01/2011

Application to Renew Funding Required: Demande de renouvellement des fonds requis: Non Renouvelable

NOTES:

Numéro de l'entente #599-000

La valeur totale de cette subvention est de 2,489,464$. Sur ce total, 1,789,464$ proviennent de l'Institut de l'appareil locomoteur et de l'arthrite, 450,000$ proviennent de l'Institut des Neurosciences, da la santé mentale et des toxicomanies et 250,000$ proviennent de AstraZeneca.

Les IRSC requièrent que leur contribution à votre projet de recherche soit reconnue dans toutes les présentations écrites et orales des résultats de votre 1 recherche, y compris les articles à caractère scientifique, les communiqués de presse, les conférences de presse, les conférences publiques entrevues avec les médias. Veuillez consulter les lignes directrices des IRSC sur les communications publiques ci-joint pour plus de détails sur les exigences en matière de communication publique et la mention de l'aide des IRSC.

Vous avez reçu ce financement parce que vos collègues ont gracieusement mis leur temps à la disposition des IRSC pour examiner votre demande. Les IRSC espèrent que, en tant que bénéficiaire du financement des IRSC, vous prendrez part aux activités d'examen par les pairs si vous y êtes invité.

Richard R. Snell, Ph.D.
Directeur adjoint, Direction des programmes de création des connaissances
Portefeuille de la recherche
