# Supplementary material for: Multicomponent Interdisciplinary Group Intervention for Self-Management of Fibromyalgia: A Mixed-Methods Randomized Controlled Trial
Source: PLoS One. 2015 May 15;10(5):e0126324. doi: 10.1371/journal.pone.0126324 (PMC4433106; doi:10.1371/journal.pone.0126324)
Supplement: S2 Protocol — (PDF) [file pone.0126324.s004.pdf]

ENGLISH VERSION OF THE RESEARCH PROTOCOL METHODOLOGY

4. STUDY METHODOLOGY

4.1 Research design

In order to meet the previously described objectives, a randomized control trial with repeated measures will be used. Interactional schools (the intervention) will be subjected to a multi-center randomised control trial (Study sites: *Université de Sherbrooke* and *Université du Québec en Abitibi-Témiscamingue*). A multi-centre study will help establish the necessary conditions in order to standardize the procedures for an eventual implantation of the intervention in other centers all across the province of Quebec.

Participants suffering from fibromyalgia or low back pain will be randomized in all study centers with the help of a random numbers tables. They will either be assigned to the Intervention group or to a waitlist (Control group). The stratified randomization method will be used for the randomization of participants in the intervention and control groups based on their pain intensity levels of moderate (4-6/10 on a numeric scale ranging from 0 = no pain to 10 = worst possible pain) or severe (≥ 7/10 on the same numeric scale) as experienced in the seven days prior to the initial evaluation. Participants will then be stratified based on gender. It should be noted that the fibromyalgia groups (intervention and control) will only consist of women due to this disorder 6/1 female/male ratio (McNally *et al*., 2006). Such a strategy will help eliminate the potential variability sources related to gender.

Participants receiving the intervention will be followed over a 12 months period while those in the control group will be followed for 6 months so that they can receive the intervention within an ethical delay.

4.2 Population

Adult individuals in the Sherbrooke or Rouyn-Noranda regions who suffer from fibromyalgia or chronic low back pain.

4.3 Selection criteria

An equal number of participants will be recruited in all study sites based on the following inclusion/exclusion criteria:

4.3.1 Inclusion criteria. Participants will need to meet all the following criteria in order to be included in the study:

- o Have a medical diagnostic of primary fibromyalgia for at least 6 months and established based on the American College of Rheumatology criteria (Wolfe *et al*., 1990) —i.e., 11/18 pain sensibility points from pressure equivalent to 4kg (Wolfe *et al*., 1990).

OR

Have a medical diagnostic of non specific low back pain for at least 6 months and established based on Chou's (2007) criteria.

- o Aged between 18 and 65 years old.
- o Being able to read, understand and able to complete questionnaires written in French.
- o Report a level of pain of moderate intensity ≥ 4/10 as experienced in the seven days prior to the initial evaluation.
- o Accept to be randomly assigned to one or the other experimental conditions.
- o Being interested and motivated by the type of proposed intervention (client vs. tourist: Here a client represent a person with fibromyalgia or low back pain who actively ask for help and meet the medical criteria. The therapeutic tactic aims at evaluating if the person is "client" or, in the case of a "tourist", help them become "client" (Barcellos de Souza, 2007, p.213).
- o Accept to participate in 9 weekly meetings over an 11-week period plus a follow-up visit.
- o Maintain a stable medical treatment for pain or abstain from using any analgesic over the course of the study.

4.3.2 Exclusion criteria. Participants will be excluded from the study if they meet any of the following criteria:

- o Pregnant or lactating.
- o Suffering from an active cancer or an uncontrolled disease of metabolic origin.
- o Suffering from chronic pain other than fibromyalgia or low back pain (e.g., rheumatoid arthritis, diabetic neuropathy, etc.).
- o Suffering from a major physical or psychological co-morbidity sufficiently severe to limit the participant's ability to participate in the study.
- o Having compensation issues with the CSST or the SAAQ.

Note that participants who start a new analgesic treatment over the course of the study will be excluded.

4.4 Study protocol and data collection

4.4.1 Training of the research and facilitators. Research assistant in all study centers will receive a serious training in order to insure the uniformity of the procedures used for the recruitment of participants and for the data collection. This will also be the case for the facilitators who will lead the intervention. Facilitators will all participate in the same training session that will be held at the Sherbrooke University. During this training session, the content of the interventions sessions, the intervention modes to be favored and the strategies to use in case of problems will be discussed at length. All

intervention's sessions will be filmed in order to insure uniformity between centers in the implantation of the program and in order to develop a video that will be used for the training of future facilitators in other rehabilitation centers of the province of Quebec where the intervention will be offered in the future.

4.4.2 Recruitment and data collection procedure.

Participants' recruitment will be accomplished with a non-probabilistic strategy where adds will be published in local newspapers of the regions of Sherbrooke and Rouyn-Noranda (see add in Annexe 1). Individuals interested in participating will be invited to communicate by phone with the research assistant. At the time of this phone call, the research assistant will do a pre-selection interview with a structured guide (Annexe 2) in order to determine if the individual meets the research selection criteria. If he or she does, the research assistant will explain the objectives and the flow of the study as well as what is expected of him or her. If the individual shows an interest, a first appointment will be scheduled in order to: confirmed by a physician the fibromyalgia or the non-specific low back pain diagnostic as well as to ensure that the individual is interested in the research project, is motivated to learn how to better manage pain (objective of the intervention) and the availability of the spouse to answer questions from different questionnaires. If the individuals is then selected for the study, he or she will read and sign the consent form (the spouse will also read and sign the consent form but this can occur at the time of the first intervention session), a basic evaluation will be done and they will complete the initial (T_0) self-reported measures from a questionnaire composed of validated scales. A 90-minute period should be enough to complete the initial evaluation (T_0) including the questionnaire. After this first meeting, participants will be randomized into the intervention or the control group with stratification by pain intensity and gender. Participants of the intervention group will be invited to a first school (intervention) session and participants of the control group will be informed of their later participation in the intervention.

Participants assigned to the intervention group will then start the program which include 9 weekly sessions over a 11-week period. The theme that will covered and the content of each sessions are described in Table 1. At the end of the last program session (T_1) that is 3 months post-randomization, at 3 months post intervention (T^2), at 6 months post-intervention (T_3) where there will be a follow-up and at 12 months post-intervention, participants of the intervention group will complete the same questionnaire as the one completed at the time of the initial evaluation. The total length of the intervention will thus be of 15 months for this group. As for the participants assigned to the control group, the same measures will be collected simultaneously. Measures immediately post-intervention will also be collected from the participants of the control group once they will have completed the intervention. The total length of the study will thus also be of 15 months for the control group (see Figure 1).

Figure 1 Study flow and data collection

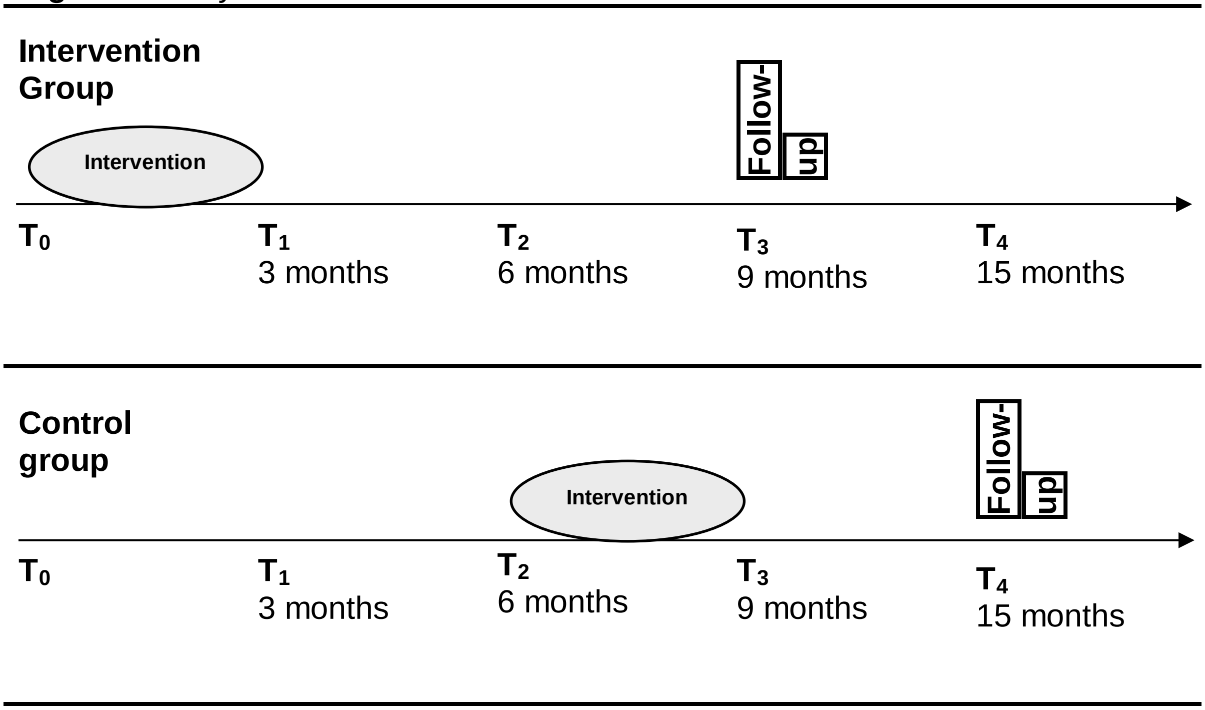

These measures will be collected at each measurement points (T_0 à T_4) from the participants as well as their spouse and that for both groups.

Note that measures will also be collected from participants' spouses at each measurement points with self-reported questionnaires. They will thus also read and sign an informed consent form before completing the questionnaire. The length of time estimated for a spouse to complete the questionnaire will be 45 minutes.

Most study data will be collected with self-administered questionnaires. The T_0, T_1, and T_3 questionnaires will be given to participants in person and will be completed on the spot while the T_2 and T_4 questionnaires will be sent to participants by mail (intervention group only). For the control group, only the T_1 questionnaire will be sent by mail. They will be invited to complete the questionnaire at home in the following week and return it in a pre-stamped envelope. One week after the sending of the postal questionnaire, the research assistant will call each participants in order to remind them of the importance of their participation in the study.

Due to the complexity associated with the measures specific to the preliminary economic analysis, the data on the use of healthcare services and on the loss of productivity will be collected over the phone by a research assistant. Telephone interviews of approximately 15 minutes will be planned in the same week as the self-administered questionnaire (T_0 to T_3 in the intervention group and T_0 to T_2 in the control group).

4.5 Variables and evaluation tools

4.5.1 Independent variables

The present research uses two independent variables, The first one refers to the intervention condition (i.e., intervention vs. Control). The second independent variable refers to the passing of time (i.e., before (T_0) and after the intervention where measures are collected at different times for the intervention group (T_1 - immediately post-intervention, T_2 - 6 months post-intervention, T_3 - 9 months post-intervention and T_4 - 15 months post intervention). For the control group, three measures will be collected before the intervention and two after the end of the intervention (see Figure 1).

The intervention follows the interdisciplinary model of the interactional back school (Charest, *et al.*, 1994) previously presented. As shown in Table 1, the intervention include 9 sessions. The firsts are weekly over a period of 5 weeks. Then there is one week of independent work between the session 5 and 7 followed by two weeks of independent work and finally the 8th session. The 9th session corresponds to the follow-up meeting. The sessions are held in groups of eight participants and two facilitators of different professional fields in order to facilitate the interdisciplinary approach. Ideally, one facilitator has a psychological training (e.g., psychologist, nurse, etc.) and the other has a physical training (e.g., ergotherapist, physiotherapist, etc.). Each sessions lasted two hours and thirty minutes including a fifteen minutes break for networking.

The 5th and 6th sessions of the intervention present different topics.

4.5.2 Dependent variables and measurement tools used with participants

The choice of dependent variables and measurement tools used in the present study were based on the recent recommendation from the IMPPACT group (Inititatives on Methods, Measurement, and Pain Assessement in Clinical Trials) (Brislin, 1986; Turk, Dworkin, Allen, Bellamy, Brandenburg, Carr *et al.*, 2003; Dworkin, Turk, Farrar, Haythornthwaite, Jensen, Katz *et al.*, 2005). The selection of the measurement tools was done while considering their psychometric qualities (e.g., validity, reliability, sensibility, etc.), the existence of valid French versions, and the existence of normative data when applicable. The selection was also guided by a desire to minimise the burden on participants. Finally, the choice of measures was based on the experience of the people in charge of the present project who have used a number of similar questionnaire measures in past studies including that of Barcellos de Souza (2007). Concerning choice of items to measure participants' demographic data, their medical history including that related to pain as well as the pharmacologic and non pharmacologic treatments they use to manage pain, they were from the Registre Quebec Douleur (Quebec Pain Registry) which was created in 2006. This registry is now used in the pain management expertise centers in the *Réseau Universitaire Intégré de Santé* (RUIS) of the province of Quebec including the CHUS (Sherbrooke University Health Center).

Table 2, reproduced in Annexe 3, lists each variable collected in the present study from the fibromyalgia and low back pain participants and when they will be collected. Regarding the variables collected at the initial evaluation, they can be grouped and summarized in the following way:

- ***Characteristics of pain*** including for instance its intensity measured with a numeric scale ranging from 0 to 10 where 0 = no pain and 10 = worst possible pain (Jensen and Karoly, 2001) as well as its impact on a number of daily life spheres (interference items from the Brief Pain Inventory (Cleeland and Ryan, 1994 ; Larue, Colleau, Brasseur and Cleeland, 1995 ; Tyler *et al*., 2002).

- ***Quality of sleep*** measured with the Chronic Pain Sleep inventory *(Kosinki, Janagap, Gajria, and Schein (2007)*.

- ***Pharmacologic and non pharmacologic treatments*** currently used in order to manage pain and other medical disorders.

- ***Usage of tobacco, alcohol and drugs.***

- ***Pain coping strategies***
    - ***Dramatisation tendency*** measured with the Pain Catastrophizing Scale (Sullivan, Bishop and Pivik, 1995 ; French, Vigneau, French, Cyr and Evans, 2004).
    - ***Pain coping strategies Questionnaire*** (CSQ-F) (*Coping strategies Questionnaire* Rosenstiel and Keefe, 1983, Irachbal *et al*., 2002).

- ***Depression level*** evaluated with the *Beck* Depression Inventory - V1 (Beck, Ward, Mendelson, Mock and Erbaugh, 1961 ; Gauthier, Thériault, Morin and Lawson, 1982).

- ***Health-related quality of life*** (*SF-12v2)* (Ware, Kosinski and Keller, 1996).

- ***Patient's expectations towards the intervention*** - i.e., expected changes about pain, daily functioning and quality of life *(Patient Global Impression of Change)* – categorical scale ranging from "considerably deteriorated" to "considerably improved" (Dworkin, Nagasako, Hetzel and Farrar, 2001 ; Farrar, 2003). A level of relief scale (0% = no relief, 100% = total relief) (Haythornthwaite and Fauerbach, 2001 ; Jensen, 2003) will also be used in order to measure the expectation of participants in terms of relief.

- ***Costs associated with pain*** (usage of healthcare resources and loss of productivity). See 4.5.4 for more details about this measure.

- ***Sociodemographic characteristics*** (gender, birth year, ethnicity, maternal language, education, current life conditions, marital status, employment status, etc.).

Depending on the study population :

- ***Fibromyalgia impact*** measured with the Fibromyalgia Impact Questionnaire (Burckhardt, Clark and Bennett, 1991 ; Perrot *et al*., 2003).

- ***Disabilities caused by low back pain*** evaluated with the *Échelle d'incapacité du dos du Québec* (Quebec back disabilities scale) (Kopec *et al*., 1996)

All previously described measures will be collected at T_0, T_1, T_2 T_3 and T_4 except those related to participants' expectations about the intervention that are only collected at T_0. These will be replaced by questions about global impression of change as perceived by participants regarding their pain, their functioning status and their quality of life with a categorical scale ranging from "considerably deteriorated" to "considerably improved" (*Patient Global Impression of Change)* (Dworkin, Nagasako, Hetzel and Farrar, 2001 ; Farrar, 2003). The degree of relief scale (0% = no relief, 100% = complete relief) (Haythornthwaite and Fauerbach, 2001 ; Jensen, 2003) will also be completed at each measurement points. Participants will have to rate their level of satisfaction towards the interactional school with a satisfaction scale in 6-point ranging from "very unsatisfying" to "very satisfying" (Haythornthwaite and Fauerbach, 2001). Regarding the sociodemographic data, only the questions related to employment status and sources of income will be repeated.

This section's questionnaires and measurement tools are presented in Annexe 4.

4.5.3 Dependent variables and measurement tools used with participants' spouses. (see **Table 3 Annexe 5**)

- ***Health related quality of life*** (*SF-12v2)* (Ware, Kosinski and Keller, 1996).

- Anxiety (*STAI)* (Spielberger *et al*., 1983; Spielberger *et al*., 1988; Gauthier and Bouchard, 1993; Spielberger *et al*., 2008)

- Beck Depression Inventory - V1 (Beck *et al*., 1961; Bourque and Beaudette, 1982, Beck, Epstein, Brown and Steer, 1988; Beck *et al*. 1996;).

- **Satisfaction of the relationship with the spouse.** Relationship with the spouse: Very satisfying, moderately satisfying, moderately unsatisfying, very unsatisfying

- Activities and attitudes about sexuality questionnaire Beaudoin, Carbonneau, Godbout, Bouchard and Sabourin (2007).

- **Perceived health** (Santé Québec, 1995)

Questions assessing the spouse's perception of his/her own health compared with the health of other people from the same age group.

- **Self-efficacy scale** (Bandura, 1977; Hébert *et al*., 2004).

General questionnaire about perceived self-efficacy adapted to the role of care giver. Self-efficacy is the conviction that one can create a positive impact and the conviction that one can do something that refers, in the present study, to the ability to act as care giver to one's spouse.

- ***Spouse's expectation about the intervention***—i.e., Expected changes about pain, daily functioning, and quality of life *(Patient Global Impression of Change)* – categorical scale ranging from "considerably deteriorated" to "considerably improved" (Dworkin, Nagasako, Hetzel and Farrar, 2001 ; Farrar, 2003). A perceived relief scale about the participants of the (0% = no relief, 100% = complete relief) (Haythornthwaite and Fauerbach, 2001 ; Jensen, 2003).

- **Personal and sociodemographic variables** (gender, year of birth, ethnicity, maternal language, education, current life conditions, marital status, employment status, primary source of income, type of relationship with the participant, cohabitation or not with the participants, length of time in the care giving role).

Questionnaires and measurement tools are presented in Annexe 6.
